# Therapeutic Potential of Anti-Obesity Drugs in Obesity-Associated Female Reproductive Dysfunction: Translating Mechanistic Evidence into Personalized Clinical Strategies

**DOI:** 10.3390/medicina62081445

**Published:** 2026-07-25

**Authors:** Fani-Niki Varra, Panagiotis Theodosis-Nobelos, Viktoria-Konstantina Varra, Michail Varras

**Affiliations:** 1 Department of Pharmacy, School of Health Sciences, Frederick University, Nicosia 1036, Cyprus; fannyvarra@gmail.com (F.-N.V.); hsc.np@frederick.ac.cy (P.T.-N.); 2 Medical School, Democritus University of Thrace, 6810 Alexandroupolis, Greece; 3 Department of Pharmacy, School of Health Sciences, University of Patras, University Campus, 26504 Patra, Greece; nadv.ars2016@gmail.com; 4 Division of Aesthetics and Cosmetic Science, Department of Biomedical Sciences, School of Health and Welfare Sciences, University of West Attica, Aigaleo, 12243 Athens, Greece; 5 Fourth Department of Obstetrics and Gynecology, ‘Elena Venizelou’ General and Maternity Hospital, Plateia Elenas Venizelou 2, Ampelokipoi, 11521 Athens, Greece

**Keywords:** anti-obesity agents, pharmacotherapy, female reproduction, obesity, weight loss, infertility, orlistat, exenatide, liraglutide, semaglutide, phentermine, topiramate, bupropion, naltrexone, metformin, exenatide, tirzepatide

## Abstract

Obesity is a multifactorial condition that profoundly affects female reproductive health through endocrine, metabolic, and inflammatory mechanisms that disrupt the hypothalamic–pituitary–gonadal (HPG) axis. Women with obesity frequently develop menstrual irregularities, anovulation, amenorrhea, infertility, polycystic ovary syndrome (PCOS), impaired endometrial receptivity, and adverse pregnancy outcomes. Central obesity and insulin resistance contribute to hyperinsulinemia, reduced sex hormone-binding globulin (SHBG) levels, hyperandrogenism, altered gonadotropin secretion, and impaired folliculogenesis, while adipokines such as leptin and chronic inflammation further impair ovarian steroidogenesis and ovulatory function. Obesity-related oxidative stress and lipotoxicity also negatively affect oocyte quality, embryo development, implantation, and assisted reproductive technology outcomes, increasing the risk of gestational diabetes, preeclampsia, miscarriage, and preterm birth. This review evaluates the therapeutic potential of pharmacological weight-loss therapies in obesity-associated female reproductive dysfunction. A comprehensive literature review was conducted using various databases, focusing on anti-obesity pharmacotherapy on obesity, infertility and fertility in reproductive-aged women. Evidence suggests that several FDA-approved and off-label anti-obesity agents, including orlistat, liraglutide, semaglutide, phentermine/topiramate, bupropion/naltrexone, metformin, exenatide, and tirzepatide, may improve reproductive outcomes primarily indirectly through weight reduction and metabolic improvement. GLP-1 receptor agonists, particularly liraglutide, semaglutide, and exenatide, appear especially promising, demonstrating beneficial effects on insulin sensitivity, menstrual regularity, ovulation, androgen levels, and pregnancy rates in women with PCOS. Tirzepatide, a dual GLP-1/GIP receptor agonist, has shown potent weight-loss and metabolic effects with potential indirect fertility benefits. Metformin improves insulin sensitivity and is widely used in PCOS to regulate androgen levels and restore ovulation, although its effects on pregnancy and live birth rates remain controversial. However, evidence for several agents remains limited, and concerns persist regarding reproductive safety during pregnancy. Overall, anti-obesity pharmacotherapy may represent an important adjunctive strategy for improving reproductive and metabolic health in women with obesity, although larger randomized clinical trials are still required.

## 1. Introduction

Obesity represents one of the most significant public health challenges worldwide, with a continuously increasing prevalence among women of reproductive age. It is a multifactorial, chronic metabolic disease associated with significant endocrine, inflammatory, and metabolic disturbances that affect multiple body systems [1,2,3]. Of particular importance is its impact on female reproductive health, as increased adipose tissue mass is associated with ovulatory dysfunction, menstrual irregularities, infertility, and an increased risk of pregnancy complications [4]. In this context, polycystic ovary syndrome (PCOS) constitutes a characteristic example of the interaction between obesity and reproductive dysfunction, where metabolic and hormonal disturbances reinforce one another, leading to a more complex clinical presentation [5,6].

In recent years, the pharmacological management of obesity has advanced significantly, offering new therapeutic options not only for weight reduction but also for the improvement of metabolic and reproductive parameters [7]. At the same time, there is growing interest in the use of anti-obesity medications in women with reproductive dysfunction, particularly in those with PCOS, infertility, or ovulatory disorders [8]. The relationship between weight loss and improvement in reproductive function is complex and multifactorial, involving both hormonal and metabolic mechanisms [9]. Within this framework, GLP-1 (Glucagon-Like Peptide-1) receptor agonists, such as liraglutide, semaglutide, and exenatide, have emerged as an important class of medications for the management of both type 2 diabetes mellitus and obesity. These agents enhance glucose-dependent insulin secretion, suppress glucagon release, delay gastric emptying, and reduce appetite through central nervous system pathways [10]. Collectively, these effects lead to improved glycemic control and substantial weight loss [10]. Thus, agents such as liraglutide, through its effects on weight reduction, improvement of insulin sensitivity, and reduction in hyperinsulinemia, indirectly contribute to the restoration of hormonal balance. These metabolic changes are associated with decreased androgen production, increased sex hormone-binding globulin (SHBG) levels, and improved ovarian function, resulting in enhanced menstrual regularity and ovulatory activity [10]. Similarly, in women with obesity and PCOS, semaglutide-induced weight loss can simultaneously decrease systemic inflammatory markers, which might subsequently lower ovarian androgen production and promote a more physiological restoration of gonadotropin secretion [11]. More recently, tirzepatide, a dual GLP-1 and glucose-dependent insulinotropic polypeptide (GIP) receptor agonist, has emerged as one of the most effective pharmacological options for the treatment of obesity and type 2 diabetes mellitus [12]. By simultaneously activating two incretin pathways, it produces greater weight loss and superior glycemic control compared with previous therapies. It also appears to improve insulin sensitivity, inflammatory status, and lipid metabolism [12]. Although evidence regarding its direct effects on female reproductive health remains limited, the substantial metabolic improvements it induces may indirectly enhance ovarian function and fertility, particularly in women with obesity and PCOS [13]. It is important to note that both tirzepatide and metformin are widely used in this context as off-label pharmacological treatments for obesity-related reproductive dysfunction, especially in women with PCOS. Metformin, a well-established biguanide, is one of the most extensively studied agents beyond its primary indication for type 2 diabetes mellitus. It acts mainly by reducing hepatic gluconeogenesis, improving peripheral insulin sensitivity, and activating AMP (adenosine monophosphate)-activated protein kinase (AMPK) [14]. Through these mechanisms, it improves glycemic control and may lead to modest weight reduction. Importantly, metformin has well-documented effects on female reproductive function, particularly in PCOS, where it can contribute to the regulation of menstrual cycles, the improvement of ovulation, and the reduction in androgen levels [15]. However, its effects on definitive reproductive outcomes, such as live birth rates, remain controversial [15,16]. Tirzepatide promotes significant weight loss, improves insulin sensitivity, and reduces hyperinsulinemia, all of which are key pathogenic factors in PCOS-associated anovulation and infertility. Furthermore, improved metabolic control may restore menstrual regularity, decrease androgen excess, and enhance ovulatory function. Since obesity and insulin resistance strongly impair reproductive outcomes, the metabolic benefits induced by tirzepatide may indirectly improve fertility potential. These effects explain its increasing clinical use despite the absence of formal reproductive indications [17,18,19].

Overall, the present review focuses on investigating the role of anti-obesity medications in obesity-related female reproductive dysfunction. Their metabolic and hormonal effects are examined, as well as their potential impact on fertility and reproductive outcomes. Available evidence suggests that the pharmacological treatment of obesity may in some cases substantially contribute to the improvement of reproductive health, although outcomes vary depending on the medication and the population studied. Finally, the need for further clinical studies is highlighted in order to achieve a better understanding of the mechanisms of action and to optimize the therapeutic approaches in women with obesity and reproductive disorders.

## 2. Search Strategy

A comprehensive search strategy was developed to identify the relevant literature on the therapeutic potential of anti-obesity drugs in obesity-associated female reproductive dysfunction. The aim was to capture evidence linking pharmacological weight-loss interventions with metabolic, endocrine, and reproductive outcomes in obese women of reproductive age, particularly those affected by obesity-related conditions such as PCOS, infertility, menstrual dysfunction, and adverse pregnancy outcomes. A systematic search was conducted in PubMed/MEDLINE, Embase, Web of Science Core Collection, Scopus, the Cochrane Central Register of Controlled Trials, and Google Scholar from database inception to 20 May 2026. These databases were selected due to their extensive coverage of biomedical, clinical, and pharmacological research. In addition, lists of references of relevant review articles and primary studies were manually screened to identify further eligible publications not retrieved through database searching. A broad initial search strategy was used to maximize sensitivity and ensure comprehensive coverage of the literature. The following keywords and Medical Subject Headings (MeSH) terms were combined using Boolean operators (AND/OR): “obesity” OR “overweight” OR “weight management” OR “weight loss” AND “female reproductive health” OR “fertility” OR “infertility” OR “menstrual irregularities” OR “polycystic ovary syndrome” OR “PCOS” AND “anti-obesity drugs” OR “pharmacotherapy” OR “weight loss medications” OR “GLP-1 receptor agonists” OR “orlistat” OR “liraglutide” OR “semaglutide” OR “orlistat” OR “phentermine topiramate” OR “naltrexone bupropion” OR “metformin”, OR “exenetide” OR “tirzepatide”. Additional terms such as “hypothalamic–pituitary–gonadal axis,” “ovulation,” “endometrial receptivity,” and “assisted reproductive technology (ART)” were included to capture mechanistic and clinical reproductive outcomes. The search was limited to studies published in the English language to ensure consistency in interpretation and data extraction. No restriction was placed on publication date in order to include both foundational and contemporary evidence. However, greater emphasis was placed on studies published within the last two decades, reflecting the rapid development of anti-obesity pharmacotherapies. Eligible studies included randomized controlled trials (RCTs), prospective and retrospective cohort studies, observational studies, systematic and narrative reviews, and meta-analyses. Preclinical animal studies and mechanistic in vitro research were also included when they provided relevant insights into reproductive endocrinology or drug mechanisms of action, whereas case reports were excluded. Study selection followed a two-stage screening process. Initially, titles and abstracts were screened for relevance to obesity, pharmacological weight-loss interventions, and female reproductive outcomes. Full-text articles were then assessed for eligibility based on predefined inclusion criteria. Studies were included if they evaluated (i) the effect of obesity on female reproductive function, (ii) the impact of weight loss on fertility or reproductive hormones, or (iii) the effects of FDA-approved and off-label anti-obesity medications on metabolic and/or reproductive endpoints. Data extraction focused on study design, population characteristics [particularly BMI (body mass index) and reproductive status], type of pharmacological intervention, duration of treatment, and primary outcomes, including weight loss, ovulatory function, hormonal changes [such as insulin, SHBG, LH/FSH (Luteinizing Hormone/Follicle-Stimulating Hormone), androgens], menstrual regularity, pregnancy rates, and outcomes in assisted reproductive technologies such as in vitro fertilization (IVF). Finally, the identified evidence was synthesized narratively due to heterogeneity in study design, interventions, and outcome measures. This approach allowed for an integrated interpretation of mechanistic and clinical findings, highlighting both established effects and current gaps in knowledge regarding the reproductive impact of anti-obesity pharmacotherapies.

The literature included in this review was identified through a structured search process and selected using predefined eligibility criteria to ensure a transparent and consistent approach. Nevertheless, the review was not designed or conducted as a systematic review, as it did not incorporate key methodological components such as protocol registration, independent study selection by multiple reviewers, or a formal assessment of the risk of bias. For this reason, the findings are presented as a narrative synthesis of the available evidence.

## 3. Mechanistic Insights into Obesity-Related Female Reproductive Dysfunction

Obesity in women is associated with dysfunction of the hypothalamic–pituitary–gonadal (HPG) axis [20]. In obese women, the reproductive disorders include PCOS, irregular menstrual periods, endometriosis, infertility and pregnancy complications [21]. Dysregulation of the hormone leptin, which is secreted mainly by white adipose tissue, plays a crucial role in several female reproductive disorders [20]. Also, adipose tissue is a major site for the metabolism of the sex steroid hormones, androgens and estrogens, and therefore, through this pathway, affects the reproductive axis in obesity [20]. Epidemiological studies indicate that serum levels of SHBG (the main transporter of sex hormones) are decreased in obesity [20,22]. These low levels of SHBG in obese patients result from insulin resistance (IR) and compensatory hyperinsulinemia, which is apparently more prevalent in central obesity [20]. Moreover, obesity is a risk factor for anxiety and depression, which may, in females, manifest with sexual avoidance, low sexual desire and interest, lack of subjective arousal and sexual pleasure and difficulties in sexual performance [20,23]. It has become evident that obesity contributes to menstrual disturbances due to HPG dysfunction from menarche to menopause. Body weight is strongly related to the onset of menarche and maintenance of a normal menstrual cycle [20,24]. Several studies support a trend of earlier pubertal onset in overweight girls compared to those with normal weight [25]. Both overall and central obesity are associated with menstrual disturbances extending from dysmenorrhea and menstrual irregularities to anovulation and amenorrhea, therefore increasing the risk of sub-fecundity and infertility [20,26]. The perturbation of the hypothalamic–pituitary–ovarian axis and neuroendocrine system in obese women causes aberrant gonadotropin secretion, reduced ovarian folliculogenesis and lower progesterone levels during the luteal phase of the female menstrual cycle, increasing the risk for sub-fecundity and infertility [27,28]. The risk of anovulatory infertility is more than doubled in women with obesity compared to those with normal BMI [27]. Interestingly, body fat distribution is an important risk factor for the presentation of anovulatory cycles. Central obesity leads commonly to anovulation compared to obesity with the same BMI but less abdominal fat accumulation [27]. Adipose tissue itself acts as an endocrine organ, responsible for the synthesis and secretion of several hormones, such as leptin [28]. Leptin is a putative signal that links metabolic status with reproductive function [29]. Leptin augments secretion of gonadotropin hormones, which are needed for initiation and maintenance of normal menstrual cycles, by acting centrally at the hypothalamus to stimulate release of gonadotropin-releasing hormone (GnRH) [20,24]. Indeed, in female patients, gaining weight stimulates production and release of LH and FSH into the peripheral circulation, leading to resumption of menstruation [20,30]. However, leptin levels increase with the expansion of adipose tissue in obesity [31]. Leptin receptor is expressed in the human ovary and, in particular, in pre-ovulatory follicles, ovarian theca cells, ovarian granulosa cells and oocytes. In vitro studies have shown that leptin affects steroidogenesis in granulosa cells and decreases both estrogen and progesterone production in a dose-dependent manner [31]. The high leptin levels observed in obese patients act in the ovaries and suppress estradiol production. Also, the high leptin levels affect the development of dominant follicles, decreasing the aromatization capacity of granulosa cells and predisposing to anovulation [32]. Moreover, the increased levels of leptin because of obesity may deregulate the hypothalamic–pituitary–gonadal system in women, leading to reproductive dysfunction, including infertility [31]. Anti-Mullerian hormone (AMH) levels decline as BMI increases and AMH is significantly lower in individuals with obesity compared to those with normal weight [33]. The lower systemic levels of adiponectin observed in obesity may be related with the decreased anti-Mullerian hormone (AMH) production by the granulosa cells of the ovarian follicles, which reflects a reduced ovarian reserve in follicles across the reproductive life of obese women [34,35]. The hormonal levels of insulin, estrogens and androgens are changed in obese women [36]. In particular, central obesity in women is linked to elevated serum insulin levels due to insulin resistance, which decreases sex hormone-binding globulin (SHBG) synthesis [36]. Women with obesity have altered androgen and estrogen levels due to increased exogonadal aromatization of androgens to estrogens in adipose tissue depots [20,37]. Obesity in women with polycystic ovarian syndrome (PCOS) enhances insulin resistance and worsens the clinical manifestations of PCOS [5,38]. The syndrome is a clinical heterogeneous disorder characterized by enlarged ovaries with multiple follicles, anovulation and hyperandrogenism [39]. Obese polycystic ovarian syndrome (PCOS) patients, compared with normal-weight PCOS patients, have lower serum SHBG levels, higher free testosterone, free androgen index (FAI), insulin resistance homeostasis assessment (HOMA-IR), fasting insulin (FINS) and fasting blood glucose levels [40,41]. In addition, it has been found that severe hyperinsulinemia promotes hyperandrogenism independently of gonadotropins, which results in reproductive dysfunction in women [27,42]. Moreover, obesity increases the conversion of androgens to estrogens in peripheral adipocytes [27].

**Figure 1:**
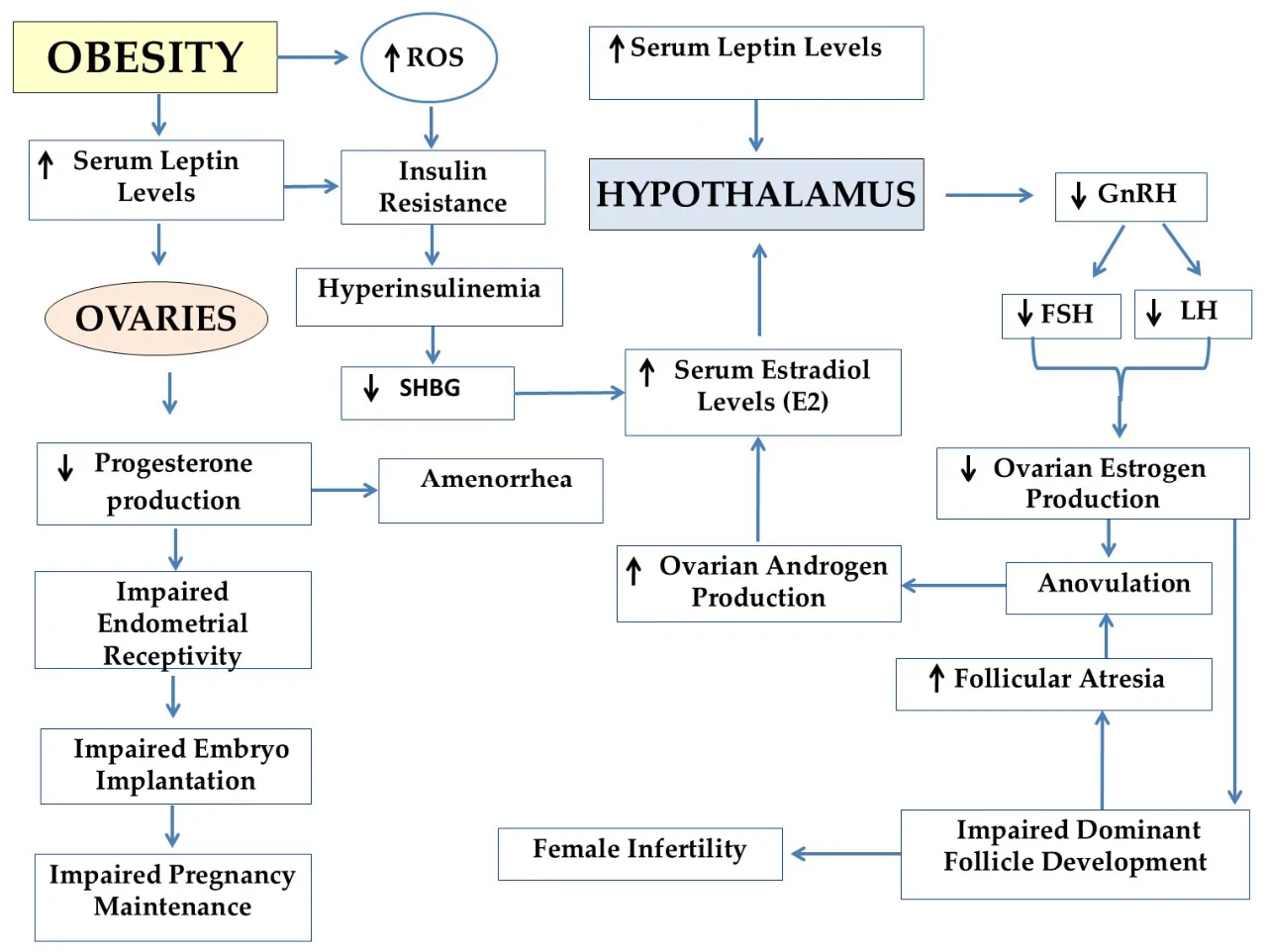
Schematic illustration of the mechanisms by which obesity is related to female infertility. Obesity disrupts female reproductive function through hormonal, metabolic, and inflammatory mechanisms. Hyperleptinemia, insulin resistance (IR), and hyperinsulinemia impair hypothalamic–pituitary–ovarian (HPO) axis function, decrease SHBG levels, alter estrogen and androgen homeostasis, and promote follicular atresia, anovulation, poor endometrial receptivity, impaired embryo implantation, and infertility.

Women with obesity have lower fertility rates and more miscarriages and they take a longer time to conceive compared to normal-weight women [35,43]. Also, obese women with normal menstrual cycles suffer from sub-fecundity [26,44]. Studies have shown that for every unit increase in BMI exceeding 29 Kg/m^2^, the chance of spontaneous conception decreases by 5% [45,46]. The underlying mechanisms of infertility in obese individuals are multifactorial, involving insulin resistance, alterations in ovarian steroids and gonadotropins secretion, anovulation, negative effects on oocyte development, embryo quality and endometrial receptivity [28]. Figure 1 illustrates the pathophysiological mechanisms by which obesity may disrupt normal reproductive function in women with obesity, leading to infertility.

Obesity causes early onset of puberty. Normally in females after puberty, leptin increases the secretion of the gonadotropin hormone FSH, which is required to initiate and maintain normal menstrual cycles by acting centrally at the hypothalamus to regulate GnRH neuronal activity and by acting directly on the gonadotrophic cells from the anterior lobe of the pituitary gland. Increased leptin levels in obesity are responsible for central (hypothalamic and pituitary) leptin resistance. Thus, leptin cannot stimulate the pulsatile secretion of GnRH from the hypothalamus as well as the gonadotropins FSH and LH from the anterior pituitary. In addition, the high levels of leptin observed in obese patients act directly on the ovaries and reduce the development of the dominant follicle in the ovaries, thereby reducing the aromatizing capacity of granulosa cells of the dominant follicle and thus predisposing it to atresia and anovulation. In obesity, the elevated serum insulin levels due to insulin resistance reduce hepatic SHBG synthesis and result in increased circulating free estrogen levels. In women with obesity and anovulatory cycles, there are increased levels of androgens, as, for example, occurs in women with PCOS. These elevated androgens aromatize to estrogens in extragonadal sites (adipose tissue depots). The elevated estrogen levels with negative feedback mechanisms affect the hypothalamus and the pituitary gland and suppress the function of the hypothalamus–pituitary–ovary axis. The final result is the reduction in ovarian estrogen production, anovulatory cycles, amenorrhea and infertility. The anovulatory cycles result in an absence of corpus luteum formation in the ovaries. Therefore, progesterone is not produced by the corpus luteum, the endometrium shows poor receptivity for pregnancy, and therefore the chance of fetal implantation is limited.

Once conception is achieved in obese patients, there are maternal and perinatal complications [28]. Maternal obesity affects fetal growth, including both macrosomia and fetal growth restriction [47]. Also, obesity increases the risk of stillbirth [47]. Obesity during pregnancy predisposes to preterm deliveries, which can lead to significant complications of the neonate, including respiratory distress syndrome, admission to neonatal intensive care unit (NICU), intra-ventricular hemorrhage, necrotizing enterocolitis and mortality [48]. Women with obesity are at increased risk for preeclampsia, gestational diabetes, cesarean delivery, venous thromboembolism, anesthetic complications and wound infections [49,50]. Obesity has a negative impact on the success of IVF treatment [28]. Obese anovulatory women undergoing IVF respond poorly to hormonal therapy to stimulate ovarian follicle development with a need for prolonged duration of ovulation induction and higher gonadotropin doses for ovarian stimulation. Moreover, the number of large and medium-size follicles, which are developed after ovulation induction, and the final number of oocytes, which are also retrieved, is lower in obese patients compared to those with normal weight [51,52]. In addition, the rates of IVF cancelation cycles are increased in obese compared to normal weight patients [26,27,53]. Finally, women with obesity and IVF treatment have poor-quality embryos, lower blastocyst formation rates, lower live birth rates and more miscarriages than women with a normal BMI [32,51,52]. Performing oocyte retrieval and embryo transfer may pose more technical difficulties in obese women who undergo IVF treatment than in women with normal BMI, leading to a higher incidence of infection and bleeding during the oocyte retrieval [54]. Also, there is an increased anesthesia risk for the obese patient during the egg retrieval [53]. In obese women, lipotoxicity causes macrophage infiltration and upregulation of pro-inflammatory cytokines in ovarian cells, which may contribute to the reduced pregnancy rates observed in response to obesity [52,55,56]. Moreover, C-reactive protein (CRP) has been found to be strongly associated with obesity [57], whilst the high serum CRP levels on the day of oocyte retrieval in assisted reproduction techniques (ART) seem to have negative effects on embryo quality [52,58]. Obese women produce high levels of triglycerides and non-esterified free fatty acids in their ovarian follicular fluid, which are associated with high levels of reactive oxygen species in follicular fluid, aggravation of oxidative stress (OS) and negative effects on the success of their assisted reproduction techniques (ART) [46,59,60]. Moreover, the increased follicular fluid triglyceride levels are associated with impaired mitochondrial metabolism in oocytes and subsequent failure of oocytes to cleave [61]. Regarding clinical pregnancy rates in women with obesity undergoing gonadotropin intrauterine insemination (IUI), the findings are mixed. Some studies have reported no difference in the clinical pregnancy rates after IUI [62] compared to non-obese controls, while several other studies reported a paradoxical increase [63]. However, this increase in clinical pregnancy rates after IUI in obese patients may be explained by the fact that obesity is associated with decreased sexual desire, erectile dysfunction and decreased frequency of sexual intercourse [27]. Studies have emphasized that obesity has a negative impact on endometrial receptivity through delaying the window of implantation [64]. Human endometrial stromal cells in obese women have alterations in endometrial gene expression, leading to reduced ability to undergo normal decidualization, which may inhibit endometrial receptivity [52]. As it is known, endometrial receptivity is the process undertaken by the uterine endometrium to prepare embryo implantation [65]. Therefore, the negative impact of obesity on endometrial receptivity is responsible for the failure of embryo implantation and infertility [66].

## 4. Impact of FDA-Approved Long-Term Anti-Obesity Medications on Female Reproductive Health

### 4.1. Orlistat

Orlistat was approved by the FDA in 1999 for the long-term management of obesity. It is a synthetic derivative of lipstatin, a molecule produced by the bacterium *Streptomyces toxytricini*, and it acts by inhibiting the pancreatic and gastric lipases, enzymes responsible for the breakdown of triglycerides in the intestine. As a result, the absorption of approximately one-third of dietary fat is reduced, thereby decreasing the overall caloric intake [12,14]. However, it does not affect the sensation of hunger [67]. It is administered in doses of 60 mg or 120 mg three times daily, together with the main meals that contain fat, while patients are advised to keep fat intake below 30% per meal [67]. Despite its efficacy, it is not widely used for the treatment of obesity, mainly due to its unpleasant adverse effects, which are directly related to its mechanism of action. These adverse effects are primarily gastrointestinal disturbances, such as abdominal pain, flatulence, urgent bowel movements, frequent stools, fatty diarrhea, and fecal incontinence, as well as potential deficiencies in fat-soluble vitamins (A, D, E, and K) due to reduced fat absorption [14]. For this reason, patients taking it may require supplementation with these vitamins [67]. Orlistat is contraindicated in patients with pre-existing malabsorption syndromes, such as ulcerative colitis and Crohn’s disease [68]. It is also not recommended for use in cases of cholestasis [68]. Despite these contraindications, it may provide benefits in patients with dyslipidemia [67]. Studies in animal models, such as rats and rabbits, did not show evidence of embryotoxicity or teratogenicity, even when supratherapeutic doses of orlistat were administered [67]. In the XENDOS study (XENical in the prevention of Diabetes in Obese Subjects), the largest randomized controlled trial (RCT) evaluating the efficacy of orlistat, the administration of 120 mg three times daily was compared with placebo, both in combination with lifestyle interventions (diet, physical activity, and behavioral modification) in 3305 individuals with a BMI ≥ 30 kg/m^2^. Of these participants, 79% had normal glucose tolerance and 21% had impaired glucose tolerance (IGT). The primary endpoints of the study were time to onset of type 2 diabetes mellitus and changes in body weight. The results showed a mean weight loss of 2.4% after 4 years in the orlistat group, a finding that may have limited clinical significance [69]. Despite the relatively modest weight loss, significant metabolic benefits were observed, including a reduced risk of developing type 2 diabetes mellitus, as well as improvements in blood pressure, insulin sensitivity, and lipid profile [69].

Few studies have examined the effect of orlistat on fertility in overweight and obese infertile women. In the study by Vosnakis I. et al. (2013) [70], the administration of orlistat in women with PCOS showed a beneficial effect both in reducing body weight and in improving the reproductive hormonal profile. The study included 61 obese women with PCOS and 20 overweight and obese control subjects [70]. Participants followed a hypocaloric diet and an exercise program and received orlistat 120 mg three times daily for 24 weeks. The results showed that women with PCOS experienced a significant increase in LH and SHBG levels, as well as a reduction in serum testosterone. An increase in serum AMH levels was also observed in women with PCOS, suggesting a possible improvement in ovarian function and ovarian reserve. At the same time, the combination of pharmacological treatment and lifestyle modification led to significant weight loss and a reduction in BMI in the PCOS group [70]. In the RCT by Kumar et al. (2014) [71], involving 90 women with polycystic ovary syndrome (PCOS), the effect of orlistat on pregnancy rates was evaluated in comparison with metformin and lifestyle intervention. Participants were equally allocated into three groups: orlistat 120 mg twice daily, metformin with gradual dose escalation up to a maximum of 500 mg three times daily, and a control group that received only behavioral intervention, diet, and physical exercise for duration of 3 months [71]. The results showed a comparable reduction in body weight between the two treatment groups (7.81 ± 0.66 kg in the orlistat group versus 7.78 ± 0.57 kg in the metformin group), as well as a similar decrease in BMI (8.12 ± 6.71 kg/m^2^ versus 8.40 ± 0.65 kg/m^2^, respectively). Ovulation rates were 33.3% in the orlistat group and 23.3% in the metformin group, with no statistically significant difference. Likewise, pregnancy rates were 40% in the orlistat group, 16.7% in the metformin group, and 3.3% in the control group, suggesting a favorable effect of orlistat on reproductive outcomes [71]. In the study by Tong et al. (2022) [72], 29 patients receiving orlistat completed 37 embryo transfer cycles, while 29 individuals in the control group completed 38 cycles. The clinical pregnancy rate was significantly higher in the orlistat group (59.46%) compared with the control group (39.47%). However, no statistically significant difference was observed in the live birth rate between the two groups [72]. In the prospective study by Al-Qahwajy et al., 120 overweight and obese infertile women aged 21–35 years with a BMI > 25 kg/m^2^ were included. Participants were divided into two groups: the first group received orlistat 120 mg twice daily for 6 months, while the second group underwent lifestyle interventions, including diet, physical exercise, and behavioral modification. The results showed that the orlistat group had significantly higher pregnancy rates compared with the control group [73].

**Figure 2:**
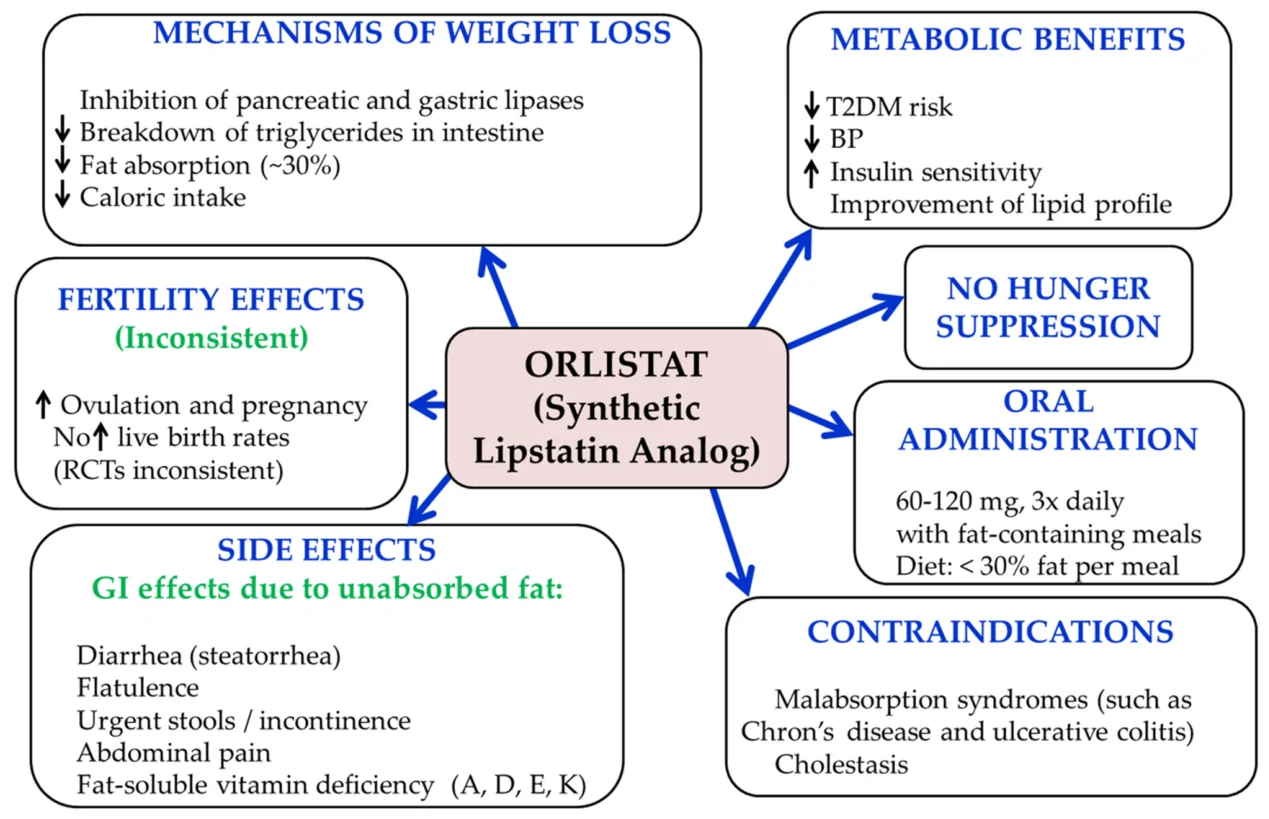
Mechanisms of action and metabolic and reproductive effects of orlistat in obese women.

Despite the encouraging findings of the above studies, the results are not entirely consistent. Wang et al. (2021), in a double-blind RCT, investigated whether orlistat-induced weight loss prior to IVF could improve live birth rates (LBRs) in overweight or obese women following fresh embryo transfer [74]. A total of 877 infertile obese women participated in the study and were randomized to receive either orlistat or placebo for a period of 4 to 12 weeks. The results showed that there were no statistically significant differences between the two groups in live birth rates, nor in conception or clinical pregnancy rates. However, a greater reduction in body weight was observed in the orlistat group (2.49 kg) compared with the placebo group (1.22 kg), a difference that was statistically significant [74]. The FIT-PLESE study, a prospective randomized controlled trial conducted by Legro R.S. et al. (2022) [75], investigated the effect of preconception interventions in 379 women with obesity and unexplained infertility. Participants were assigned either to an intensive intervention group or to a standard-care group. The intensive group received increased physical activity combined with weight loss strategies using meal replacements and pharmacological treatment with orlistat, while the standard group followed increased physical activity without a specific focus on weight reduction. Although the intensive intervention group achieved significantly greater weight loss (−6.6 ± 5.4%) compared with the standard group (−0.3 ± 3.2%), no significant differences were observed in live birth rates. These findings suggest that targeted weight loss, even when achieved through combined interventions including orlistat, does not necessarily translate into improved reproductive outcomes in this population of women [75]. In addition, a non-statistically significant trend toward higher miscarriage rates was observed in the orlistat group, mainly after implantation (15.9%), compared with the standard group (5.1%). This finding was attributed by the authors to a possible reduced absorption of long-chain polyunsaturated fatty acids [75]. At the same time, the potential development of vitamin D deficiency due to the action of orlistat may have a negative impact, given that adequate vitamin D levels have been associated with higher conception rates, improved implantation—particularly in in vitro fertilization (IVF) cycles—and a reduced risk of pregnancy complications for both the mother and the fetus, also affecting the antioxidant status of the follicles and potentially the embryo [76]. Due to the conflicting evidence regarding the effect of orlistat on fertility, further research is necessary to clarify its role in female reproductive function. Figure 2 illustrates the mechanisms of action, dosage, metabolic and reproductive effects, side effects, and contraindications of orlistat in women with obesity.

### 4.2. Liraglutide

Liraglutide is an acylated analog of GLP-1, consisting of 31 amino acids, with 97% homology to human GLP-1 [10,77]. Its composition involves the addition of a fatty acid side chain to the ε-amino group of lysine at position 26, as well as the replacement of lysine with arginine at position 34 [10,77]. These structural modifications enhance its binding to albumin, extending its half-life beyond 13 h and allowing it to be administered by subcutaneous injection once daily [10]. Liraglutide was approved for the treatment of type 2 diabetes mellitus in Europe in 2009 and in the U.S. in 2010 by the FDA, at daily doses of 0.6, 1.2 or 1.8 mg [77]. Later, in 2014, it was also approved for the treatment of obesity, with a recommended subcutaneous maintenance dose of 3.0 mg daily [77]. Its use is not indicated in individuals with a personal or family history of medullary thyroid carcinoma (MTC) or multiple endocrine neoplasia type 2 (MEN2) syndrome [68]. Liraglutide promotes weight loss through a combination of central and peripheral mechanisms [78]. It enhances satiety by directly activating GLP-1 receptors in the brainstem (area postrema and nucleus tractus solitarius) and hypothalamus (arcuate nucleus/ARC, paraventricular nucleus/PVN, and dorsomedial hypothalamus). In addition, it indirectly inhibits the activity of orexigenic neuropeptide Y (NPY)/agouti-relate peptide (AgRP) neurons while stimulating anorexigenic pro-opiomelanocortin (POMC)/cocaine- and amphetamine-regulated transcript (CART) neurons in the arcuate nucleus, thereby contributing to the regulation of appetite and energy balance [10,12]. During long-term weight-loss maintenance, liraglutide supports sustained outcomes by helping to maintain leptin levels and enhance peptide YY (PYY) secretion, thereby promoting adaptive metabolic adjustments that favor energy conservation [10]. Its peripheral actions include delayed gastric emptying and activation of the ileal brake, both of which contribute to enhanced satiety [78]. In addition, it increases resting energy expenditure, enhances glucose-dependent insulin secretion, promotes pancreatic β-cell proliferation (as shown in experimental models), and suppresses glucagon secretion [78]. The SCALE clinical trial, as described by Pi-Sunyer X. et al. (2015) [79], is one of the largest and most significant randomized controlled trials investigating the effectiveness of liraglutide in the management of obesity. The study included a total of 3731 adult patients with obesity (BMI > 30 kg/m^2^ or >27 kg/m^2^ in the presence of dyslipidemia or hypertension, whether treated or untreated) without type 2 diabetes. Of these participants, 78% were women, with a mean age of approximately 45 years. Participants were randomized to receive either subcutaneous injections of liraglutide at a dose of 3.0 mg daily or a placebo, in combination with lifestyle interventions such as a hypocaloric diet and increased physical activity. The duration of the intervention was 56 weeks, allowing for the assessment of both short- and medium-term effects of the treatment on body weight. The results demonstrated that liraglutide was significantly more effective compared to placebo in achieving weight loss. Specifically, 63.2% of participants receiving liraglutide achieved a weight loss of at least 5% of their initial body weight, while 33.1% achieved a weight loss of ≥10%. Additionally, the treatment was associated with improvements in cardiometabolic parameters, including glucose and lipid levels, as well as a reduction in waist circumference, indicating an overall improvement in the patients’ metabolic profile. Overall, the SCALE study demonstrated that liraglutide at a dose of 3.0 mg is an effective and clinically meaningful therapeutic option for the management of obesity, particularly when combined with lifestyle modifications [79].

**Figure 3:**
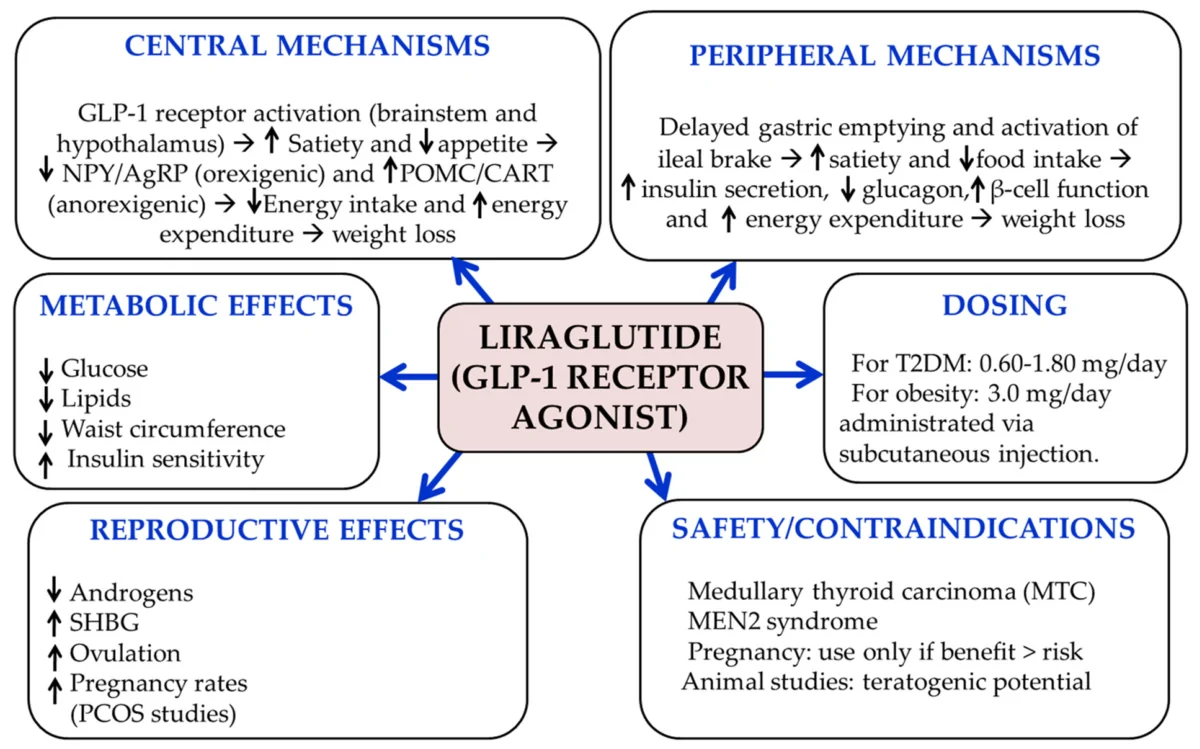
Mechanisms of action and metabolic and reproductive effects of liraglutide in obese women.

Liraglutide, in addition to regulating appetite and weight, exerts protective effects on many tissues, including those of the reproductive system, through complex and interconnected signaling pathways that affect inflammation, oxidative stress, apoptosis, and metabolism [10]. Weight loss by liraglutide reduces inflammation, oxidative stress and hyperinsulinemia, improving insulin sensitivity and ultimately contributing to the reduction in androgen production, particularly in conditions such as obesity and polycystic ovary syndrome (PCOS) [10]. There is limited direct clinical data regarding exposure to liraglutide during pregnancy in humans. However, preclinical animal studies have demonstrated potential teratogenic effects. Specifically, in the offspring of pregnant rats administered liraglutide at doses ≥ 0.8 times the systemic exposure achieved with a 3 mg once-daily subcutaneous dose in humans, vascular, renal, skeletal, and oropharyngeal malformations have been observed [67]. According to the FDA, liraglutide should be used during pregnancy only when the expected therapeutic benefit clearly outweighs the potential risk to the fetus. Although there are no specific official recommendations regarding the timing of discontinuation before pregnancy, liraglutide has a relatively shorter half-life compared to semaglutide. For semaglutide, discontinuation at least two months prior to attempting conception is recommended, which is often used as an indirect reference point when planning pre-pregnancy discontinuation of similar medications [67]. Liraglutide has shown promising results in improving both metabolic and reproductive parameters in non-diabetic women with obesity and polycystic ovary syndrome [10]. In particular, the double-blind, randomized controlled trial conducted by Nylander M. et al. (2017) [80] evaluated the effects of liraglutide (1.8 mg once daily, subcutaneously) compared with placebo over a 26-week period in 72 overweight women diagnosed with PCOS. The intervention group achieved a mean body weight loss of 5.2 kg over the six-month follow-up, along with increased menstrual regularity and improved ovarian morphology, including a significant reduction in ovarian volume of 1.6 mL compared with the placebo group. In addition, increases in serum SHBG concentrations and decreases in free testosterone levels were observed, suggesting the potential efficacy of liraglutide in the management of PCOS [80]. In addition, in the prospective, open-label RCT by Salamun V. et al. (2018) [81], 28 infertile obese women with PCOS were studied to evaluate whether treatment with metformin alone or in combination with liraglutide could improve pregnancy rates in this population undergoing ART. Participants were allocated into two treatment groups: the first group received metformin (MET) at a dose of 1000 mg twice daily, while the second group received metformin at the same dosage combined with low-dose liraglutide (1.2 mg once daily, subcutaneous administration). The pharmacological treatment lasted 12 weeks, aiming to improve body weight and metabolic parameters prior to attempts at conception. This was followed by a 4-week washout period, after which an ovarian stimulation protocol was initiated as part of ART, in order to assess the effect of the interventions on reproductive outcomes. The results showed that both therapeutic approaches led to significant weight loss. Specifically, patients in the MET group lost an average of 7.0 ± 6.0 kg, while those in the combination therapy (COMBI) group lost 7.5 ± 3.9 kg, with no statistically significant difference between the two groups. However, regarding reproductive outcomes, combination therapy demonstrated clear superiority: the pregnancy rate per embryo transfer was 85.7% in the COMBI group compared with 28.6% in the MET group. In addition, the cumulative pregnancy rate over 12 months was higher in the combination group (69.2%) compared with the metformin-only group (35.7%). Overall, these findings suggest that despite comparable weight loss, the addition of liraglutide to metformin may offer a significant advantage in improving pregnancy outcomes in obese women with PCOS [81]. In sum, liraglutide may improve female reproductive outcomes, particularly in obese women with PCOS, by enhancing insulin sensitivity, reducing weight, and improving hormonal balance and ovarian function. However, evidence remains limited and reproductive safety is uncertain. Future large, randomized trials are needed to clarify its effectiveness, optimal use, and pregnancy safety. Figure 3 illustrates the central and peripheral mechanisms of action, the dosage, metabolic and reproductive effects, and the contraindications of liraglutide in women with obesity.

### 4.3. Semaglutide

Semaglutide is a synthetic analog of the hormone GLP-1 (glucagon-like peptide-1), sharing approximately 94% sequence homology with the native human peptide [12,82]. Despite this high degree of similarity, it has been structurally modified to enhance its stability and significantly extend its pharmacokinetic profile, with a reported elimination half-life ranging from approximately 155 to 184 h [12]. Specifically, targeted modifications have been introduced into the peptide sequence; at position 8, the amino acid alanine has been replaced by 2-aminoisobutyric acid (Aib), a substitution that confers resistance to enzymatic degradation by dipeptidyl peptidase-4 (DPP-4), the enzyme responsible for the rapid inactivation of endogenous GLP-1 [77,83,84]. Additionally, at position 34, lysine has been replaced by arginine, enabling further chemical modification of the molecule. One key modification is the attachment of a long-chain fatty acid via the lysine residue at position 26. This acylation promotes reversible binding of semaglutide to circulating albumin, which reduces renal clearance and protects the molecule from premature degradation. As a result, semaglutide exhibits a markedly prolonged half-life, allowing for sustained pharmacological activity and enabling once-weekly administration [77,82,84]. Functionally, semaglutide mimics the physiological actions of endogenous GLP-1 by enhancing glucose-dependent insulin secretion from the pancreas and suppressing glucagon release. In addition, it delays gastric emptying, thereby attenuating postprandial glucose excursions [84,85]. Moreover, semaglutide exerts effects on the central nervous system, particularly on appetite-regulating pathways within the hypothalamus, brainstem and septal nuclei, resulting in reduced hunger and decreased caloric intake [12,85]. Collectively, these mechanisms contribute to improved glycemic control as well as significant reductions in body weight [84,85]. Semaglutide was first approved in the United States in 2017 for the treatment of adults with type 2 diabetes mellitus [86]. Subsequently, higher-dose formulations were approved for long-term weight management in adults with obesity or overweight with associated comorbidities [83,87]. An effective dose of semaglutide for weight reduction is 2.4 mg administered once weekly via subcutaneous injection. Treatment is initiated at a dose of 0.25 mg per week for the first four weeks, followed by a gradual dose escalation at four-week intervals until the target dose of 2.4 mg weekly is achieved [12]. The systematic review and meta-analysis by Gao et al. (2022) [88] evaluated the efficacy and safety of semaglutide for weight loss in obese or overweight adults without diabetes. Based on data from randomized controlled trials, the analysis demonstrated that semaglutide significantly reduced body weight compared with placebo, with mean reductions of approximately 10% or more. In addition, treatment with semaglutide was associated with improvements in secondary anthropometric outcomes, including reductions in BMI and waist circumference [88]. Injectable semaglutide (2.4 mg once weekly) has been associated with a mean body weight reduction of approximately 14.9% over a treatment period of around 68 weeks, compared to 15.1% with oral semaglutide (50 mg daily) in the OASIS 1 study [89,90]. Semaglutide carries a “boxed warning” regarding the potential risk of thyroid C-cell tumors, based on findings from animal studies [67]. For this reason, its use is not recommended in patients with a personal or family history of medullary thyroid carcinoma (MTC) or in patients with multiple endocrine neoplasia type 2 (MEN2) [67]. There is a relative contraindication, or at least increased caution, regarding the use of semaglutide in patients with a history of acute pancreatitis, as cases of worsening or recurrence of the condition have been reported. The most common adverse effects of the drug concern the gastrointestinal system, including nausea, vomiting, diarrhea, and abdominal pain, particularly during treatment initiation or dose escalation [67]. Available studies on the effects of semaglutide on reproductive health are currently limited, especially regarding clinical data [77]. Most evidence derives from preclinical animal studies, in which adverse outcomes have been observed, including embryotoxicity, delayed fetal development, and an increased risk of congenital abnormalities, especially at doses associated with significant maternal weight loss [91]. Due to these findings, and the lack of sufficient and well-established safety data in humans, the use of semaglutide is not recommended during pregnancy [91]. Based on the available data, including the recent study by Diab et al. (2024) [92], the use of subcutaneous semaglutide during breastfeeding appears, to date, to be associated with a low likelihood of infant exposure through breast milk. The findings suggest that the transfer of the active substance into human milk is extremely limited and that its oral absorption by the infant is also expected to be minimal, due to its peptide structure and potential degradation within the gastrointestinal tract [92]. Despite these encouraging findings, the overall evidence remains limited and is based on a small number of cases and studies, which does not allow for definitive conclusions regarding the safety of long-term use during lactation. Therefore, although current data do not indicate a significant risk to the breastfed infant, the use of semaglutide during breastfeeding should be considered on an individual basis, taking into account the maternal benefit and the limited clinical experience available [92]. Weight loss induced by semaglutide may have several beneficial metabolic and reproductive effects. These include improvements in insulin sensitivity, reductions in systemic inflammatory markers, and partial restoration of normal gonadotropin secretion. Collectively, these changes may enhance ovarian responsiveness and improve the likelihood of regular ovulation, which is a critical prerequisite for fertility and successful conception. However, these potential benefits should be interpreted with caution, as reproductive outcomes remain an emerging area of investigation [11]. The study by Carmina and Longo (2023) [93], involving obese women with PCOS, reported that treatment with semaglutide (0.5 mg subcutaneously once a week) was associated with significant weight reduction and, in a substantial proportion of responders, normalization of menstrual cycles. These findings suggest that, in addition to its metabolic benefits, semaglutide may also contribute to improvement in reproductive function. Overall, the observed outcomes indicate that the weight loss and metabolic effects of semaglutide may translate into clinically meaningful enhancements in menstrual regularity, particularly in women with PCOS [93]. A prospective, randomized, controlled clinical trial by Chen H et al. (2025) [94] evaluated the effects of combined therapy with metformin (1000 mg twice daily) and semaglutide (1 mg once weekly) in overweight and obese women with PCOS, compared with metformin monotherapy over 16 weeks. The study assessed changes in body weight, metabolic parameters, and reproductive outcomes. The results showed that combination therapy led to significantly greater reduction in body weight and improvement in metabolic indices, including insulin resistance and glycemic control, compared with baseline and metformin alone. Additionally, the combination was associated with improved reproductive outcomes, such as enhanced menstrual regularity and markers of ovulatory function. Notably, natural pregnancy rates were higher in the combination group (35%) than in the metformin-only group (15%). Overall, these findings suggest that semaglutide, particularly when combined with metformin, may offer clinically meaningful benefits in addressing both metabolic and reproductive dysfunction in women with PCOS [94]. Therefore, semaglutide may exert beneficial indirect effects on female reproductive health, primarily through significant weight loss and improvements in metabolic function. These changes are particularly relevant in conditions such as PCOS, where the metabolic dysfunction contributes to anovulation and subfertility. It seems that semaglutide, especially when combined with metformin, can enhance menstrual regularity and may improve natural conception rates in selected populations. However, despite these promising findings, the use of semaglutide in women actively attempting conception or during pregnancy remains contraindicated due to insufficient safety data and potential risks to fetal development. Current clinical guidance therefore supports its use only as part of a supervised preconception weight management strategy, with discontinuation being recommended prior to conception attempts. However, recent findings indicate that these agonists facilitate follicular progression and improve oocyte competence by combating metabolic and oxidative stress inside the follicular compartment [8].

**Figure 4:**
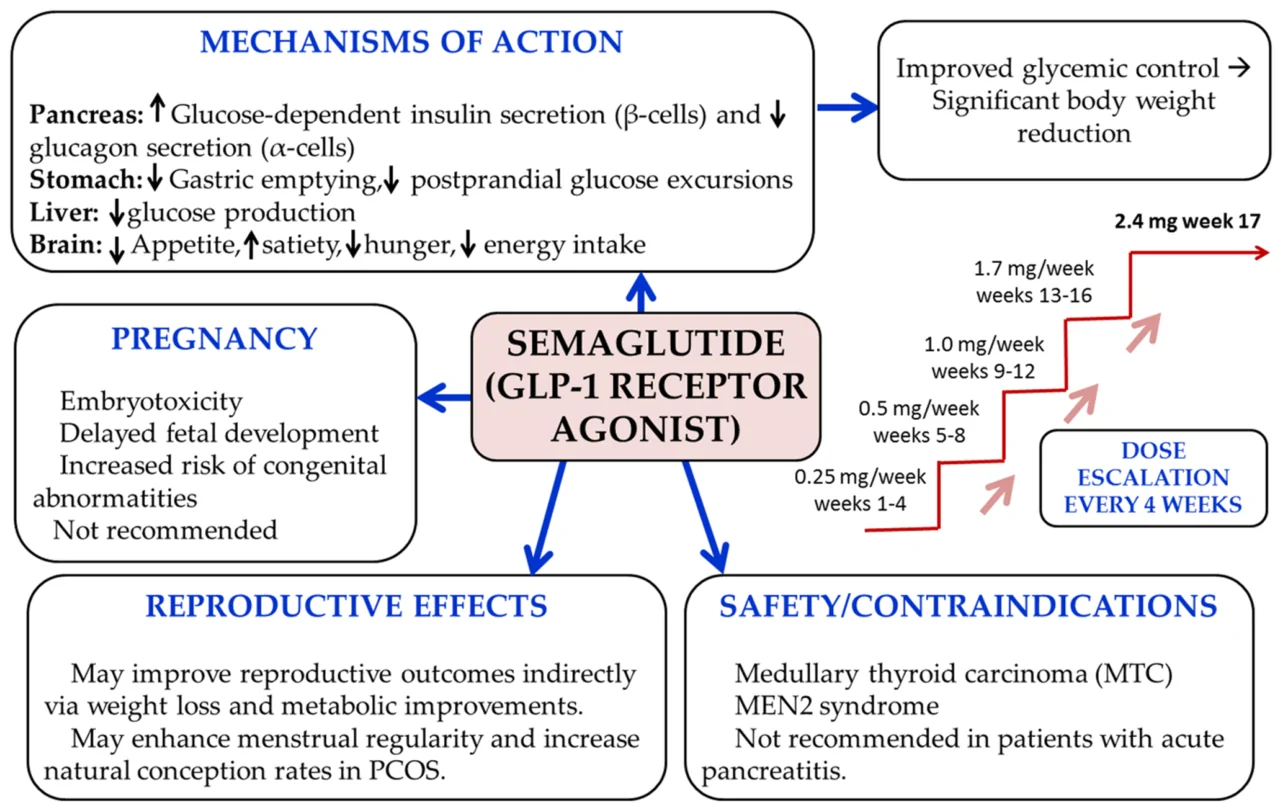
Mechanisms of action, dose escalation, contraindications and reproductive effects of semaglutide in obese women.

Importantly, the existing evidence base remains limited by short study durations, small sample sizes, and a lack of fertility-focused endpoints. Concerning GLP-1 agonists, a randomized, double-blind crossover trial in healthy lean men demonstrated that dulaglutide had no effect on sexual desire, hypothalamic–pituitary–gonadal axis hormones, or semen parameters, suggesting no direct impairment of male sexual or reproductive function. However, recent clinical case reports have identified possible female sexual side effects [95]. Another aspect that has to be taken into account is the pharmacokinetic behavior of semaglutide that is determined by its rational structural modifications, including DPP-4-resistant amino acid substitution and fatty-acid side-chain conjugation that promotes reversible albumin binding; these features prolong systemic exposure, support attainment of steady-state concentrations after repeated dosing, and may influence its distribution into extracellular compartments, although the extent and clinical relevance of semaglutide penetration into compartments such as the ovarian follicular fluid require further investigation [96,97,98]. These findings further accentuate the need for large-scale, well-designed clinical trials to clarify the direct effects of semaglutide, and other well-applied GLP-1 agonists, on ovarian function, folliculogenesis, and the hypothalamic–pituitary–ovarian axis. Importantly, GLP-1 receptor agonists are not currently approved or guideline-recommended for fertility restoration, and substantial uncertainty remains regarding long-term reproductive safety, optimal patient selection, and clinical guidelines [99]. Until such data are available, treatment decisions should be individualized, considering metabolic status, reproductive goals, and careful clinical oversight. Figure 4 illustrates the mechanisms of action, dosage escalation, metabolic and reproductive effects, side effects, and contraindications of semaglutide in women with obesity.

In conclusion, semaglutide demonstrates significant therapeutic potential for obese women by driving profound weight reduction and reversing metabolic dysfunction. These metabolic improvements offer promising indirect benefits for female reproductive health, particularly in restoring menstrual regularity and improving ovulatory function in conditions like PCOS. However, clinical data focused specifically on reproductive and fertility endpoints remain strictly limited, characterized by small sample sizes and short study durations. Because current evidence regarding direct mechanisms on the hypothalamic–pituitary–ovarian axis and follicular microenvironment is still emerging, caution is warranted. Semaglutide remains strictly contraindicated during pregnancy due to safety concerns identified in animal studies. Therefore, while semaglutide serves as an effective pre-pregnancy weight management strategy, large-scale, long-term clinical trials are urgently required to establish its direct impact on fertility, clarify optimal washout periods, and formulate definitive clinical guidelines for reproductive-aged women.

### 4.4. Phentermine and Topiramate Combination (Qsymia)

Phentermine (2-methyl-1-phenylpropan-2-amine) belongs to the class of sympathomimetic drugs and acts on the hypothalamus by enhancing the release of neurotransmitters such as serotonin, norepinephrine, and dopamine [1,77]. Through this mechanism, it reduces the appetite and diminishes the feeling of pleasure associated with food consumption [12]. Increased release of norepinephrine in the brain contributes to the enhancement of leptin production [12]. In 1995, the FDA approved phentermine as a medication for the treatment of obesity, with its use being limited to a short-term period of up to 12 weeks [12]. Common side effects of phentermine include insomnia, dry mouth, increased blood pressure, and tachycardia [12]. In addition, due to its chemical similarity to amphetamines, there is a potential risk of developing dependence [12]. Its use is not recommended in pregnant women, nor in individuals with glaucoma, uncontrolled hypertension, or a history of cardiovascular disease [12].

Topiramate acts through three main mechanisms: (i) it enhances the activity of gamma-aminobutyric acid (GABA), (ii) it inhibits the action of glutamic acid, and (iii) it inhibits the function of carbonic anhydrase [12,77]. Topiramate is used both in the treatment of epilepsy, due to its anticonvulsant properties, and in the prevention of migraines [12]. At the same time, it has been observed to contribute to a reduction in appetite [12]. Topiramate should not be administered during pregnancy, nor in patients with glaucoma or a history of kidney stones [12]. The use of topiramate during pregnancy has been associated, in experimental animal studies, with an increased risk of fetal mortality, and a higher likelihood of cleft lips in fetuses has also been reported [67]. In addition, its use is not recommended during breastfeeding, as adverse effects such as diarrhea and drowsiness have been observed in infants breastfed by mothers receiving topiramate treatment [67]. Topiramate may also interact with combined oral contraceptives, potentially reducing their contraceptive effectiveness and increasing the likelihood of bleeding [67]. Furthermore, topiramate’s effects on neurotransmitter pathways, particularly on GABA receptors, appear to potentially influence normal reproductive function. In experimental rat models, possible effects have been observed on ovulatory cycles, oocyte quality, and the implantation process, which may lead to reduced fertility [68].

The combination of phentermine and topiramate (Qsymia) was approved by the FDA in 2012 as a pharmacological treatment for obesity [67]. Khera and colleagues conducted a systematic review and meta-analysis that included 28 randomized clinical trials with a total of 29,018 participants. The mean age was 46 years, 74% of the patients were women, and the average body weight and BMI were 100.5 kg and 36.1 kg/m^2^, respectively. The study compared pharmacological interventions with placebo, including orlistat (16 trials), lorcaserin (3 trials), naltrexone–bupropion (4 trials), phentermine–topiramate (2 trials), and liraglutide (2 trials). In addition, a multi-arm trial comparing both liraglutide and orlistat with placebo was included. It was found that, compared with placebo, the greatest mean weight loss after one year of treatment was observed with the phentermine–topiramate combination, resulting in an average reduction of approximately 8.8 kg (95% CI: −10.2 to −7.42 kg). Moreover, considering that about 9% of patients on placebo achieved at least 10% weight loss, the probability of reaching this threshold was substantially higher with active treatments: 54% with phentermine–topiramate, 34% with liraglutide, 30% with naltrexone–bupropion, 25% with lorcaserin, and 20% with orlistat [95]. These findings suggest that among pharmacological interventions for the treatment of obesity, the phentermine–topiramate combination is the most effective option, achieving the greatest mean weight loss and the highest rates of clinically significant (>10%) reduction compared with placebo and other treatments [100].

Qsymia is contraindicated in all cases where the administration of phentermine or topiramate is not recommended, as it is a combination of these two active substances. Caution is required in women of reproductive age, as the drug is classified as Category X for use during pregnancy [12]. For this reason, clinical guidelines recommend performing a pregnancy test before initiating treatment and repeating it every month during the therapy to avoid potential fetal exposure during the early stages of pregnancy [68]. At the same time, many healthcare professionals require patients to provide written confirmation of consistent use of reliable contraception methods throughout the treatment [68].

The most common adverse effects associated with phentermine/topiramate therapy include paresthesia, hypoesthesia, dry mouth, constipation, taste disturbances, decreased concentration, irritability, dizziness, insomnia, and increased blood pressure [12]. Overall, Qsymia, as a combination of phentermine and topiramate, appears to be the most effective pharmacological option for the management of obesity in women of reproductive age, due to the substantial weight loss it achieves. However, its use is associated with important limitations, particularly regarding pregnancy, as well as potential effects on reproductive mechanisms. Despite its efficacy in reducing body weight, its impact on female fertility remains insufficiently documented and requires further long-term investigation, making careful and individualized use in clinical practice essential [77]. However, due to its adverse effects, the potentiality of the combination in female reproduction is not considered promising.

### 4.5. Bupropion and Naltrexone Combination (Contrave)

Bupropion is a selective catecholamine reuptake inhibitor, primarily targeting norepinephrine and dopamine, two neurotransmitters that play an important role in the regulation of mood and the brain’s reward system [12,68]. It belongs to the class of aminoketone antidepressants and is widely used for the treatment of depression and nicotine dependence, while in some cases it is also used as an adjunct therapy for the management of attention-deficit disorder with or without hyperactivity (ADHD/ADD) [68]. The enhancement of dopaminergic and noradrenergic neurotransmission by bupropion contributes to improved mood, increased energy, and reduced fatigue [68]. At the same time, the drug stimulates pro-opiomelanocortin (POMC) neurons in the arcuate hypothalamic nucleus (ARH) [12]. In these neurons, POMC is cleaved into alpha-melanocyte-stimulating hormone (α-MSH) and beta-endorphins [12,96]. α-MSH acts on melanocortin-4 receptors (MC4R), leading to the induction of satiety, reduced food intake, and increased energy expenditure [12]. In contrast, beta-endorphins bind to μ-opioid receptors (MORs) located on POMC neurons. Activation of these receptors triggers a self-inhibitory negative feedback mechanism, which limits the continued release of α-MSH [12,101]. Although bupropion is primarily used as an antidepressant, it has been observed that its administration may also contribute to weight loss, a finding that supports its investigation as a potential treatment for obesity. In an 8-week randomized, double-blind, placebo-controlled clinical trial, Gadde et al. studied 50 overweight and obese women with a BMI ranging from 28.0 to 52.6 kg/m^2^. Bupropion treatment was initiated at a dose of 100 mg per day and gradually increased to a maximum dose of 200 mg twice daily. In addition, all participants followed a hypocaloric diet of 1600 kcal/day, and adherence was monitored through food diaries. Women who responded to the treatment continued receiving it in a double-blind manner for an additional 16 weeks, completing a total of 24 weeks of follow-up. Participants who received bupropion showed a mean body weight loss of 6.2% ± 3.1% after 8 weeks of treatment (n = 18), which increased to 12.9% ± 1.5% after 24 weeks of treatment (n = 14) [102]. At the same time, obesity has been associated with an increased risk of developing depression and anxiety disorders [103]. Pathophysiological mechanisms such as chronic inflammation, insulin and leptin resistance, as well as hypertension, appear to contribute both to metabolic dysfunction and to the development of psychiatric disorders [103]. In addition, among women with obesity, the prevalence of depression is approximately twice as high compared to men [67]. For this reason, the antidepressant effects of bupropion may provide additional therapeutic benefit in this population [67].

Naltrexone is a synthetic, long-acting opioid receptor antagonist and is primarily used in the treatment of alcohol and opioid dependence [12,68]. Its pharmacological action is based on the blockade of μ-opioid receptors (MORs) in the brain. Through this mechanism, the pleasurable and rewarding effects associated with food intake are reduced, which contributes to appetite suppression [12,68]. The FDA has approved the use of naltrexone for the treatment of alcohol and opioid dependence, as well as for the management of severe opioid toxicity [12]. Experimental data have shown that naltrexone may contribute to reduced food intake, overall food consumption, and binge-eating episodes. However, in clinical studies conducted in humans, its use as monotherapy has not consistently produced reliable and reproducible results in terms of weight loss [104]. Ahmed et al. also described beneficial effects of naltrexone in women with PCOS. In a randomized controlled trial, 30 obese, infertile patients with hyperandrogenism and hyperinsulinemia received oral naltrexone at a dose of 50 mg daily for 6 months [100]. In cases where ovulation was not achieved after 12 weeks of monotherapy, clomiphene citrate was added (50 mg/day for 5 days, with the option to increase up to 150 mg/day in non-responders). Naltrexone treatment was associated with a significant reduction in body mass index, fasting insulin levels, the LH/FSH ratio, and androgen levels. No pregnancies were observed during monotherapy, whereas during the combination phase with clomiphene, 9 women (33.3%) achieved pregnancy [105]. In addition, Fulghesu et al. demonstrated that oral administration of naltrexone at a dose of 50 mg daily for 8 weeks may enhance the effectiveness of pulsatile GnRH (5 μg/bolus every 90 min) administration in obese women with PCOS. Specifically, this combination was associated with higher rates of ovarian response and ovulation induction (90%) compared with pulsatile GnRH administered as monotherapy (60%) [106].

**Figure 5:**
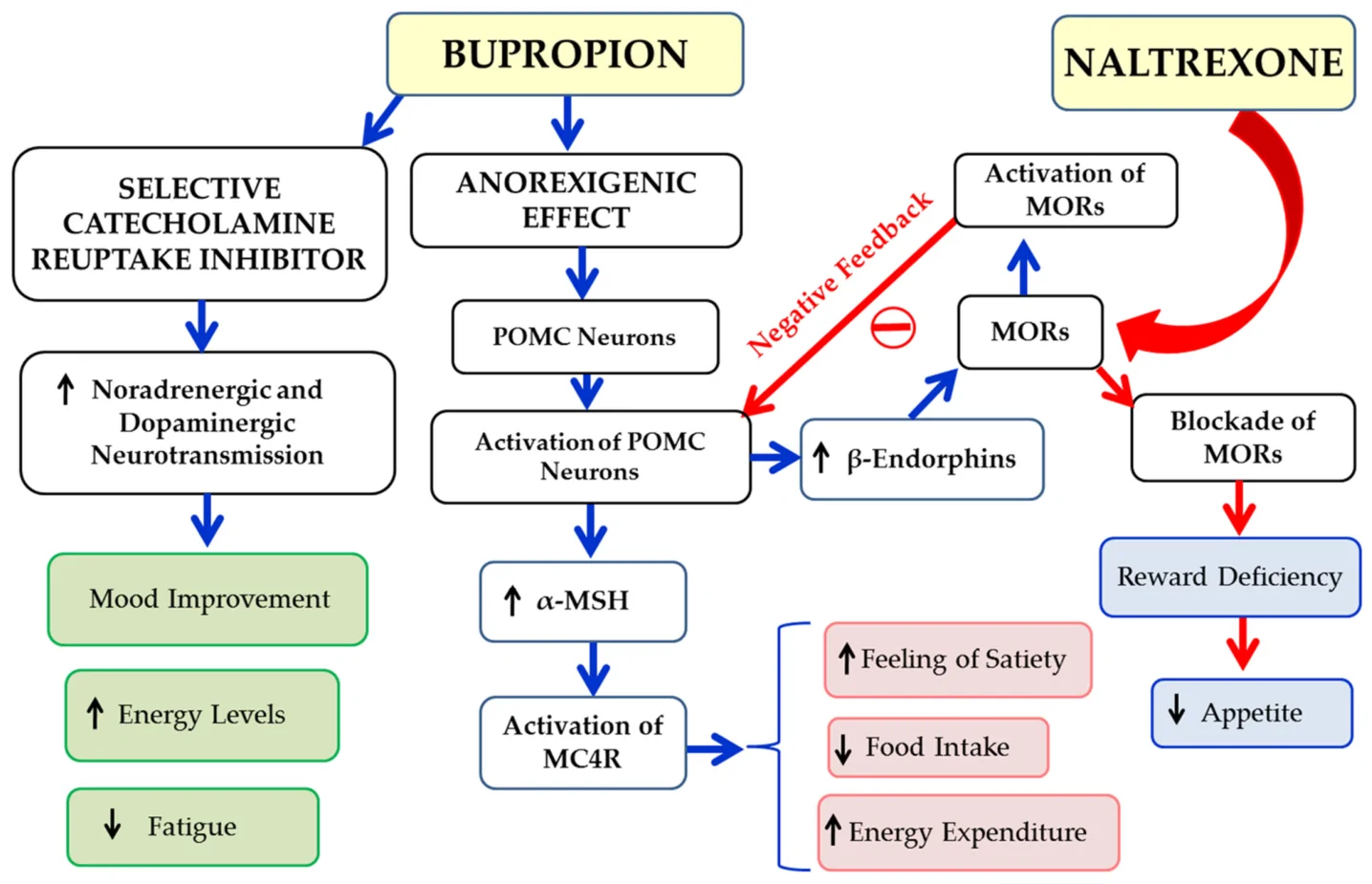
Mechanism of action of the bupropion/naltrexone combination in the hypothalamic melanocortin system through activation of POMC neurons and enhancement of satiety. Bupropion is a selective catecholamine reuptake inhibitor that primarily targets norepinephrine and dopamine, enhancing dopaminergic and noradrenergic neurotransmission. This mechanism contributes to improved mood, increased energy, and reduced fatigue. In parallel, bupropion exerts anorexigenic effects by stimulating pro-opiomelanocortin (POMC) neurons in the arcuate hypothalamic nucleus (ARH). Within these neurons, POMC is cleaved into α-melanocyte-stimulating hormone (α-MSH) and β-endorphin. α-MSH activates melanocortin-4 receptors (MC4R), promoting satiety, reducing food intake, and increasing energy expenditure. Conversely, β-endorphin binds to MORs on POMC neurons, triggering an autoinhibitory negative feedback mechanism that limits further α-MSH release and leads to reward deficiency and reduced appetite (α-MSH: alpha-melanocyte-stimulating hormone; MC4R: melanocortin-4 receptor; POMC: pro-opiomelanocortin; MOR: μ-opioid receptor).

The pharmaceutical formulation Contrave, which combines naltrexone and bupropion, exploits the synergistic action of the two active substances, achieving greater effectiveness in weight loss compared with the administration of each agent alone. This combination was approved by the FDA in 2014 for the pharmacological treatment of obesity [12,67,68]. This treatment is indicated for obese individuals with a body mass index (BMI) > 30 kg/m^2^, as well as for overweight patients with a BMI > 27 kg/m^2^ who present at least one obesity-related comorbidity, such as type 2 diabetes mellitus (T2DM), hypertension, or dyslipidemia [12]. At the same time, its administration is recommended to be accompanied by a hypocaloric diet and increased physical activity to enhance the overall effectiveness of the therapeutic intervention [12]. Bupropion stimulates the activity of POMC neurons in the arcuate nucleus of the hypothalamus, increasing POMC production and the release of α-MSH and β-endorphin [12,68]. α-MSH acts on MC4R receptors, causing a feeling of satiety, reduced food intake, and increased energy expenditure. However, β-endorphins, which are endogenous agonists of MORs on POMC neurons, activate negative feedback mechanisms, limiting the further secretion of α-MSH [12]. At this point, naltrexone acts by blocking MORs, thereby preventing the β-endorphin-mediated autoinhibitory feedback of POMC neurons on one hand and on the other contributing to the inhibition of reward pathways and the reduction in hedonic food intake [12,67,101] (Figure 5).

In the COR-BMOD (Behavioral Modification) study, the effectiveness of the naltrexone/bupropion combination was evaluated in conjunction with an intensive behavioral modification program. Participants who received the pharmacological treatment alongside the behavioral intervention experienced significantly greater weight loss compared with the group following the same program while receiving placebo (−11.5% ± 0.6% vs. −7.3% ± 0.9%, *p* < 0.001) [107]. In the randomized, double-blind, placebo-controlled COR-II clinical trial by Apovian et al., 1496 obese and overweight adults with dyslipidemia and/or hypertension participated. The participants received a combination of sustained-release (SR) naltrexone 32 mg/day and sustained-release bupropion 360 mg/day (NB32) for up to 56 weeks. Treatment with NB32 was associated with a significantly greater reduction in body weight compared with placebo (−8.2% vs. −1.4%). In addition, more pronounced improvements in cardiometabolic risk markers were observed compared with the placebo group [108]. In the COR-Diabetes study by Lee Hollander et al., overweight and obese patients with type 2 diabetes mellitus received either a combination of sustained-release naltrexone 32 mg and sustained-release bupropion 360 mg for a period of 56 weeks. The results showed that administration of the combined therapy was associated with a significantly greater reduction in body weight compared with placebo (−5.0% vs. −1.8%, *p* < 0.001). At the same time, a greater proportion of patients in the intervention group achieved at least 5% weight loss compared with the control group (44.5% vs. 18.9%, *p* < 0.001) [109]. The systematic review and meta-analysis by Liu examined data from randomized controlled clinical trials in order to evaluate the effect of bupropion, both as monotherapy and in combination with naltrexone, on body weight indices in overweight or obese adults. A total of 25 studies (33 treatment arms) were included in the analysis, involving participants aged 18 years and older. The interventions included comparisons of bupropion alone and bupropion combined with naltrexone versus placebo or other control groups. The primary outcomes concerned body weight, BMI, and waist circumference. The results showed that both bupropion and, to a greater extent, its combination with naltrexone led to a statistically significant reduction in body weight (WMD: −3.67 kg) and waist circumference (WMD: −2.98 cm) compared with the control groups, although no significant effect on BMI was observed. Subgroup analyses demonstrated greater weight loss when treatment duration exceeded 26 weeks, as well as the superiority of combination therapy over bupropion monotherapy. Finally, meta-regression analysis showed that higher doses were associated with greater reductions in body weight [110].

Administration of Contrave is carried out through gradual dose titration, aiming to improve patient tolerability and maximize therapeutic effectiveness. This gradual dose adjustment assists in the reduction in the adverse effects that may occur during the treatment [68]. The most-reported adverse effects associated with the administration of the naltrexone/bupropion combination include nausea, constipation, headache, dizziness, insomnia, dry mouth, diarrhea, and increased blood pressure [12]. The use of this combination is not recommended in individuals with a history of seizures or eating disorders such as bulimia or anorexia nervosa, while concurrent alcohol consumption is also contraindicated [12]. In addition, its concomitant use with opioids, other medications containing bupropion, or monoamine oxidase inhibitors (MAOIs) within the previous 14 days is contraindicated [68]. Additionally, Contrave is contraindicated in patients with uncontrolled hypertension, as well as during pregnancy and breastfeeding, since both naltrexone and bupropion are excreted in breast milk [12,68]. In studies conducted in pregnant rats and rabbits, oral administration of naltrexone at doses higher than the therapeutic range was associated with an increased incidence of early fetal loss [67]. According to data from the U.S. FDA, most available studies in humans and experimental animals do not demonstrate an increased risk of adverse pregnancy outcomes associated with the use of bupropion [67]. Nevertheless, some experimental data from animal studies have reported reduced fetal body weight and a higher incidence of fetal abnormalities when supratherapeutic doses were administered [67]. In addition, there are a few case reports of seizures in infants who were breastfed by mothers receiving bupropion treatment [67]. The systematic review by Goldberg et al. found no direct clinical trials evaluating the combination of bupropion and naltrexone specifically in women with polycystic ovary syndrome (PCOS) [18]. The authors described the combination as a potential future therapeutic option for obesity in PCOS, given that obesity and insulin resistance are key pathogenetic factors of the disorder. Available data from the general obese population suggests approximately 5% weight loss after 12 weeks of treatment. Since weight reduction in PCOS is associated with improvement in metabolic and reproductive parameters, the combination may theoretically benefit insulin resistance, hormonal balance, and ovulation. However, it is emphasized that specific randomized trials in PCOS are lacking, and the efficacy and safety of Contrave in this population have not been adequately established. Therefore, further high-quality clinical trials are required to evaluate its long-term metabolic, reproductive, and psychological effects [18]. Overall, the naltrexone/bupropion combination (Contrave) has primarily been studied as a treatment for obesity rather than specifically for reproductive outcomes; however, its potential effect on female reproductive function arises indirectly through significant weight loss and improvements in metabolic parameters. Weight reduction in obese women may lead to improved insulin resistance, regulation of hormonal disturbances, and restoration of ovulation, factors that are particularly important in PCOS. Data from clinical studies show that the combination achieves meaningful weight loss and improvements in cardiometabolic markers, which could theoretically support better reproductive function. In addition, bupropion may contribute to improved mood, which can positively influence sexual and psychological health. Nevertheless, there are still no specific clinical studies demonstrating a direct effect of Contrave on fertility or reproductive outcomes in women with obesity or PCOS, making the current evidence largely indirect and theoretical.

## 5. Off-Label Prescribing of Anti-Obesity Medications

### 5.1. Metformin

**Figure 6:**
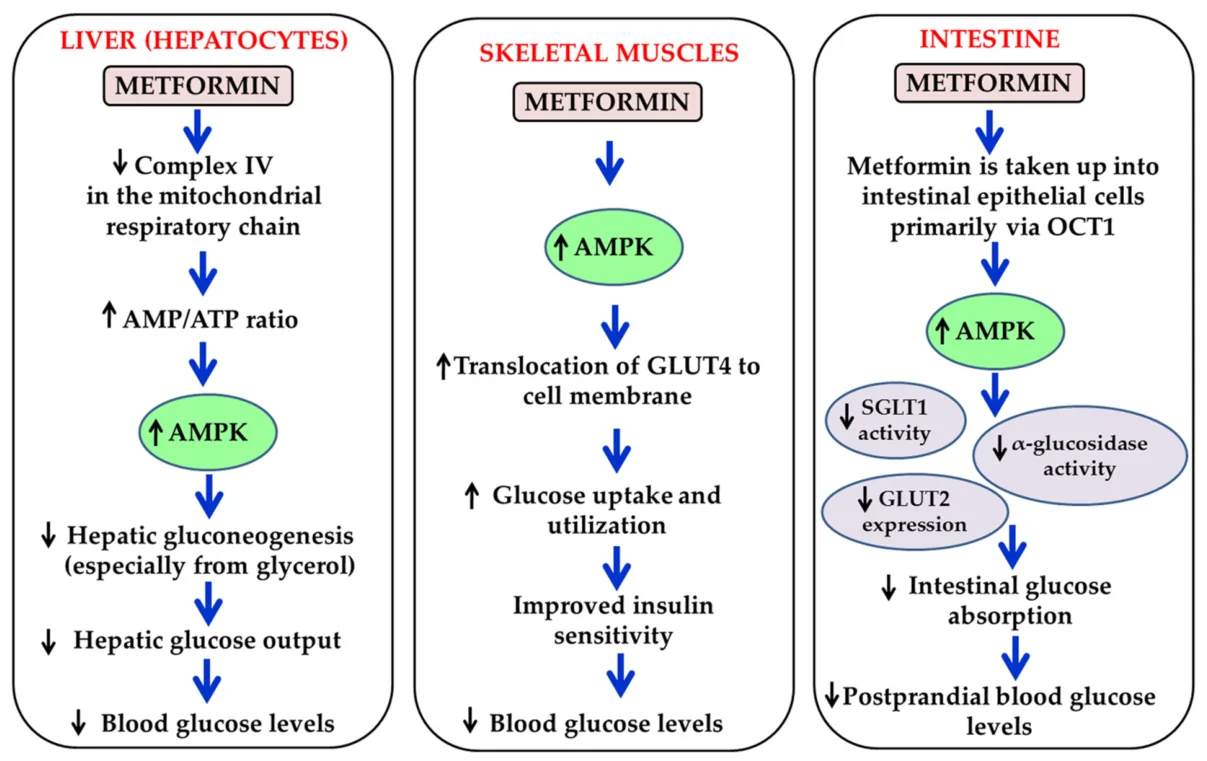
Illustration of the mechanisms by which metformin acts in hepatocytes, skeletal muscles and the intestine and reduces blood glucose levels (GLUT2: Glucose Transporter 2; GLUT4: Glucose Transporter 4; OCT1: Organic Cation Transporter 1; SGLT1: sodium-glucose cotransporter 1).

Metformin is an oral antihyperglycemic drug, derived from the plant Galega officinalis (known as “goat gall” or “French lilac”) [14]. It belongs to the biguanide class and was approved by the FDA in 1995 for the treatment of type 2 diabetes mellitus [14]. Metformin works primarily by reducing glucose production by the liver, improving the sensitivity of peripheral tissues to insulin, and reducing intestinal glucose absorption, ultimately resulting in a reduction in blood glucose levels [14,67]. This mechanism appears to be related to the inhibition of the function of complex IV of the mitochondrial respiratory chain, which alters the cytoplasmic redox balance and leads to selective inhibition of hepatic gluconeogenesis, especially that derived from glycerol [14]. At the same time, at the cellular level, metformin activates AMP-activated protein kinase (AMPK), thus contributing to further suppression of hepatic gluconeogenesis and enhancement of glucose uptake by skeletal muscles [14]. As illustrated in Figure 6, metformin exerts its glucose-lowering effects by precise molecular cascades in hepatocytes, skeletal muscle, and the intestine: In hepatocytes, the inhibition of complex IV increases the intracellular AMP/ATP ratio, which serves as the direct trigger for AMPK-mediated reductions in hepatic glucose output. In skeletal muscles, the activated AMPK induces the translocation of GLUT4 transporters to the cell membrane, facilitating enhanced peripheral glucose uptake and utilization. In the intestine, metformin enters epithelial cells via the OCT1 transporter, where it utilizes AMPK to downregulate both SGLT1 and α-glucosidase activity while suppressing GLUT2 expression, effectively minimizing postprandial glucose absorption.

**Figure 7:**
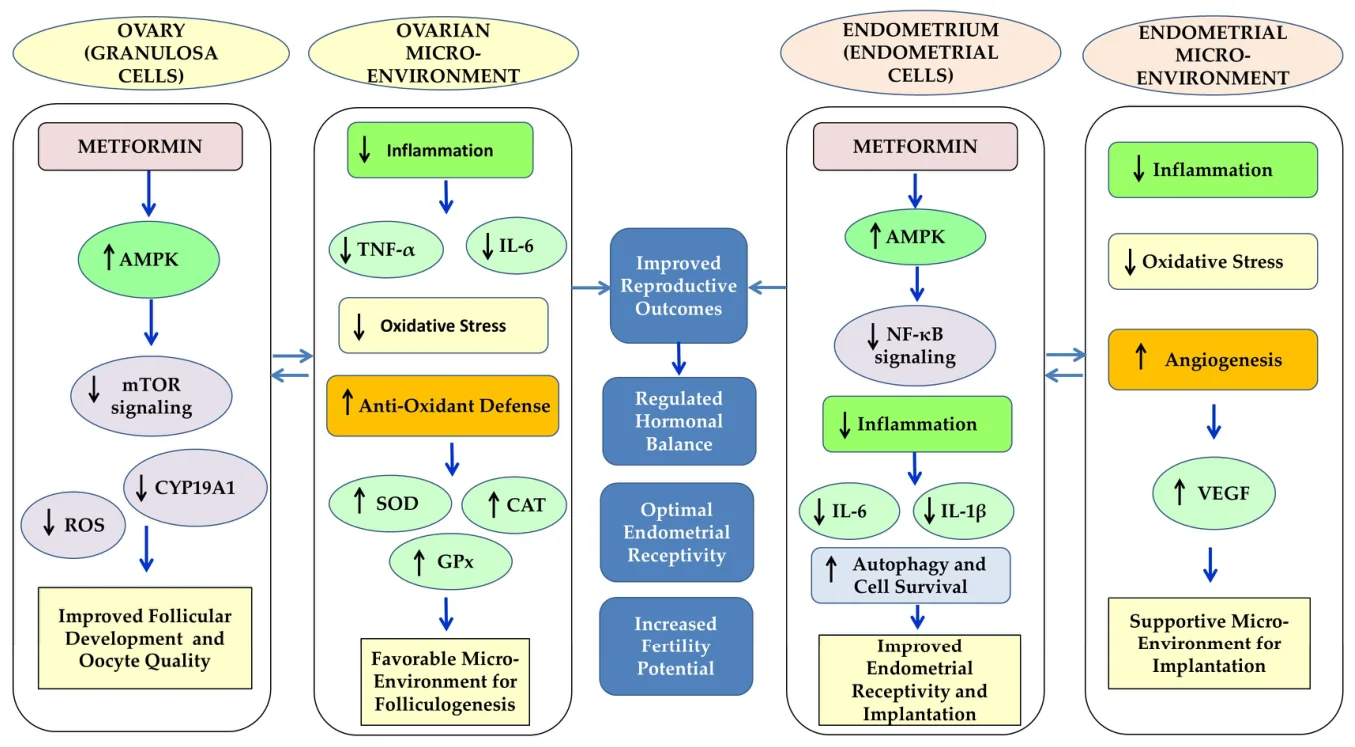
Schematic representation of the molecular mechanisms and microenvironmental modulations of metformin in the ovary and endometrium leading to improved reproductive outcomes (AMPK: adenosine monophosphate-activated protein kinase; CAT: catalase; CYP19A1: cytochrome P450 family 19 subfamily A member 1 (aromatase); GPx: glutathione peroxidase; IL-1β: interleukin-1 beta; IL-6: interleukin-6; mTOR: mammalian target of rapamycin; NF-κB: nuclear factor kappa B; ROS: reactive oxygen species; SOD: superoxide dismutase; TNF-α: tumor necrosis factor-alpha; VEGF: vascular endothelial growth factor).

At the ovarian level, available data suggest that metformin may indirectly modulate steroidogenesis by attenuating insulin-driven theca cell androgen production; however, direct in vivo evidence delineating specific ovarian cellular targets in humans remains limited [111,112]. Beyond these systemic pathways, molecular evidence indicates that metformin acts directly on granulosa cells by activating the adenosine monophosphate-activated protein kinase (AMPK) pathway [113,114]. This activation subsequently downregulates mammalian target of rapamycin (mTOR) signaling, leading to a decrease in reactive oxygen species (ROS) and a downregulation of cytochrome P450 19A1 (CYP19A1), which ultimately improves follicular development and oocyte quality [115,116]. Concurrently, within the ovarian microenvironment, metformin mitigates inflammation—demonstrated by the reduction in tumor necrosis factor-alpha (TNF-α) and interleukin-6 (IL-6)—while lowering oxidative stress and enhancing antioxidant defenses via the upregulation of superoxide dismutase (SOD), catalase (CAT), and glutathione peroxidase (GPx) to foster a favorable microenvironment for folliculogenesis [117] (Figure 7). Similarly, in the endometrium, accumulating evidence indicates that metformin may improve markers of endometrial receptivity, including vascular parameters and implantation-related gene expression, as well as reduce inflammatory signaling in PCOS populations [111,118]. At the cellular level, metformin-mediated AMPK activation suppresses nuclear factor kappa B (NF-κB) signaling, thereby decreasing inflammatory cytokines such as IL-6 and interleukin-1 beta (IL-1β) while promoting autophagy and cell survival to improve endometrial receptivity and implantation [119,120]. Within the local endometrial microenvironment, metformin additionally suppresses oxidative stress and inflammation while driving angiogenesis through increased vascular endothelial growth factor (VEGF) expression [121]. Collectively, these coordinated cellular and microenvironmental enhancements converge to deliver improved reproductive outcomes, characterized by a regulated hormonal balance, optimal endometrial receptivity, and increased fertility potential [122]. Nevertheless, the extent to which these effects reflect direct endometrial actions versus secondary improvements mediated by systemic metabolic and endocrine normalization remains incompletely understood and represents an active area of ongoing research [123] (Figure 7).

Metformin may also cause mild weight loss in overweight individuals, diabetic or not, primarily through improved insulin sensitivity, which may contribute to reduced appetite and lower energy intake [14,67]. The systematic review by Lentferink et al. (2018) [124] evaluated the efficacy of metformin in overweight and obese children and adults in the reduction in the body weight, insulin resistance, and the prevention of the progression to type 2 diabetes mellitus. The results showed that the effect of metformin on body weight and BMI is variable, with smaller reductions being observed in children compared to adults. At the same time, it was found that the use of metformin in adults is associated with a significant reduction in the risk of progression to T2DM, with percentages ranging from 7% to 31% [124]. There is also evidence that metformin may affect the composition of the intestinal microflora, contributing to body weight control [14]. However, metformin has not been officially approved as a treatment for weight loss and its use for this purpose requires careful consideration by healthcare professionals [14]. Metformin may enhance the beneficial effects of liraglutide in improving insulin sensitivity and glucose regulation. In a 12-week randomized trial by Jensterle et al. in 30 obese women with PCOS, the combination of metformin (1000 mg twice daily) and liraglutide (1.2 mg daily) was associated with significant reduction in body weight and glucose levels after a glucose tolerance test, whilst total androgens were decreased and less nausea was observed. In contrast, liraglutide monotherapy (3 mg daily) appeared to be more effective in terms of weight loss compared to the combination [125]. Metformin can also be used in other clinical conditions, such as PCOS, certain types of cancer, type 1 diabetes mellitus, and insulin resistance. In addition, its potential role in interventions related to slowing the aging process is also being investigated [14]. Metformin administration contributes to the restoration of ovarian function through the improvement of metabolic and hormonal disorders. According to Magzoub R et al. in women with polycystic ovary syndrome who are not obese, metformin is associated with a modest improvement in clinical pregnancy rates compared to placebo [15]. At the same time, it was found that its effectiveness is similar to clomiphene citrate in achieving clinical pregnancy; however, it is accompanied by an increased likelihood of miscarriage [15]. In adult women with polycystic ovary syndrome, especially those with a body mass index of 25–30 kg/m^2^, its effectiveness in reducing hirsutism appears to be limited compared to oral contraceptives [14]. In women with PCOS, the combination of metformin and oral contraceptives may enhance androgen regulation and improve menstrual cycles, particularly in obese patients. However, in women with normal or low body weight, it may cause adverse effects, such as decreased muscle mass and fluid retention (the “osteosarcopenic effect”) [16]. This phenomenon appears to result from a combination of the hormonal and metabolic effects of the treatment. The reduction in androgens limits the maintenance of muscle mass, while the activation of AMPK by metformin enhances catabolic processes in muscle tissue. At the same time, changes in mitochondrial function and the increase in muscle damage markers contribute to muscle loss and functional decline, leading to this phenotype. Therefore, an individualized therapeutic approach is necessary [16]. Metformin can be used as a second-line treatment in combination with gonadotropins for ovulation induction in assisted reproduction programs, such as IVF and ICSI. It acts by improving metabolic and endocrine parameters, reducing androgen levels, and enhancing ovarian response and implantation, while reducing the need for high doses of gonadotropins and preventing ovarian hyperstimulation syndrome (OHSS) [14,16]. An analysis of nine randomized trials in 816 women with polycystic ovary syndrome showed that metformin increases clinical pregnancy rates and reduces the risk of ovarian hyperstimulation syndrome. However, there was no clear evidence that it improves live birth rates (LBR) [126]. Similarly, the double-blind randomized study by Abdalmageed et al. showed that the use of metformin in overweight or obese women with PCOS undergoing IVF was associated with a lower number of oocytes at oocyte retrieval, without a clear improvement in live birth rates (LBR) [127]. Also, in the context of assisted reproduction, the available data on the effect of metformin on the risk of spontaneous abortions remain conflicting [16]. In women with PCOS, metformin has been studied in combination with clomiphene citrate, a mild nonsteroidal estrogen antagonist, for the treatment of anovulatory infertility [16]. However, the effectiveness of the combination of metformin and clomiphene citrate in inducing ovulation and improving birth rates, compared with clomiphene citrate monotherapy, remains controversial [128]. The combination of metformin and clomiphene citrate appears to be more effective in women with PCOS who have a reduced response to clomiphene citrate, severe obesity, insulin resistance, or dyslipidemia, as well as in those who respond positively to metformin [16]. Also, in women with PCOS, the combination of metformin and letrozole, which is an aromatase inhibitor used to restore the ovarian cycle and induce ovulation, appears to improve pregnancy and live birth rates compared to the combination of clomiphene citrate and metformin [16]. Agrawal et al. (2019) showed that the combination of metformin with myo-inositol, a natural sugar of the B vitamin complex, is more effective in inducing ovulation in women with polycystic ovary syndrome and infertility and is also associated with higher live birth rates [129]. In some cases of metformin use, adverse gastrointestinal effects have been reported [14,16]. The FDA recommends that, during pregnancy, women with diabetes should receive insulin, while data regarding the safety of metformin during pregnancy remain limited [67,114]. In the study by Dukhovny et al., exposure to metformin during the first trimester of pregnancy was investigated in both women with diabetes and non-diabetic women who used it for the treatment of infertility. The results showed that its use in pregnant women with diabetes was associated with an increased risk of various congenital anomalies related to diabetes. When metformin was used for the treatment of infertility, the adjusted odds ratios (aORs) for secundum atrial septal defect, anorectal malformations, and limb reduction defects were increased by 2–4 times [130]. In contrast, in the study by Notaro et al., adverse effects of metformin during pregnancy were characterized mainly as mild, with no findings linking its use to an increased likelihood of teratogenesis [131]. Overall, metformin is a widely used antihyperglycemic drug with multidimensional action on glucose metabolism, through reduction in hepatic gluconeogenesis, enhancement of insulin sensitivity and activation of AMPK. In addition to its main indication in type 2 diabetes mellitus, it has an important role in metabolic and reproductive disorders, such as PCOS, where it contributes to the regulation of androgens, improvement in ovarian function and restoration of the cycle. Despite the favorable results on indicators such as ovulation, insulin resistance and clinical pregnancy, the data on live births remain contradictory. Furthermore, its action in combination therapies (liraglutide, clomiphene, letrozole, inositol) show that its effectiveness depends on the patient’s profile. Therefore, its use requires an individualized approach and careful clinical evaluation.

### 5.2. Exenatide

Exenatide is the first representative of the class of glucagon-like peptide-1 (GLP-1) receptor agonists to be introduced into clinical practice [132]. Its development was based on the study of exendin-4, a peptide isolated from the saliva of the venomous lizard *Heloderma suspectum* [133]. Exendin-4 shows significant structural similarity to endogenous GLP-1, which allows it to bind to GLP-1 receptors and mimic its physiological effects in the human body [134]. Through activation of these receptors, exenatide enhances glucose-dependent insulin secretion, thereby improving glycemic control with a low risk of hypoglycemia [135]. At the same time, it suppresses inappropriate glucagon secretion from pancreatic α-cells, reducing hepatic glucose production [135]. In addition, it slows gastric emptying, leading to delayed postprandial glucose absorption [136], while also exerting central effects that reduce appetite and consequently energy intake [137]. The combination of these actions makes exenatide particularly useful for both glycemic control and body weight reduction [77]. Exenatide was approved by the FDA in 2005 for the treatment of type 2 diabetes mellitus, becoming one of the first GLP-1 receptor agonists introduced into clinical practice [138]. Subsequently, as evidence accumulated regarding its beneficial effects on weight reduction, its use expanded to the management of obesity, particularly in patients with associated metabolic disorders. However, exenatide has not received formal FDA approval for obesity [139]. Exenatide is administered subcutaneously, typically initiated at a dose of 5 micrograms twice daily, which may be increased to 10 micrograms twice daily after one month if needed for glycemic control, depending on patient response and tolerability [140]. This gradual titration optimizes therapeutic efficacy while minimizing adverse effects [139]. The most frequently reported side effects of exenatide involve the gastrointestinal system. In addition, patients may experience injection-site induration, headaches, and, less commonly, hypersensitivity reactions [141]. In the study by Rosenstock et al. (2010) [142], the effect of exenatide in combination with lifestyle interventions on body weight and glucose tolerance was evaluated in obese individuals, with or without prediabetes, compared with placebo over a 24-week period. The results showed that the exenatide group achieved significantly greater weight loss (−5.1 ± 0.5 kg) compared to the placebo group (−3.3 ± 0.5 kg). In addition, favorable changes in glucose tolerance were observed, particularly in individuals with prediabetes. Overall, the study supports that exenatide, when combined with lifestyle modification, enhances weight loss and improves metabolic parameters, highlighting its role as an adjunct pharmacological intervention in the management of obesity [142]. The double-blind trial by Lundkvist et al. (2017) [143] evaluated the effects of once-weekly exenatide (2 mg) in combination with dapagliflozin (10 mg once daily) on body weight, body composition, glycemic variables, and systolic blood pressure (SBP) in obese adults without diabetes, compared with placebo, over a 24-week period. Regarding body weight, the difference in weight change between the exenatide plus dapagliflozin group and placebo was −4.13 kg. In addition, 36% of participants in the active treatment group achieved a weight loss of ≥5% of their baseline body weight, compared with only 4.2% in the placebo group, indicating clinically meaningful efficacy in weight reduction. Prediabetes was also less frequent in the active treatment group. Furthermore, the difference in change in systolic blood pressure (SBP) between the exenatide plus dapagliflozin group and placebo was −6.7 mmHg. Overall, the results suggest that the combination therapy of exenatide and dapagliflozin leads to a moderate but clear reduction in body weight in obese adults without diabetes, mainly through a reduction in fat mass, with good tolerability. In addition, favorable changes in glycemic parameters were observed, including a possible reduction in the incidence of prediabetes, as well as a decrease in systolic blood pressure [143]. In the randomized, double-blind, placebo-controlled study by Basolo et al. (2018) [144], the effect of subcutaneous administration of exenatide (10 μg twice daily) on energy expenditure, energy intake, and body weight was evaluated in obese adults without diabetes. The study duration was 24 weeks, with interim monitoring during the first 5 weeks and monthly visits until completion. The results showed that exenatide significantly reduced spontaneous energy intake (primarily in the short term), without affecting 24 h energy expenditure. Greater initial weight loss was observed (at 5 weeks), but no significant difference was found at 6 months compared to placebo. Overall, the study demonstrates that exenatide reduces food intake and limits overeating but does not substantially affect long-term energy expenditure or body weight [144]. In the study by Rui-Li Ma et al. (2021) [145], the effectiveness of the combination of exenatide (2 mg QW, once weekly) and metformin (500 mg three times a day) was evaluated in overweight or obese women with PCOS over a period of 12 weeks. The results showed that adding exenatide to metformin therapy led to a significant reduction in body weight and body mass index compared to the patients’ baseline status. Weight loss was also accompanied by improvements in metabolic parameters, suggesting an overall beneficial effect on the metabolic profile of women with PCOS. Overall, the study demonstrates that exenatide, when used in combination with metformin, may effectively contribute to weight reduction in this population group, reinforcing its therapeutic role in the management of obesity associated with PCOS [145]. In the double-blind, randomized, placebo-controlled trial by van Ruiten et al. (2022), strong evidence emerged that exenatide promotes weight reduction indirectly through central mechanisms, by modulating eating behavior [146].

Exenatide appears to exert beneficial effects on the female reproductive system, primarily by modulating the hormonal profile. Specifically, it has been associated with increased levels of FSH, which is essential for follicular development, as well as SHBG, which reduces circulating free androgen levels [77,141]. At the same time, exenatide administration has been linked to decreased serum testosterone concentrations—an important effect in women with PCOS, where hyperandrogenemia is a central pathophysiological feature [77,133]. These hormonal changes are associated with improvements in ovarian function, including increased ovulation frequency and restoration of menstrual cycle regularity [147,148]. Collectively, these findings suggest that exenatide may play a meaningful role in enhancing reproductive function in women with PCOS, likely through combined indirect mechanisms involving reduced insulin resistance and improved endocrine balance [77]. A randomized controlled trial by Renyuan Li et al. (2022) [149] further evaluated the effects of exenatide compared with metformin on fertility outcomes in overweight or obese women with PCOS. The study demonstrated that short-term preconception treatment with exenatide resulted in a significantly higher spontaneous pregnancy rate compared to metformin alone (29.2% vs. 14.7%), an effect likely driven by greater weight loss and improved insulin sensitivity. However, during longer follow-up, including the use of assisted reproductive technologies, no significant differences were observed in the overall pregnancy rates between the two groups. In addition, pregnancy outcomes, such as complications and delivery results, were comparable. In summary, exenatide appears to enhance the likelihood of natural conception in the short term, mainly through metabolic improvements, but does not seem to provide additional benefits in overall pregnancy rates or pregnancy outcomes when longer-term follow-up and assisted reproduction are considered [149]. Overall, exenatide plays a promising role in female reproductive health, particularly in women with PCOS, by improving metabolic status and modulating hormonal balance. It enhances ovulation and menstrual regularity and may increase spontaneous pregnancy rates. However, its long-term impact on overall fertility outcomes remains limited, indicating the need for further research. Additionally, most studies of GLP-1 receptor agonists are applied in women with PCOS, where GLP-1 agonists seem promising in various aspects. However, very few studies involved women without PCOS, with the findings being rather limited.

### 5.3. Tirzepatide

Tirzepatide is a novel therapeutic agent classified as a dual agonist of the glucagon-like peptide-1 (GLP-1) and glucose-dependent insulinotropic polypeptide (GIP) receptors, improving glycemic control in type 2 diabetes mellitus while also promoting significant weight reduction [12,14,150,151]. By concurrently stimulating both GLP-1 and GIP pathways, tirzepatide harnesses complementary and synergistic incretin effects that enhance the management of metabolic disorders [12,14,152]. These two hormones are members of the incretin system and are released from the intestinal tract in response to nutrient ingestion. GLP-1 primarily facilitates glucose-dependent insulin secretion and contributes to appetite suppression and satiety regulation, whereas GIP also promotes insulin secretion and is increasingly recognized for its involvement in lipid handling and energy homeostasis [12,14,153]. Consequently, dual incretin receptor activation is associated with improved glycemic outcomes, body weight reduction, and potential benefits in lipid metabolism [12,14]. Tirzepatide was approved by the U.S. Food and Drug Administration for the treatment of type 2 diabetes mellitus in 2022 [12]. The SURPASS-1 study conducted by Rosenstock et al. assessed the efficacy and safety of tirzepatide in individuals with type 2 diabetes mellitus who had insufficient glycemic control despite lifestyle interventions such as diet and physical activity alone. This study was designed as a randomized, double-blind, placebo-controlled phase 3 clinical trial. Participants were randomly allocated to receive once-weekly tirzepatide at doses of 5 mg, 10 mg, or 15 mg, for a total duration of 40 weeks. The main outcome measured was the change in glycated hemoglobin (HbA1c) from baseline, while additional endpoints included changes in body weight and the evaluation of safety and tolerability. The results showed that tirzepatide led to substantial and dose-dependent reductions in HbA1c, with many participants achieving near-normal glycemic levels. In addition, all treatment groups showed marked reductions in body weight, with losses ranging from approximately 7.0 to 9.5 kg, whereas the placebo group exhibited minimal change. A high proportion of patients reached target HbA1c levels (<7%), highlighting the drug’s strong glucose-lowering effect. In terms of safety, tirzepatide was generally well tolerated. The most common adverse events were gastrointestinal symptoms, such as nausea, diarrhea, and vomiting, which were mostly mild to moderate and tended to decrease over time, whilst the incidence of hypoglycemia was low. Overall, the study concluded that tirzepatide provides marked improvements in glycemic control and body weight with an acceptable safety profile, supporting its role as a promising new therapy for type 2 diabetes [142]. The SURPASS-2 trial conducted by Frías et al. compared the efficacy and safety of tirzepatide with once-weekly semaglutide in patients with type 2 diabetes mellitus inadequately controlled with metformin. This was a 40-week, randomized, open-label, phase 3 clinical trial involving multiple doses of tirzepatide (5 mg, 10 mg, and 15 mg) and semaglutide (1 mg). The primary endpoint was the change in HbA1c from baseline, with body weight reduction and safety outcomes assessed as key secondary endpoints. The results showed that tirzepatide was superior to semaglutide in reducing HbA1c across all dose levels. Additionally, tirzepatide led to significantly greater weight loss compared with semaglutide, with a clear dose-dependent effect. Regarding safety, both treatments had similar overall safety profiles. The most common adverse events were gastrointestinal (nausea, diarrhea, vomiting), which were generally mild to moderate. Hypoglycemia was uncommon in both groups. Serious adverse events occurred in approximately 5–7% of patients treated with tirzepatide, compared with about 3% among those receiving semaglutide. In summary, the study demonstrated that tirzepatide provided greater improvements in glycemic control and body weight reduction than semaglutide, supporting its potential as a more effective therapeutic option for type 2 diabetes mellitus management [154]. The post hoc analysis by Tchang et al. evaluated body weight reduction with tirzepatide in women across different reproductive stages, using data from the SURMOUNT clinical trial program. The analysis included women categorized by reproductive stage (premenopausal and postmenopausal) who received tirzepatide for obesity. The primary focus was to assess whether reproductive stage influenced the magnitude of weight loss achieved during treatment. Results showed that tirzepatide produced substantial and clinically meaningful weight reduction in women across all reproductive stages. Weight loss was consistent and dose-dependent, with higher doses associated with greater reductions in body weight. Importantly, the efficacy of tirzepatide appeared comparable between premenopausal and postmenopausal women, indicating that reproductive stage did not significantly affect treatment response. In general, the findings suggest that tirzepatide is effective for weight management in women regardless of reproductive stage, supporting its broad applicability in female patients with obesity [155].

The available data on the influence of tirzepatide on ovulation, menstrual regularity, conception rates, and fertility treatment outcomes remain limited. However, the significant weight loss and metabolic improvements observed in the SURMOUNT clinical trial program suggest a potentially meaningful impact on reproductive physiology [146,147]. Tirzepatide may contribute to improved ovarian function and fertility through several interrelated mechanisms. First, better glycemic control—driven by increased insulin sensitivity, lower fasting insulin concentrations, and reduced hyperinsulinemia—can mitigate ovarian androgen overproduction, which is a key feature of PCOS. Second, clinically meaningful weight reduction is associated with improvements in both metabolic status and reproductive outcomes in women affected by obesity-related endocrine dysfunction. Third, tirzepatide-induced metabolic changes may promote a less inflammatory internal environment by modulating adipokine secretion, including decreased leptin levels, increased adiponectin concentrations, and reductions in pro-inflammatory cytokines such as IL-1β, IL-6, and TNF-α [12,13,17]. Evidence regarding the use of tirzepatide during pregnancy and breastfeeding remains very limited. Current knowledge is primarily derived from animal studies and indirect data from other GLP-1 receptor agonists rather than controlled human trials. Preclinical findings suggest that exposure to GLP-1-based therapies during pregnancy may be associated with adverse fetal outcomes, including reduced fetal weight and potential developmental effects, particularly when exposure occurs during early gestation [156,157]. As a result, tirzepatide is not recommended during pregnancy, and discontinuation prior to conception is generally advised. Data on tirzepatide excretion into human breast milk and its effects on breastfed infants are currently insufficient. Limited pharmacokinetic evidence suggests that milk concentrations are negligible or undetectable; however, due to the absence of robust infant outcome data, most regulatory guidance recommends caution and individualized risk–benefit assessment during lactation [158].

Overall, tirzepatide represents a promising therapeutic breakthrough for obese women by targeting the dual pathways of GIP and GLP-1 receptors to drive profound weight loss and metabolic restoration. By mitigating insulin resistance, reducing systemic inflammation, and normalizing metabolic homeostasis, tirzepatide plays a pivotal role in restoring hypothalamic–pituitary–gonadal axis function. Clinical and real-world evidence increasingly suggests that these metabolic improvements can significantly enhance reproductive health outcomes, particularly by restoring ovulatory cycles and improving fertility in obese women suffering from conditions like Polycystic Ovary Syndrome (PCOS). However, given the mechanism of rapid weight loss and potential teratogenic risks, future clinical trials must prioritize evaluating its long-term safety, optimal washout periods before conception, and direct impact on obstetric outcomes to safely integrate tirzepatide into pre-pregnancy care pathways.

**Figure 8:**
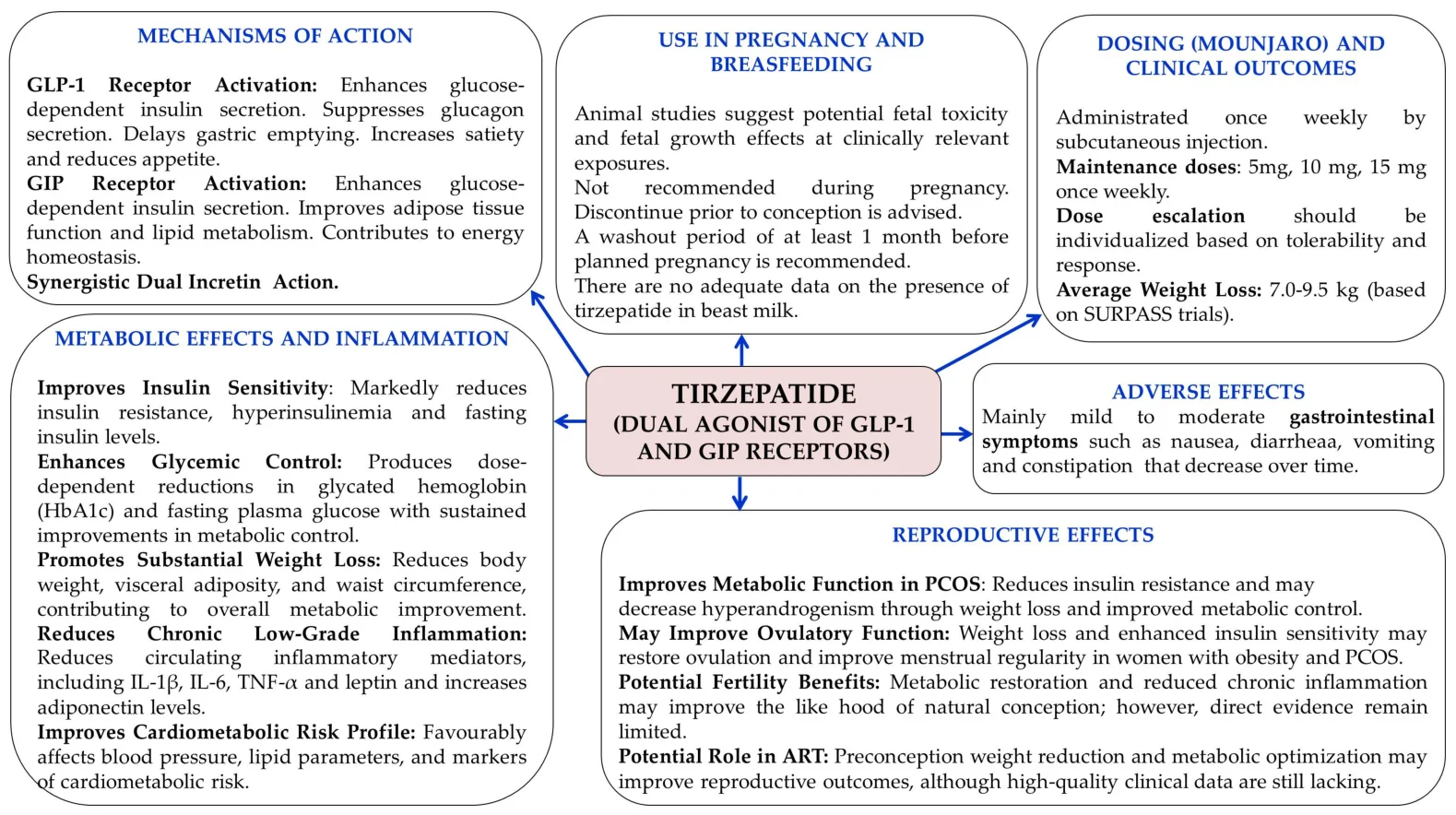
Overview of the mechanisms of action, metabolic and reproductive effects, dosing, safety profile, and use during pregnancy and breastfeeding of tirzepatide. Tirzepatide is a first-in-class dual glucose-dependent insulinotropic polypeptide (GIP) and glucagon-like peptide-1 (GLP-1) receptor agonist. By simultaneously activating both incretin pathways, it improves insulin sensitivity, glycemic control, and body weight while reducing obesity-associated inflammation. These metabolic benefits may contribute to improved reproductive function in women with obesity and polycystic ovary syndrome (PCOS). The figure also summarizes recommended maintenance dosing, common adverse effects, and current recommendations regarding pregnancy and breastfeeding (ART: assisted reproductive technology; GIP: glucose-dependent insulinotropic polypeptide; GLP-1: glucagon-like peptide-1; HbA1c: glycated hemoglobin; IL-1β, interleukin-1 beta; IL-6: interleukin-6; PCOS: polycystic ovary syndrome; TNF-α, tumor necrosis factor-alpha).

Figure 8 summarizes the principal pharmacological characteristics of tirzepatide. As a dual GIP/GLP-1 receptor agonist, tirzepatide exerts complementary actions on glucose homeostasis, appetite regulation, and energy metabolism, resulting in substantial and sustained weight loss together with marked improvements in glycemic control and insulin sensitivity. These metabolic improvements are accompanied by attenuation of obesity-associated inflammation and may indirectly enhance reproductive function, particularly in women with obesity and PCOS, although evidence for direct ovarian or endometrial effects remains limited. Figure 8 also highlights the recommended once-weekly dosing regimen, the predominantly gastrointestinal adverse-event profile, and the current recommendations to avoid tirzepatide during pregnancy and breastfeeding due to insufficient human safety data.

## 6. Integrating Mechanistic Knowledge into Personalized Therapy

The interplay between obesity and female reproductive dysfunction is recognized as one of the most complex challenges in the field of reproductive endocrinology. Traditionally, obesity management relied primarily on lifestyle modifications, although long-term adherence has generally been poor. The introduction of modern anti-obesity pharmacotherapy has substantially expanded therapeutic options by targeting the metabolic abnormalities that underlie reproductive dysfunction [159]. Importantly, current evidence suggests that improvements in fertility are mediated predominantly through systemic metabolic restoration rather than direct pharmacological effects on ovarian tissues [125]. This section presents an integrated framework for translating mechanistic knowledge into personalized reproductive care. It reviews the molecular and cellular mechanisms linking anti-obesity pharmacotherapy with ovarian and endometrial function, discusses phenotype-driven therapeutic selection, examines the emerging clinical complexities surrounding incretin-based therapies, and identifies critical gaps that will shape future research and clinical practice.

### 6.1. Molecular and Cellular Characterization of the Ovarian Microenvironment, Endometrial Receptivity, and Embryo Implantation

A mechanistic understanding of obesity-associated reproductive dysfunction requires integration of systemic metabolic disturbances with local ovarian and endometrial microenvironmental alterations. The metabolic abnormalities associated with obesity—including insulin resistance (IR), persistent low-grade inflammation, and altered lipid metabolism—collectively impair hypothalamic–pituitary–gonadal (HPG) axis function and contribute to ovarian dysfunction [5,55]. These systemic abnormalities are associated with altered gonadotropin dynamics, increased ovarian androgen production, and impaired follicular development [20,42], although the relative contribution of central versus peripheral mechanisms remains incompletely defined.

Metformin represents the most established pharmacological intervention targeting insulin resistance in this context. Through activation of AMPK and modulation of hepatic gluconeogenesis, metformin improves systemic insulin sensitivity and reduces circulating insulin concentrations [14,160]. In women with insulin-resistant reproductive phenotypes, such as PCOS, these metabolic effects are associated with reductions in androgen excess and improvements in ovulatory function [13,155,156]. These changes are thought to be mediated indirectly through normalization of insulin–theca cell interactions and downstream steroidogenic activity, including modulation of CYP17A1 expression [161,162,163].

Glucagon-like peptide-1 receptor agonists (GLP-1RAs), including semaglutide and liraglutide, improve reproductive outcomes primarily through systemic metabolic effects, including substantial weight loss, enhanced insulin sensitivity, and attenuation of chronic low-grade inflammation [12,164]. Although GLP-1-related signaling pathways have been identified in reproductive tissues, their physiological relevance within the human ovary and endometrium remains incompletely understood [165]. Accordingly, the beneficial reproductive effects observed in women with obesity are currently considered to result predominantly from restoration of metabolic homeostasis rather than from direct receptor-mediated actions within ovarian or endometrial tissues [165,166].

Obesity is characterized by profound alterations in the follicular microenvironment, including elevated concentrations of free fatty acids—particularly palmitic and stearic acid—in follicular fluid [167]. These lipotoxic changes promote endoplasmic reticulum stress, mitochondrial dysfunction, and oxidative damage within the oocyte, thereby compromising oocyte competence, early embryonic development, and overall reproductive efficiency [168,169]. By improving systemic metabolic status and reducing lipotoxicity and inflammation, GLP-1 receptor agonists may indirectly ameliorate these obesity-associated alterations, although convincing evidence for direct tissue-specific effects remains limited [170]. While inflammatory pathways such as NF-κB are increasingly recognized in the pathophysiology of obesity-related reproductive dysfunction [171], direct human evidence supporting GLP-1-mediated regulation of intra-follicular inflammatory cytokine signaling remains limited [165]. Accordingly, any potential protective effects on the ovarian [172] and endometrial microenvironments [173] are currently considered to be indirect and largely secondary to improvements in systemic metabolic homeostasis.

Dual GLP-1/GIP receptor agonists, such as tirzepatide, demonstrate greater metabolic efficacy than selective GLP-1 receptor agonists, producing superior weight loss and glycemic control while also influencing adipose tissue lipid handling through GIP receptor signaling, which may reduce circulating lipid substrates [12,174,175]. It remains uncertain whether these enhanced systemic metabolic effects confer measurable advantages at the level of ovarian or endometrial tissue, and this represents an ongoing area of active investigation [13].

Orlistat acts via a distinct gastrointestinal mechanism by inhibiting dietary fat absorption, thereby reducing postprandial lipid exposure and circulating triglyceride levels [12,71,176]. While this may contribute to improved systemic lipid profiles, its direct effects on ovarian physiology are not well established and are presumed to be indirect [177,178].

Centrally acting agents such as the combination of naltrexone and bupropion primarily modulate hypothalamic appetite and reward pathways [12]. Any reproductive benefits observed in patients treated with these agents are likely secondary to weight loss and improved systemic metabolic status rather than direct endocrine effects on the reproductive axis [179,180,181].

Collectively, current evidence suggests that anti-obesity pharmacotherapies may improve reproductive function primarily through systemic metabolic and inflammatory modulation rather than direct ovarian or endometrial targeting [8]. However, the precise contribution of local tissue-level mechanisms, particularly within the follicular and endometrial microenvironments, remains insufficiently characterized and warrants further mechanistic and clinical investigation [8]. Table 1 summarizes the principal molecular targets and mechanisms of action and their potential implications for ovarian function, endometrial receptivity, implantation, and female reproductive outcomes.

### 6.2. Phenotype-Based Personalization of Anti-Obesity Pharmacotherapy

The clinical heterogeneity of obesity-associated reproductive dysfunction underscores the limitations of a uniform “one-size-fits-all” therapeutic paradigm [182,183,184]. Patients present with diverse metabolic, behavioral, and reproductive characteristics that may differentially influence responsiveness to pharmacological interventions [182,183,184]. In this context, a phenotype-stratified framework may offer a useful conceptual approach to guide individualized therapy, although prospective validation remains necessary [182,183,184].

***Phenotype 1 (Metabolic/PCOS)*** represents the classical intersection of metabolic dysfunction and reproductive endocrine disturbance. These patients are characterized by severe insulin resistance (IR), clinical or biochemical hyperandrogenism, central adiposity, and associated clinical features such as acanthosis nigricans [185,186]. In this subgroup, therapeutic strategies primarily aim to improve insulin sensitivity and reduce hyperinsulinemia, thereby ameliorating downstream reproductive endocrine dysregulation [187]. Combination therapy with incretin-based agents (such as GLP-1 receptor agonists or dual GLP-1/GIP receptor agonists) and metformin has been proposed as a potentially complementary approach [188,189]. GLP-1-based therapies induce substantial weight loss and improve systemic insulin sensitivity, while metformin exerts additional effects on hepatic glucose production and peripheral insulin action [179,180]. Emerging evidence suggests that these metabolic improvements may be associated with normalization of ovulatory function and reductions in androgen excess, although direct ovarian effects remain incompletely defined [188].

***Phenotype 2 (Behavioral/Central Obesity)*** is characterized predominantly by dysregulated eating behavior, including emotional eating, binge eating tendencies, or hyperphagia, often accompanied by central adiposity and comparatively modest baseline metabolic impairment [190,191]. In such patients, interventions targeting central appetite regulation and reward circuitry may be particularly relevant. The combination of naltrexone and bupropion acts on hypothalamic melanocortin signaling while modulating mesolimbic reward pathways, thereby reducing hedonic eating behavior and caloric intake [12,190,191]. Although sustained weight loss has been associated with improvements in reproductive function in obese populations more broadly, direct evidence linking this pharmacological combination to restoration of ovulatory function remains limited [180,192]. Any reproductive benefits are therefore likely indirect, mediated through reductions in adiposity and systemic inflammatory burden [180].

***Phenotype 3 (Early Intervention Before ART)*** comprises women with class I obesity (BMI 30–34.9 kg/m^2^) and mild or intermittent ovulatory dysfunction who are being managed before the initiation of assisted reproductive technology (ART). In this population, early weight-loss interventions aim to optimize metabolic health, improve reproductive function, and reduce obesity-related pregnancy complications. Although weight reduction before ART improves metabolic parameters and may increase the likelihood of spontaneous conception, current meta-analyses indicate that pre-treatment weight loss does not significantly improve live birth rates following ART in women with obesity [193]. Nevertheless, achieving a 5–10% reduction in body weight before conception is recommended to improve overall health and reduce the risk of adverse maternal and pregnancy outcomes [193]. Short-acting GLP-1 receptor agonists, such as liraglutide, offer a favorable pharmacokinetic profile for short-term weight-reduction strategies. Their relatively rapid onset and reversibility may facilitate preconception weight optimization within a defined treatment window, followed by discontinuation prior to ovarian stimulation in accordance with current safety recommendations [194,195]. However, optimal timing of discontinuation and its impact on subsequent reproductive outcomes remain areas of ongoing investigation.

***Phenotype 4 (Gastrointestinal/Lipid-Driven)*** is characterized by pronounced dyslipidemia and dietary lipid excess or by intolerance or contraindications to incretin-based therapies [196]. In such patients, non-systemic approaches such as orlistat may represent an alternative therapeutic option. Orlistat reduces intestinal fat absorption, thereby lowering circulating triglyceride and free fatty acid levels [12]. While improvements in systemic lipid profiles may plausibly contribute to a more favorable metabolic environment for reproduction, direct effects on follicular physiology and oocyte quality remain largely theoretical and require further investigation.

Overall, this phenotype-based framework highlights the potential for aligning anti-obesity pharmacotherapy with dominant metabolic and behavioral characteristics in women of reproductive age. However, current evidence remains insufficient to support definitive therapeutic stratification. Future prospective studies are required to determine whether such phenotype-guided approaches translate into clinically meaningful improvements in ovulatory function, ART outcomes, and live birth rates. Table 2 summarizes the proposed phenotype-based pharmacological approach, highlighting the principal clinical characteristics, preferred therapeutic options, mechanisms of action, and expected reproductive benefits for women of reproductive age with obesity.

### 6.3. Navigating the Incretin Fertility Paradox

The integration of GLP-1 receptor agonists (GLP-1RAs) and dual GLP-1/GIP receptor agonists into reproductive medicine has introduced a clinically relevant tension between metabolic optimization and reproductive timing, herein conceptualized as the *incretin fertility paradox*. On one hand, these agents represent among the most effective non-surgical interventions for obesity, with robust evidence demonstrating substantial weight reduction and improvement in metabolic parameters, including insulin sensitivity [168,174]. In women with obesity-associated anovulation, particularly in the context of PCOS, such metabolic improvements have been associated with resumption of more regular ovulatory cycles in a proportion of patients, likely mediated through systemic reductions in IR and improvements in endocrine–metabolic homeostasis [8,186,197,198]. However, direct mechanistic effects on ovarian function and intra-follicular metabolic environments remain incompletely characterized [13,165,173]. On the other hand, the reproductive use of these agents is constrained by limited pregnancy safety data and signals of reproductive toxicity observed in preclinical studies. As a result, current regulatory guidance advises discontinuation prior to conception, with recommended washout periods varying by compound and formulation [199]. This creates a clinically relevant temporal mismatch between metabolic benefit and reproductive intent, particularly in patients pursuing time-sensitive fertility treatments. During the discontinuation phase, clinical studies have demonstrated a tendency toward weight regain and partial reversal of metabolic improvements, which may adversely affect reproductive parameters in susceptible individuals [200,201]. Nevertheless, the extent to which such metabolic fluctuations directly influence oocyte competence and endometrial receptivity in humans remains an active area of investigation [202].

To address this challenge, a structured clinical approach has been proposed, encompassing sequential phases of metabolic optimization, treatment discontinuation, conception planning, and early gestational care (Table 3).

During ***Phase 1 (Metabolic and Weight Optimization)****,* pharmacological therapy is typically combined with lifestyle interventions and appropriate contraceptive measures, in line with current clinical recommendations for reproductive-age women receiving anti-obesity pharmacotherapy. Oral contraceptives are unreliable in this phase due to the delayed gastric emptying induced by incretins, which compromises the pharmacokinetics and absorption of oral steroidal hormones [54,203,204].

In ***Phase 2 (The Mandated Washout Period)****,* anti-obesity pharmacotherapy is discontinued [205], and patients are transitioned to pregnancy-compatible metabolic strategies, including low-to-moderate-dose metformin where clinically appropriate, combined with structured dietary and behavioral interventions designed to mitigate weight regain [186].

***Phase 3 (The Active Conception Window)*** represents a period of dynamic metabolic readjustment, during which ovulatory function may improve in parallel with changes in weight and insulin sensitivity. However, robust data describing patterns such as ovulatory variability following treatment cessation are currently lacking, and claims regarding systematic reproductive “rebound” phenomena remain speculative [102].

Finally, ***Phase 4 (Gestational Surveillance)*** management focuses on minimizing metabolic risk through non-teratogenic strategies, including lifestyle modification and selected pharmacological agents with established safety profiles, in order to reduce the risk of gestational metabolic complications such as gestational diabetes and hypertensive disorders [205].

**Table 3:** Clinical management timeline of the GLP-1 fertility paradox.

Phase	Duration/Timing	Physiological and Therapeutic Goals	Teratogenic Risks and Safety Concerns	Clinical Strategies and Action Plan
Phase 1: Metabolic and Weight Optimization	Approximately 3–6 months or individualized according to weight-loss goals.	Achieve clinically meaningful weight loss (≥5–10%), improve insulin sensitivity, reduce hyperandrogenism, and restore ovulatory function [168,186].	GLP-1 receptor agonists are contraindicated during pregnancy. Effective contraception should be used during treatment. Tirzepatide may reduce oral contraceptive absorption during dose escalation because of delayed gastric emptying [54,203,204].	Initiate GLP-1 receptor agonist therapy when indicated; provide structured lifestyle intervention; ensure reliable contraception; monitor weight, menstrual regularity and ovulatory function.
Phase 2: Washout Before Conception	Follow product-specific recommendations; for semaglutide, discontinue at least 2 months before planned conception.	Allow adequate drug elimination before conception [205,206] while maintaining metabolic improvements through lifestyle interventions [194].	Weight regain and deterioration of insulin resistance (IR) may occur after treatment discontinuation [200,201].	Discontinue GLP-1-based therapy before conception; continue nutritional counseling and physical activity; metformin may be continued in women with PCOS [186].
Phase 3: The Active Conception Window	After completion of the recommended washout period until spontaneous conception or ART.	Preserve metabolic benefits while optimizing ovulation, endometrial function and embryo development [194].	Anti-obesity medications remain contraindicated throughout conception attempts and pregnancy. Evidence for “ovulatory rebound” or increased multiple pregnancy risk is currently lacking.	Confirm discontinuation of GLP-1 therapy before ovarian stimulation or conception; optimize nutrition, physical activity and glycemic control; continue pregnancy-compatible therapies when indicated.
Phase 4: Gestational Surveillance	Throughout pregnancy.	Maintain maternal metabolic health and minimize obesity-related obstetric complications [205].	Anti-obesity medications should not be restarted during pregnancy. Obesity increases the risk of gestational diabetes, hypertensive disorders and cesarean delivery [206].	Manage weight through nutrition, physical activity and routine obstetric care; insulin remains the preferred treatment when pharmacologic glycemic control is required during pregnancy [207].

(ART, assisted reproductive technology).

### 6.4. Current Knowledge Gaps and Future Research Directions

As anti-obesity medications become more widely incorporated into reproductive medicine, important gaps in both mechanistic knowledge and clinical evidence continue to limit their evidence-based application. Although existing research has demonstrated promising therapeutic potential, several unresolved questions remain regarding their biological effects, long-term safety, and optimal clinical use in reproductive populations. Instead of making broad recommendations for additional investigation, this section highlights three major areas of uncertainty that are expected to shape future translational and clinical research in this evolving field. (Table 4).

The first major gap concerns transgenerational epigenomic effects. Maternal obesity is strongly associated with persistent epigenetic alterations in the oocyte, including changes in DNA methylation and histone modifications, which have been implicated in the intergenerational transmission of metabolic and cardiovascular risk [208,209]. Although anti-obesity pharmacotherapies, particularly GLP-1 receptor agonists, effectively normalize systemic metabolic parameters, their impact on the oocyte microenvironment remains insufficiently characterized [210]. It remains unclear whether the rapid mobilization of adipose tissue during intensive weight loss transiently modifies the circulating lipid and one-carbon metabolite milieu reaching the follicular microenvironment, thereby influencing peroxisomal phospholipid metabolism and methyl donor availability. In light of recent evidence demonstrating that maternal obesity disrupts epigenetic reprogramming through peroxisome-dependent phospholipid–methyl uncoupling during zygotic genome activation, an important unanswered question is whether the metabolic remodeling induced by anti-obesity therapies similarly affects epigenetic programming in the oocyte and early embryo, either transiently or persistently [178]. Conversely, it is equally plausible that sustained metabolic normalization may partially reverse obesity-associated epigenetic signatures [211]. Addressing this uncertainty will require prospective longitudinal cohort studies incorporating follicular fluid multi-omics profiling (including cfDNA, transcriptomics, and metabolomics) alongside long-term follow-up of offspring health outcomes [212].

The second gap relates to the optimization of assisted reproductive technology (ART) outcomes in the context of anti-obesity pharmacotherapy. At present, there is no consensus regarding the optimal timing for discontinuation of anti-obesity medications prior to ovarian stimulation or embryo transfer [186,194]. Clinical decision-making requires careful balancing of competing risks, as extended pharmacotherapy may postpone fertility treatment in women with reduced ovarian reserve [8,197], while early discontinuation can lead to rapid weight regain and metabolic destabilization during the IVF cycle [197]. There is a pressing need for well-designed randomized controlled trials comparing different discontinuation strategies, including early cessation following clinically meaningful weight loss versus continuation until the recommended preconception washout period. Key reproductive endpoints should include oocyte yield, blastocyst quality, implantation rates, and live birth outcomes.

The third gap involves the comparative reproductive effects of single versus dual incretin receptor agonism. While dual GLP-1/GIP receptor agonists such as tirzepatide demonstrate superior weight loss and glycemic control compared with selective GLP-1 receptor agonists, it remains uncertain whether these macro-metabolic advantages translate into differential reproductive benefits [154]. Emerging experimental data suggest that GLP-1 receptors have been identified in several reproductive tissues, including the endometrium, while incretin receptors are also expressed in vascular endothelium [213]. Although direct human evidence remains limited, these findings raise the possibility that incretin signaling may influence endometrial perfusion, receptivity, and decidualization. However, whether dual agonism confers specific advantages in terms of endometrial receptivity, immune modulation at the maternal–fetal interface, or implantation success remains speculative. Resolving this question will require head-to-head mechanistic and clinical studies incorporating endometrial transcriptomic profiling, implantation biomarkers, and cycle-specific reproductive outcomes during the window of implantation.

**Table 4:** Research gaps and emerging priorities in anti-obesity pharmacotherapy and female fertility.

Major Research Area	Specific Unresolved Research Question	Current Knowledge Gap	Proposed Methodological Approach/Study Design	Potential Clinical and Translational Impact
Transgenerational Epigenomics	Does rapid weight loss via GLP-1 receptor agonists affect the epigenetic profile of the oocyte and the long-term health of the offspring?	Although metabolic improvement is beneficial, the effects of GLP-1RA-induced weight loss on oocyte DNA methylation, histone modifications and embryonic developmental programming remain largely unexplored [178,208,209,210].	Prospective longitudinal cohorts evaluating follicular-fluid biomarkers, granulosa-cell transcriptomics, and long-term follow-up of offspring after maternal preconception GLP-1RA exposure.	Clarifying whether pharmacological metabolic optimization before conception improves reproductive outcomes without adversely affecting developmental programming.
ART Outcome Optimization	What is the ideal BMI target and optimal kinetic threshold for discontinuing anti-obesity medications before starting an in vitro fertilization (IVF) cycle?	Current reproductive and obesity guidelines provide no evidence-based recommendations regarding BMI targets, percentage weight loss, or optimal discontinuation interval before ovarian stimulation [186,194].	Multicenter prospective studies and randomized controlled trials comparing different discontinuation strategies before IVF treatment.	Development of standardized preconception protocols aimed at maximizing oocyte competence, embryo quality and live birth while minimizing unnecessary treatment interruption.
Incretin Comparative Superiority	Does tirzepatide (dual GLP-1/GIP receptor agonist) outperform semaglutide (selective GLP-1RA) in directly improving endometrial receptivity?	Whether tirzepatide exerts direct ovarian or endometrial effects independent of its profound metabolic and weight-loss effects remains unknown, as mechanistic studies evaluating its actions on reproductive tissues are currently lacking [8].	Head-to-head mechanistic clinical trials including endometrial transcriptomics (RNA-seq), follicular-fluid metabolomics and reproductive outcomes.	Identification of drug-specific reproductive effects that could support precision medicine approaches for women with obesity undergoing fertility treatment.

(ART, assisted reproductive technology; BMI, body mass index; DNA, deoxyribonucleic acid; GIP, glucose-dependent insulinotropic polypeptide; GLP-1, glucagon-like peptide-1; GLP-1RA, glucagon-like peptide-1 receptor agonist; IVF, in vitro fertilization; RNA-seq, RNA sequencing;).

## 7. Conclusions and Clinical Implications

Obesity is a chronic, multifactorial metabolic disease with profound implications for female reproductive health. The evidence synthesized in this review highlights that adiposity is not merely a condition of energy imbalance but a complex endocrine and inflammatory disorder that disrupts multiple levels of the HPG axis. In women of reproductive age, obesity is strongly associated with menstrual irregularities, anovulation, subfertility, and adverse pregnancy outcomes. These effects are particularly pronounced in conditions such as PCOS, where insulin resistance, hyperinsulinemia, and hyperandrogenism create a self-perpetuating cycle of metabolic and reproductive dysfunction.

A central conclusion emerging from the reviewed literature is that pharmacological weight-loss interventions may play an increasingly important role not only in the management of obesity itself but also in the restoration of reproductive function. Anti-obesity medications exert their effects through diverse mechanisms, including modulation of appetite, insulin sensitivity, energy expenditure, and gastrointestinal motility. Importantly, many of these mechanisms intersect directly or indirectly with reproductive endocrine pathways, suggesting that improvements in metabolic health may translate into clinically meaningful benefits in fertility and menstrual function.

Among the most established pharmacological agents, metformin has long been used in the management of type 2 diabetes mellitus and has been widely adopted in reproductive endocrinology, particularly in PCOS. Its primary mechanisms—reduction in hepatic gluconeogenesis, enhancement of peripheral insulin sensitivity, and activation of AMPK—result in improved glycemic control and modest weight reduction. Beyond its metabolic effects, metformin has demonstrated beneficial actions on ovarian function, including improved ovulation rates, restoration of menstrual cyclicity, and partial reduction in androgen levels. However, its impact on clinically definitive reproductive outcomes such as live birth rates remains inconsistent across studies. Some evidence suggests improved pregnancy rates, particularly in insulin-resistant or obese women, while other data highlight limited or no superiority compared to alternative ovulation induction strategies. Furthermore, concerns regarding potential adverse effects during pregnancy, although not conclusively established, underscore the need for careful patient selection and monitoring.

GLP-1 receptor agonists, such as liraglutide, semaglutide and exenatide, represent a newer and rapidly expanding class of anti-obesity agents with significant metabolic and potential reproductive benefits. These drugs enhance glucose-dependent insulin secretion, suppress glucagon release, delay gastric emptying, and reduce appetite through central nervous system pathways. Collectively, these mechanisms lead to clinically meaningful weight loss and improved glycemic control. Importantly, in women with PCOS, GLP-1 receptor agonists have been associated with improved hormonal profiles, including reductions in serum testosterone, increases in SHBG, and improved follicular development. These changes translate into improved ovulatory function and menstrual regularity. Some studies also suggest enhanced spontaneous pregnancy rates following short-term preconception treatment, although long-term fertility outcomes remain less clear. The overall evidence indicates that GLP-1 receptor agonists may offer dual benefits in metabolic and reproductive domains, particularly in obese women with PCOS, but their reproductive indications remain indirect and not formally established.

The emergence of tirzepatide, a dual GLP-1/GIP receptor agonist, represents a significant advancement in the pharmacotherapy of obesity. The dual incretin mechanism provides synergistic effects on insulin secretion, appetite regulation, and lipid metabolism, resulting in substantial improvements in metabolic health. Although direct evidence regarding reproductive outcomes is still limited, the metabolic improvements observed—particularly reductions in insulin resistance, systemic inflammation, and adipokine dysregulation—suggest a biologically plausible beneficial effect on ovarian function. In women with obesity-related reproductive dysfunction, particularly PCOS, tirzepatide may therefore hold future therapeutic potential, although this remains speculative at present.

A key overarching finding from this review is that improvements in reproductive function following anti-obesity pharmacotherapy are likely mediated primarily through metabolic normalization rather than direct gonadal effects. Weight loss leads to decreased insulin resistance and hyperinsulinemia, which in turn reduces ovarian androgen production. Additionally, reductions in adipose-derived inflammatory cytokines such as interleukin-6 (IL-6), tumor necrosis factor-alpha (TNF-α), and leptin, along with increases in adiponectin, contribute to a more favorable endocrine environment for ovulation and implantation. These interconnected pathways emphasize the central role of metabolic health in reproductive physiology.

However, it must be stressed that direct clinical evidence in women remains limited, and many of the proposed reproductive benefits are derived from small clinical studies, indirect metabolic effects, or preclinical data. Therefore, these expected outcomes should be interpreted with caution until confirmed by large, well-designed randomized clinical trials with reproductive endpoints. Tirzepatide achieves unprecedented weight loss, but case reports suggest potential sexual side effects, whilst naltrexone/bupropion may benefit sexual desire through mood improvement, and phentermine/topiramate primarily enhances psychological well-being [214]. Furthermore, due to other causes, some patients are prone to stopping the therapy for various reasons, and health disparities may arise [215]. Across all drug classes, sexual endpoints were typically secondary outcomes in clinical trials rendering the export of safe conclusion arduous, whilst many other factors such as age may be important in personalizing the therapeutic regimens, since such medications have shown to offer significant benefits but with particular safety considerations for each age group [216]. To these factors affecting the effect of anti-obesity treatments should also be added the compliance of the patients, which, due to high costs (as is the case with GLP-1 and GIP agonists) or side effects, results in real-world results that do not align with those of the clinical trials [217,218].

Thus, several limitations in the current evidence base must be acknowledged. First, many studies are short-term and focus primarily on surrogate outcomes such as weight loss, hormonal changes, or ovulation rates, rather than definitive reproductive endpoints like live birth rates or long-term fertility outcomes. Second, heterogeneity in study design, patient populations, and intervention protocols limits the comparability of results across trials. Third, most data derive from populations with PCOS or metabolic syndrome, and therefore may not be generalizable to all women with obesity-related reproductive dysfunction. Finally, long-term safety data, particularly in relation to pregnancy and lactation, remains insufficient for newer agents such as GLP-1 receptor agonists and dual incretin therapies. Future perspectives in this field should focus on several key directions. There is a critical need for large-scale, long-term randomized controlled trials evaluating the direct effects of anti-obesity medications on fertility outcomes, including ovulation rates, time to conception, pregnancy maintenance, and live birth rates. Such studies should also stratify participants based on metabolic phenotype, degree of insulin resistance, and presence or absence of PCOS, in order to identify subgroups most likely to benefit from specific therapies. Also, mechanistic studies are required to further elucidate the pathways linking pharmacologically induced weight loss to reproductive recovery. While insulin and androgen modulation are well established, the roles of gut hormones, adipokines, mitochondrial function, and hypothalamic regulation remain incompletely understood. In particular, the emerging concept of the gut–brain–ovary axis may provide a novel framework for understanding how metabolic interventions influence reproductive endocrinology. In addition, the safety of anti-obesity medications in the context of conception, pregnancy, and lactation must be rigorously evaluated. Given the increasing use of GLP-1 receptor agonists and dual incretin therapies in women of reproductive age, clear guidelines are needed regarding treatment discontinuation prior to conception and risk assessment during early pregnancy. Current evidence suggests potential risks based on animal studies and limited human data, reinforcing the need for pharmacovigilance and prospective registries. Moreover, personalized medicine approaches should be integrated into future clinical practice. Not all women with obesity-related reproductive dysfunction respond similarly to pharmacological interventions. Factors such as baseline BMI, insulin resistance severity, androgen levels, and ovarian reserve may influence treatment response. Therefore, individualized therapeutic strategies combining pharmacological agents with lifestyle interventions, insulin sensitizers, or ovulation induction agents may offer the most effective outcomes. Finally, the concept of off-label use of anti-obesity medications in reproductive medicine requires careful ethical and clinical consideration. While emerging evidence suggests potential benefits, particularly in PCOS-related infertility, such use should be guided by robust clinical evidence and multidisciplinary decision-making involving endocrinologists, reproductive specialists, and primary care providers.

The comparative evidence summarized in Table 5 highlights the considerable heterogeneity among currently available anti-obesity pharmacotherapies with respect to reproductive outcomes. While GLP-1 receptor agonists, particularly liraglutide, semaglutide, and exenatide, consistently demonstrate favorable effects on weight reduction, insulin sensitivity, menstrual regularity, ovulation, and, in selected studies, pregnancy outcomes, the overall evidence remains uneven across drug classes. For several agents, reproductive benefits are supported primarily by improvements in metabolic health rather than by robust fertility-specific endpoints, emphasizing the need for adequately powered randomized trials evaluating live birth, reproductive safety, and long-term maternal and offspring outcomes.

In conclusion, anti-obesity pharmacotherapy represents a promising and rapidly evolving therapeutic approach with important implications for female reproductive health. Beyond achieving clinically meaningful weight loss, several FDA-approved and off-label agents, including metformin, exenatide, GLP-1 receptor agonists, and tirzepatide, improve insulin sensitivity, reduce hyperandrogenism, restore menstrual regularity, and promote ovulation, particularly in women with obesity and polycystic ovary syndrome. These metabolic and endocrine benefits support a shift from weight management alone toward a more integrated strategy of metabolic and reproductive rehabilitation. However, current evidence remains insufficient to establish anti-obesity medications as primary fertility treatments, and important uncertainties persist regarding reproductive safety, optimal preconception discontinuation, assisted reproductive technology outcomes, and long-term maternal and offspring health. Therefore, treatment should be individualized according to metabolic phenotype, reproductive goals, and pregnancy planning. Future large, well-designed randomized clinical trials integrating mechanistic biomarkers with fertility outcomes are essential to define the optimal role of these therapies within precision reproductive medicine.

**Table 5:** Evidence from clinical trials of anti-obesity pharmacotherapies and their effects on female reproductive outcomes.

Drug/Class	Study	Study Design	Sample Size	Population	Drug and Dose	Duration	Weight-Loss Outcomes	Reproductive Outcomes	Major Limitations
Orlistat	XENDOS (Torgerson et al., 2004) [69]	RCT	3305	Adults with obesity	Orlistat 120 mg TID	4 years	Mean weight loss 2.4%; improved insulin sensitivity and cardiometabolic profile	Reproductive outcomes not evaluated.	Not fertility-focused; mixed-sex population
	Vosnakis et al., 2013 [70]	Prospective study	61 PCOS and 20 controls	Obese women with PCOS	Orlistat 120 mg TID	24 weeks	Significant weight and BMI reduction	↑ SHBG, ↑ LH, ↓ testosterone, ↑ AMH.	Small sample; no pregnancy endpoint
	Kumar et al., 2014 [71]	RCT	90	Women with PCOS	Orlistat 120 mg BID	3 months	~7.8 kg weight loss	Pregnancy rate 40%; ovulation 33.3%.	Short follow-up
	Tong et al., 2022 [72]	Controlled study	58	Women undergoing embryo transfer	Orlistat	IVF cycles	Not primary endpoint	Higher clinical pregnancy; no live birth benefit.	Small IVF cohort
	Wang et al., 2021 [74]	Double-blind RCT	877	Overweight/obese infertile women before IVF	Orlistat	4–12 weeks	Greater weight loss than placebo	No improvement in live birth or pregnancy rates.	Short intervention
	FIT-PLESE (Legro et al., 2022) [75]	RCT	379	Women with obesity and unexplained infertility	Lifestyle and orlistat	Preconception	Greater weight loss	No increase in live birth rate.	Multifactorial intervention
Liraglutide (GLP-1 RA)	SCALE (Pi-Sunyer et al., 2015) [79]	RCT	3731	Adults with obesity	Liraglutide 3.0 mg/day	56 weeks	63% lost ≥5%; 33% lost ≥10% body weight	Reproductive outcomes not evaluated.	General obesity trial
	Nylander et al., 2017 [80]	Double-blind RCT	72	Overweight women with PCOS	Liraglutide 1.8 mg/day	26 weeks	Mean weight loss 5.2 kg	Improved menstrual regularity, ↓ ovarian volume, ↑ SHBG, ↓ testosterone.	Small study
	Salamun et al., 2018 [81]	Open-label RCT	28	Infertile obese women with PCOS	Metformin ± liraglutide 1.2 mg/day	12 weeks	Similar weight loss in both groups	Pregnancy per embryo transfer 85.7% vs. 28.6%; higher cumulative pregnancy rate.	Small sample; combination therapy.
Semaglutide (GLP-1 RA)	STEP 1 (Wilding et al., 2021) [164]	RCT	1961	Adults with obesity or overweight without diabetes	Semaglutide 2.4 mg once weekly	68 weeks	Approximately 15% body weight reduction	Reproductive outcomes not assessed.	Obesity trial; fertility endpoints not evaluated.
	OASIS 1 (Knop et al., 2023) [89]	RCT	667	Adults with obesity or overweight	Oral semaglutide 50 mg daily	68 weeks	Mean weight loss 15.1%	Reproductive outcomes not assessed.	General obesity population
	Carmina and Longo, 2023 [93]	Prospective observational study unresponsive to lifestyle intervention	27	Obese women with PCOS	Semaglutide 0.5 mg weekly	6 months	Significant weight reduction (approximately 11.5 kg)	Normalization of menstrual cycles in most responders.	Small sample size; no control group
	Chen et al., 2025 [94]	RCT	120	Overweight/obese PCOS	Semaglutide 1 mg weekly + metformin 1000 mg BID	16 weeks	Greater weight loss than metformin alone	Improved menstrual regularity, ovulatory function and higher natural pregnancy rate (35% vs. 15%).	Single-center study; relatively short follow-up
Phentermine/Topiramate	Khera et al. 2016 [100]	Systematic review & meta-analysis of RCTs	29,018	Adults with obesity	Standard approved doses	~1 year	Greatest weight loss (~8.8 kg) among approved medications	No direct fertility outcomes.	No reproductive endpoints; pregnancy contraindicated
Naltrexone/Bupropion	Greenway et al. (COR-I), 2010 [180]	RCT	1742	Adults with obesity or overweight with comorbidities	Naltrexone SR 32 mg and bupropion SR 360 mg daily	56 weeks	Mean weight loss 6.1% vs. 1.3% with placebo	No reproductive outcomes evaluated.	Not designed to assess fertility
	Apovian et al. (COR-II), 2013 [108]	RCT	1496	Adults with obesity or overweight	Naltrexone SR 32 mg and bupropion SR 360 mg daily	56 weeks	Significant weight loss compared with placebo	No reproductive or hormonal outcomes reported.	General obesity population
Metformin	Legro et al., 2007 [219]	RCT	626	Infertile women with PCOS	Metformin 1000 mg twice daily	~6 months	Modest weight reduction	Improved ovulation but lower live birth rate than clomiphene; combination therapy most effective.	Weight loss modest
Exenatide (GLP-1 RA)	Elkind-Hirsch et al., 2008 [220]	RCT	60	Women with obesity and PCOS	Exenatide 10 μg BID vs. metformin	24 weeks	Greater weight loss than metformin	Improved menstrual cyclicity and ovulation.	Small sample size
	Ye et al., 2023 [148].	Systematic review and meta-analysis	9 RCTs (785 women)	Overweight/obese women with PCOS	Exenatide 5–10 μg twice daily	12–24 weeks	Exenatide significantly reduced body weight and BMI compared with metformin	Exenatide was associated with higher spontaneous pregnancy rates, improved ovulation rates and improved menstrual regularity compared with metformin.	Based mainly on relatively small RCTs
Tirzepatide (Dual GIP/GLP-1 Receptor Agonist)	SURMOUNT-1 (Jastreboff et al., 2022) [174]	RCT	2539	Adults with obesity or overweight without diabetes	Tirzepatide 5, 10 or 15 mg once weekly	72 weeks	Mean weight loss 15–21% depending on dose	Reproductive outcomes not assessed.	Fertility not evaluated
	SURMOUNT-3 (Wadden et al., 2023) [221]	RCT	806	Adults with obesity following intensive lifestyle intervention	Tirzepatide 10–15 mg weekly	72 weeks	Additional mean weight loss of 18.4% after lifestyle intervention	No reproductive outcomes assessed.	Obesity study only
	SURMOUNT-4 (Aronne et al., 2024) [222].	RCT	783	Adults with obesity or overweight	Tirzepatide 10–15 mg weekly	88 weeks	Sustained weight loss; withdrawal resulted in weight regain	Reproductive outcomes not evaluated.	No fertility-specific endpoints

(AMH, anti-Müllerian hormone; BID, twice daily; BMI, body mass index; GLP-1 RA, glucagon-like peptide-1 receptor agonist; GIP, glucose-dependent insulinotropic polypeptide; IVF, in vitro fertilization; LH, luteinizing hormone; PCOS, polycystic ovary syndrome; RCT, randomized controlled trial; SHBG, sex hormone-binding globulin; SR, sustained release; TID, three times daily).

## Figures and Tables

**Table 1:** Target mechanism of anti-obesity drugs on the ovarian microenvironment and endometrial receptivity and embryo implantation.

Drug Class/Agent	Primary Molecular Targets	Effects on Follicular Fluid and Oocyte Quality	Impact on Granulosa and Theca Cells	Endometrial Receptivity and Embryo Implantation
Metformin (Biguanide)	AMPK activation; inhibition of mitochondrial complex I [14].	May indirectly improve the follicular microenvironment by reducing hyperinsulinemia, oxidative stress, and metabolic dysfunction associated with obesity and insulin resistance [161,162,163].	Reduces ovarian androgen production by normalizing insulin–theca cell interactions and modulating steroidogenesis, including CYP17A1 expression, thereby improving ovulatory function [14,16,111,112,161,162,163].	May indirectly improve endometrial function through restoration of systemic metabolic homeostasis and insulin sensitivity [111,118,163]; evidence for direct effects on implantation remains limited [123,163].
GLP-1RAs (Semaglutide, Liraglutide, Exenatide)	GLP-1 receptor agonism; systemic improvement of insulin sensitivity, body weight and inflammatory status [170,171,172,173].	May indirectly reduce follicular lipotoxicity, oxidative stress and mitochondrial dysfunction through metabolic improvement [167,168,169]; evidence for direct ovarian effects is limited [170].	May indirectly improve follicular function by reducing hyperinsulinemia and systemic inflammation [171]; direct regulation of granulosa-cell apoptosis or intra-follicular cytokine signaling in humans remains unconfirmed [165].	May indirectly enhance endometrial receptivity through improved metabolic homeostasis and reduced systemic inflammation; direct endometrial effects remain insufficiently established [173].
Dual GLP-1/GIP Agonists (Tirzepatide)	Synergistic GLP-1 and GIP receptor activation; enhancement of systemic metabolic regulation and adipose tissue lipid handling [13,174,175].	May indirectly improve the follicular microenvironment by reducing lipotoxicity and metabolic dysfunction secondary to marked weight loss and improved glycemic control [174,175]; direct ovarian effects remain unconfirmed [13].	Improves insulin resistance and may indirectly reduce ovarian hyperandrogenism through systemic metabolic improvement [174,175]; direct effects on granulosa or theca cells have not been established [13].	Whether the enhanced metabolic effects of tirzepatide translate into improved endometrial receptivity or implantation remains uncertain, and direct reproductive evidence is currently lacking [13].
Orlistat (Gastric & Pancreatic Lipase Inhibitor)	Reversible inhibition of gastrointestinal lipases, reducing dietary fat absorption, postprandial lipid exposure, and circulating triglyceride levels [67].	May indirectly improve the ovarian metabolic environment by reducing systemic lipid exposure; direct effects on ovarian physiology remain unestablished [177,178].	Νo established direct cellular mechanisms [177,178].	Indirect improvement through metabolic control and weight reduction; no direct evidence for implantation effects [179].
Naltrexone/Bupropion	μ-opioid receptor antagonism; POMC neuron activation [12].	Indirect improvement through weight loss [12,180]; no demonstrated direct ovarian effects [181].	No convincing evidence of direct effects on granulosa or theca cells [181].	No direct evidence regarding endometrial receptivity; benefits are presumed secondary to weight reduction [68].

(AMPK, adenosine monophosphate-activated protein kinase; CYP17A1, cytochrome P450 family 17 subfamily A member 1; GIP, glucose-dependent insulinotropic polypeptide; GLP-1, glucagon-like peptide-1; GLP-1RA, glucagon-like peptide-1 receptor agonist).

**Table 2:** Phenotype-based pharmacological approach for reproductive-aged women with obesity.

Patient Phenotype	Clinical and Metabolic Characteristics	Primary Mechanism of Reproductive Dysfunction	Proposed Pharmacotherapy	Clinical Rationale and Therapeutic Goals
Phenotype 1 (Metabolic/PCOS)	PCOS; severe insulin resistance; clinical or biochemical hyperandrogenism; central adiposity; acanthosis nigricans [185,186].	Severe insulin resistance and hyperinsulinemia reduce hepatic SHBG synthesis, increase ovarian androgen production, and contribute to chronic anovulation [187].	GLP-1 receptor agonist (e.g., semaglutide) or dual GLP-1/GIP receptor agonist (e.g., tirzepatide) + metformin *(off-label combination)* [188,189].	Maximizes insulin sensitization and promotes weight loss, reduces hyperandrogenism, and may improve ovulatory function, although its direct effects on the ovary remain incompletely understood [163,186,190].
Phenotype 2 (Behavioral/Central Obesity)	Central adiposity; binge or emotional eating; mild metabolic impairment [191].	Chronic low-grade inflammation and neuroendocrine dysregulation affecting the hypothalamic–pituitary–ovarian axis [192].	Naltrexone/bupropion [191].	Improves appetite regulation and food craving; reproductive benefits are expected to occur indirectly through sustained weight loss [180].
Phenotype 3 (Early Intervention before ART)	BMI 30–35 kg/m^2^; intermittent anovulation; planned IVF/ART cycles [193].	Obesity-associated impairment of oocyte competence and endometrial function [194].	Liraglutide [195].	Facilitates preconception weight reduction before ART. Treatment should be discontinued before attempting conception [195].
Phenotype 4 (Lipid-Driven Obesity)	Dyslipidemia; high dietary fat intake; contraindications or intolerance to GLP-1RAs [196]	Lipotoxicity and oxidative stress within the ovarian microenvironment [197].	Orlistat [196].	Reduces dietary fat absorption and promotes weight loss, potentially decreasing systemic lipotoxicity [196]; evidence for direct ovarian effects remains limited.

(ART, assisted reproductive technology; BMI, body mass index; GLP-1RAs, glucagon-like peptide-1 receptor agonists; IVF, in vitro fertilization; PCOS, polycystic ovary syndrome; SHBG, sex hormone-binding globulin).

## Data Availability

No new data were created in this study.

## References

[1] Varra F.N., Varras M., Varra V.K., Theodosis-Nobelos P. (2024). Molecular and pathophysiological relationship between obesity and chronic inflammation in the manifestation of metabolic dysfunctions and their inflammation-mediating treatment options (Review). Mol. Med. Rep..

[2] Varra F.N., Theodosis-Nobelos P., Varra V.K., Varras M. (2025). Mechanistic Insights into Antioxidant Interventions Targeting Obesity-Induced Oxidative Stress in the Pathogenesis and Complications of Type 2 Diabetes Mellitus. Curr. Issues Mol. Biol..

[3] Varra F.N., Varras M., Varra V.K., Theodosis-Nobelos P. (2025). Mechanisms Linking Obesity with Non-Alcoholic Fatty Liver Disease (NAFLD) and Cardiovascular Diseases (CVDs)—The Role of Oxidative Stress. Curr. Issues Mol. Biol..

[4] Zheng L., Yang L., Guo Z., Yao N., Zhang S., Pu P. (2024). Obesity and its impact on female reproductive health: Unraveling the connections. Front. Endocrinol..

[5] Barber T.M., Hanson P., Weickert M.O., Franks S. (2019). Obesity and Polycystic Ovary Syndrome: Implications for Pathogenesis and Novel Management Strategies. Clin. Med. Insights Reprod. Health.

[6] Ouyang X., Zhou Q., Tang H., Li L. (2025). Pathogenesis and treatment of obesity-related polycystic ovary syndrome. J. Ovarian Res..

[7] Chao A.M., Taylor S., Moore M., Amaro A., Wadden T.A. (2024). Evolving Approaches for Pharmacological Therapy of Obesity. Annu. Rev. Pharmacol. Toxicol..

[8] Voros C., Chatzinikolaou F., Papapanagioutou I., Polykalas S., Mavrogianni D., Koulakmanidis A.-M., Athanasiou D., Kanaka V., Bananis K., Athanasiou A. (2026). A Systematic Review on GLP-1 Receptor Agonists in Reproductive Health: Integrating IVF Data, Ovarian Physiology and Molecular Mechanisms. Int. J. Mol. Sci..

[9] Chen Y., Wang R., Zhang N., Xu L. (2025). The dual burden of obesity: Decoding metabolism and female reproductive endocrinology. Front. Physiol..

[10] Shetty S., Bannur Karunakara M., Kristipati R.R., Kalthur G., Kumari S. (2015). Role of Liraglutide in weight management and reproductive health in women with obesity and PCOS-A critical review of the evidence. F1000Research.

[11] Sills E.S., Harrity C., Chu H.I., Wang J.-W., Yang F., Wood S.H. (2025). Semaglutide and human reproduction: Caution at the intersection of energy balance, ovarian function, and follicular development. Reprod. Biol. Endocrinol..

[12] Varra F.N., Varras M., Varra V.K., Theodosis-Nobelos P. (2026). Anti-obesity treatments with anti-inflammatory and antioxidant potential and their effects on obesity-related metabolic and cardiovascular disorders: A narrative review. Curr. Rev. Clin. Exp. Pharmacol..

[13] Niwińska K.E., Borowski M., Leśniak J.A., Leśniak N.M., Patrzykąt K.M., Zakrzewska A.M., Popielarska K., Michalak J.A., Augustyn M., Midera A. (2026). The Impact of GLP-1 Analogues and Tirzepatide on Female Fertility: Mechanisms, Clinical Evidence, and Implications. J. Educ. Health Sport.

[14] Cerón Saldívar H.I. (2023). Reproduction and Anti-Obesity Medications: A Review of Current Evidence. J. Reprod..

[15] Magzoub R., Kheirelseid E.A.H., Perks C., Lewis S. (2022). Does metformin improve reproduction outcomes for non-obese, infertile women with polycystic ovary syndrome? Meta-analysis and systematic review. Eur. J. Obstet. Gynecol. Reprod. Biol..

[16] Shpakov A.O. (2021). Improvement Effect of Metformin on Female and Male Reproduction in Endocrine Pathologies and Its Mechanisms. Pharmaceuticals.

[17] Devi Anala A., Hussain Saifudeen I.S., Ibrahim M., Nanda M., Naaz N., Atkin S.l. (2023). The Potential Utility of Tirzepatide for the Management of Polycystic Ovary Syndrome. J. Clin. Med..

[18] Goldberg A., Graca S., Liu J., Rao V., Witchel S.F., Pena A., Li R., Mousa A., Tay C.T., Pattuwage L. (2024). Anti-obesity pharmacological agents for polycystic ovary syndrome: A systematic review and meta-analysis to inform the 2023 international evidence-based guideline. Obes. Rev..

[19] Etrusco A., Mikuš M., D’Amato A., Barra F., Planinić P., Goluža T., Buzzaccarini G., Marušić J., Tešanović M., Laganà A.S. (2024). Incretin Hormone Secretion in Women with Polycystic Ovary Syndrome: Roles of Obesity, Insulin Sensitivity and Treatment with Metformin and GLP-1s. Biomedicines.

[20] Kyrou I., Randeva H.S., Tsigos C., Kaltsas G., Weickert M.O., Feingold K.R., Anawalt B., Blackman M.R., Boyce A., Chrousos G. (2018). Clinical problems caused by obesity. Endotext.

[21] Venkatesh S.S., Ferreira T., Benonisdottir S., Rahmioglu N., Becker C.M., Granne I., Zondervan K.T., Holmes M.V., Lindgren C.M., Wittemans L.B.L. (2022). Obesity and risk of female reproductive conditions: A mendelianl randomization study. PLoS Med..

[22] Bourebaba N., Ngo T.H., Śmieszek A., Bourebaba L., Marycz K. (2022). Sex hormone binding globulin as a potential drug candidate for liver-related metabolic disorders treatment. Biomed. Pharmacother..

[23] Blasco B.V., García-Jaménez J., Bodoano I., Gutiérrez-Rojas L. (2020). Obesity and depression: Its prevalence and influence as a prognostic factor: A systematic review. Psychiatry Investig..

[24] Manfredi-Lozano M., Roa J., Tena-Semrere M. (2018). Connecting metabolism and gonadal function: Novel central neuropeptide pathways involved in the metabolic control of puberty and fertility. Front. Neuroendocrinol..

[25] Huang A., Reinehr T., Roth C. (2020). Connections between obesity and puberty. Curr. Opin. Endocr. Metab. Res..

[26] Cena H., Chiovato L., Nappi R.E. (2020). Obesity, polycystic ovary syndrome, and infertility: A new avenue for GLP-1 receptor agonists. J. Clin. Endocrinol. Metab..

[27] Mahutte N., Kamga-Ngade C., Sharma A., Sylvestre C. (2018). Obesity and reproduction. J. Obstet. Gynecol. Can..

[28] Armstrong A., Berger M., Al-Safi Z. (2022). Obesity and reproduction. Curr. Opin. Obest Gynecol..

[29] Evans M.C., Lord R.A., Anderson G.M. (2021). Multiple leptin signaling pathways in the control of metabolism and fertility: A means to different ends?. Int. J. Mol. Sci..

[30] Hotkamp K., Mika C., Grzella I., Heer M., Pak H., Heberbrand J., Herpertz-Dahlmann B. (2003). Preproductive function during weight gain in anorexia revrosa. Leptin represents a metabolic gate to gonadotropin secretion. J. Neural Trasm..

[31] Westerman R., Kuhnt A.K. (2022). Metabolic risk factors and fertility disorders: A narrative review of the female perspective. Reprod. Biomed. Soc. Online.

[32] Pérez-Pérez A., Sánchez-Jiménez F., Maymó J., Dueñas J.L., Varone C., Sánchez-Margalet V. (2015). Role of leptin in female reproduction. Clin. Chem. Lab. Med..

[33] Bernardi L.A., Carnthon M.R., de Chavez P.J., Ikhena D.E., Neff L.M., Baird D.D., Marsh E.E. (2017). Relationship between obesity and anti-Müllerian hormone in reproductive-aged African-American women. Obesity.

[34] Merhi Z., Bazzi A.A., Bonney E.A., Buyuk E. (2019). Role of adiponectin in ovarian follicular development and ovarian reserve. Biomed. Rep..

[35] HogenEsch E., Boots C., Bernardi L.A. (2021). Aneuploidy rates are not higher in women with obesity: Is it worth the “weight” to delay in vitro fertilization until body mass index decreases?. Fertil. Steril..

[36] Pasquali R., Pelusi C., Genghini S., Cacciari M., Gambineri A. (2003). Obestity and reproductive disorders in women. Hum. Reprod. Update.

[37] Ostinelli G., Laforest S., Denham S.G., Gauthier M.F., Drolet-Labelle V., Scott E., Hould F.S., Marceau S., Homer N.Z.M., Bégin C. (2022). Increased adipose tissue indices of androgen catabolism and aromatization in women with metabolic dysfunction. J. Clin. Endocrinol. Metab..

[38] Barber T.M. (2022). Why are women with polycystic ovary syndrome obese?. Br. Med. Bull..

[39] Witchel S.F., Oberfield S.E., Peña A.S. (2019). Polycystic ovary syndrome: Pathophysiology, presentation, and treatment with emphasis on adolescent girls. J. Endocr. Soc..

[40] Jahromi B.N., Borzou N., Parsanezhad M.E., Envar Z., Ghaemmaghami P., Sebetian S. (2021). Associations of insulin resistance, sex hormone-binding globulin, triglyceride, and hormonal profiles in polycystic ovary syndrome: A cross-sectional study. Int. J. Reprod. Biomed..

[41] Xing C., Zhang J., Zhao H., He B. (2022). Effect of sex hormone-binding globulin on polycystic ovary syndrome: Mechanisms, manifestations, genetics and treatment. Int. J. Womens Health.

[42] Unluhizarci K., Karaca Z., Kelestimur F. (2021). Role of insulin and insulin resistance in androgen excess disorders. World J. Diabetes.

[43] Amiri M., Tehrani F.R. (2020). Potential adverse effects of female and male obesity on fertility: A narrative review. Int. J. Endocrinol. Metab..

[44] Yilmaz N., Kilic S., Kanat-Pektas M., Gykerman C., Mollamahmutoglu L. (2009). The relationship between obesity and fecundity. J. Womens Health.

[45] Van der Steeg J.W., Steures P., Eijkemans M.J., Habbema J.D., Hompes P.G., Burggraaff J.M., Oosterhuis G.J.E., Bossuyt P.M., van der Veen F., Mol B.W. (2008). Obesity affects spontaneous pregnancy changes in subfertility ovulatory women. Hum. Reprod..

[46] Narice B.F., Metwally M. (2020). Evidence-based assisted reproduction in obese women. Obes. Gynecol..

[47] Ikedionwu C.A., Dongarwar D., Yusuf K.K., Ibrahimi S., Salinas-Miranda A.A., Salihu H.M. (2020). Prepregnancy maternal obesity, macrosomia, and risk of stillbirth: A population-based study. Eur. J. Obstet. Gynecol. Reprod. Biol..

[48] Liu B., Xu G., Sun Y., Du Y., Gao R., Snetselaar L.G., Santillan M.K., Bao W. (2019). Association between maternal prepregnancy obesity and preterm birth according to maternal age and race or ethnicity: A population-based study. Lancet Diabetes Endocrinol..

[49] Olson K.N., Redman L.M., Sones J.L. (2019). Obestiy “complements” preeclampsia. Physiol. Genom..

[50] LeBlanc E.S., Smith N.X., Vesco K.K., A Hillier T., Stevens V.J. (2021). Weight loss prior to pregnancy and early gestational glycemia: Prepare, a randomized clinical trial. J. Clin. Endocrinol. Metab..

[51] Cavalcante M.B., Sarno M., Peixoto A.B., Júnior E.A., Barini R. (2019). Obesity and recurrent miscarriage: A systematic review and meta-analysis. J. Obstet. Gynecol. Res..

[52] Aydogan Mathyk B., Quaas A.M. (2021). Obesity and IVF: Weighing in on the evidence. J. Assist. Reprod. Genet..

[53] Shen X., Xie Y., Chen D., Guo W., Feng G., Jiang W., Long H., Lyu Q., Jin W., Kuang Y. (2021). Effect of female and male body mass index on cumulative live birth rates in the freeze-all strategy. J. Clin. Endocrinol. Metab..

[54] Gonzalez M.B., Rodker R.L., Rose R.D. (2022). Obestiy and oocyte quality: Significant implications for ART and emerging mechanistic insights. Biol. Reprod..

[55] Broughton D.E., Moley K.H. (2017). Obesity and female infertility: Potential mediators of obesity’s impact. Fertil. Steril..

[56] Lainez N.M., Coss D. (2019). Obesity, neuroinflammation, and reproductive function. Endocrinology.

[57] Li Q., Wang Q., Xu W., Ma Y., Wang Q., Eatman D., You S., Zou J., Champion J., Zhao L. (2020). C-reactive protein causes adult-onset obesity through chronic inflammatory mechanism. Front. Cell Dev. Biol..

[58] Herzberger E.H., Miller N., Ghetler Y., Yaniv R.T., Neumark E., Shulman A., Wiser A. (2021). A prospective study of C-reactive protein in patients with obesity during IVF. Hum. Fertil..

[59] Catalano P.M., Shankar K. (2017). Obesity and pregnancy: Mechanisms of short term and long term adverse consequences for mother and child. Br. Med. J..

[60] Gonzalez M.B., Lane M., Knight E.J., Bobker R.L. (2018). Inflammatory markers in human follicular fluid correlate with lipid levels and body mass index. J. Reprod. Immunol..

[61] Song J., Xiang S., Pang C., Guo J., Sun Z. (2020). Metabolomic alterations of follicular fluid of obese women undergoing in-vitro fertilization treatment. Sci. Rep..

[62] Balen A.H., Platteau P., Andersen A.N., Devroey P., Sørensen P., Helmgaard L., Arce J.C. (2006). The influence of body weight on response to ovulation induction with gonadotrophins in 335 women with World Health Organization group II anovulatory infertility. BJOG Int. J. Obstet. Gynaecol..

[63] Isa A.M., Abu-Rafea B., Alasiri S.A., Binsaleh S., Ismail K.H., Vilos G.A. (2014). Age, body mass index, and number of previous trials: Are they prognosticators of intra-uterine-insemination for infertility treatment?. Int. J. Fertil. Steril..

[64] Bellver J., Marín C., Lathi R.B., Murugappan G., Labarta E., Vidal C., Giles J., Cabanillas S., Marzal A., Galliano D. (2021). Obetity affects endometrial receptivity by displacing the window implantation. Reprod. Sci..

[65] Yang T., Zhao J., Liu F., Li Y. (2022). Lipid metabolism and endometrial receptivity. Hum. Reprod. Update.

[66] Wei S., Schmidt M.D., Dwyer T., Norman R.J., Venn A.J. (2009). Obesity and menstrual irregularity: Assocations with SHBG, testosterone, and insulin. Obesity.

[67] Nuako A., Tu L., Reyes K.J.C., Chhabria S.M., Standord F.C. (2023). Pharmacologic Treatment of Obesity in Reproductive Aged Women. Curr. Obstet. Gynecol. Rep..

[68] Duah J., Seifer D.B. (2025). Medical therapy to treat obesity and optimize fertility in women of reproductive age: A narrative review. Reprod. Biol. Endocrinol..

[69] Torgerson J.S., Hauptman J., Boldrin M.N., Sjöström L. (2004). XENical in the prevention of diabetes in obese subjects (XENDOS) study: A randomized study of orlistat as an adjunct to lifestyle changes for the prevention of type 2 diabetes in obese patients. Diabetes Care.

[70] Vosnakis C., Georgopoulos N.A., Rousso D., Mavromatidis G., Katsikis I., Roupas N.D., Mamali I., Panidis D. (2013). Diet, physical exercise and Orlistat administration increase serum anti-Mullerian hormone (AMH) levels in women with polycystic ovary syndrome (PCOS). Gynecol. Endocrinol..

[71] Kumar P., Arora S. (2014). Orlistat in polycystic ovarian syndrome reduces weight with improvement in lipid profile and pregnancy rates. J. Hum. Reprod. Sci..

[72] Tong J., Xiang L., Niu Y., Zhang T. (2022). Effect of orlistat intervention on in vitro fertilization/intracytoplasmic sperm injection outcome in overweight/obese infertile women. Gynecol. Endocrinol..

[73] Al-Qahwajy M.A.A., Eissa A.K.A., Taha W.S., Abdelmoaty M.A. (2022). QRLISTAT (the lipase inhibitor) therapy in overweight and obese sub-fertile women. Al-Azhar Med. J..

[74] Wang Z., Zhao J., Ma X., Sun Y., Hao G., Yang A., Ren W., Jin L., Lu Q., Wu G. (2021). Effect of Orlistat on Live Birth Rate in Overweight or Obese Women Undergoing IVF-ET: A Randomized Clinical Trial. J. Clin. Endocrinol. Metab..

[75] Legro R.S., Hansen K.R., Diamond M.P., Steiner A.Z., Coutifaris C., Cedars M.I., Hoeger K.M., Usadi R., Johnstone E.B., Haisenleder D.J. (2022). Effects of preconception lifestyle intervention in infertile women with obesity: The FIT-PLESE randomized controlled trial. PLoS Med..

[76] Várbíró S., Takács I., Tűű L., Nas K., Sziva R.E., Hetthéssy J.R., Török M. (2022). Effects of Vitamin D on Fertility, Pregnancy and Polycystic Ovary Syndrome-A Review. Nutrients.

[77] Pavli P., Triantafyllidou O., Kapantais E., Vlahos N.F., Valsamakis G. (2024). Infertility Improvement after Medical Weight Loss in Women and Men: A Review of the Literature. Int. J. Mol. Sci..

[78] Maselli D., Atieh H., Clark M.M., Eckert D., Taylor A., Carlson P., Burton D.D., Busciglio I., Harmsen W.S., Vella A. (2022). Effects of Liraglutide on Gastrointestinal Functions and Weight in Obesity: A Randomized Clinical and Pharmacogenomic Trial. Obesity.

[79] Pi-Sunyer X., Astrup A., Fujioka K., Greenway F., Halpern A., Krempf M., Lau D.C.W., Le Roux C.W., Ortiz R.V., Jensen C.B. (2015). A randomized, controlled trial of 3.0 mg of liraglutide in weight management. N. Engl. J. Med..

[80] Nylander M., Frossing S., Clausen H.V., Kistorp C., Faber J., Skouby S.O. (2017). Effects of liraglutide on ovarian dysfunction in polycystic ovary syndrome: A randomized clinical trial. Reprod. Biomed. Online.

[81] Salamun V., Jensterle M., Janez A., Bokal E.V. (2018). Liraglutide increases IVF pregnancy rates in obese PCOS women with poor response to first-line reproductive treatments: A pilot randomized study. Eur. J. Endocrinol..

[82] Knudsen L.B., Lau J. (2019). The Discovery and Development of Liraglutide and Semaglutide. Front. Endocrinol..

[83] Mahapatra M.K., Karuppasamy M., Sahoo B.M. (2022). Semaglutide, a glucagon like peptide-1 receptor agonist with cardiovascular benefits for management of type 2 diabetes. Rev. Endocr. Metab. Disord..

[84] Weiskirchen R., Lonardo A. (2025). Semaglutide from Bench to Bedside: The Experimental Journey Towards a Transformative Therapy for Diabetes, Obesity and Metabolic Liver Disorders. Med. Sci..

[85] Papakonstantinou I., Tsioufis K., Katsi V. (2024). Spotlight on the Mechanism of Action of Semaglutide. Curr. Issues Mol. Biol..

[86] Miles K.E., Kerr J.L. (2018). Semaglutide for the Treatment of Type 2 Diabetes Mellitus. J. Pharm. Technol..

[87] Salvador R., Moutinho C.G., Sousa C., Vinha A.F., Carvalho M., Matos C. (2025). Semaglutide as a GLP-1 Agonist: A Breakthrough in Obesity Treatment. Pharmaceuticals.

[88] Gao X., Hua X., Wang X., Xu W., Zhang Y., Shi C., Gu M. (2022). Efficacy and safety of semaglutide on weight loss in obese or overweight patients without diabetes: A systematic review and meta-analysis of randomized controlled trials. Front. Pharmacol..

[89] Knop F.K., Aroda V.R., Vale R.D.D., Holst-Hansen T., Laursen P.N., Rosenstock J., Rubino D.M., Garvey W.T., OASIS 1 Investigators (2023). Oral semaglutide 50 mg taken once per day in adults with overweight or obesity (OASIS 1): A randomised, double-blind, placebo-controlled, phase 3 trial. Lancet.

[90] Ferrara F., Bazzani D., Crivelli B., Danieli E., Gazzola P., Guarnieri G., Handschin G., Lauria C., Marchetti C., Sbraga E. (2025). Progress and challenges in obesity pharmacotherapy: Semaglutide as a milestone. Naunyn Schmiedebergs Arch. Pharmacol..

[91] Mandal L., Andersen L.U., Luef B.M., Tanvig M.H., Vinter C.A. (2026). Impact of semaglutide exposure on fetal and neonatal outcomes in pregnant women: A systematic review. Eur. J. Obstet. Gynecol. Reprod. Biol..

[92] Diab H., Fuquay T., Datta P., Bickel U., Thompson J., Krutsch K. (2024). Subcutaneous Semaglutide during Breastfeeding: Infant Safety Regarding Drug Transfer into Human Milk. Nutrients.

[93] Carmina E., Longo R.A. (2023). Semaglutide Treatment of Excessive Body Weight in Obese PCOS Patients Unresponsive to Lifestyle Programs. J. Clin. Med..

[94] Chen H., Lei X., Yang Z., Xu Y., Liu D., Wang C., Du H. (2025). Effects of combined metformin and semaglutide therapy on body weight, metabolic parameters, and reproductive outcomes in overweight/obese women with polycystic ovary syndrome: A prospective, randomized, controlled, open-label clinical trial. Reprod. Biol. Endocrinol..

[95] Merhi Z. (2026). GLP-1 receptor agonists and sexual function in women and men: A narrative review of emerging evidence and the need for further research. Sex. Med. Rev..

[96] Lau J., Bloch P., Schäffer L., Pettersson I., Spetzler J., Kofoed J., Madsen K., Knudsen L.B., McGuire J., Steensgaard D.B. (2015). Discovery of the Once-Weekly Glucagon-Like Peptide-1 (GLP-1) Analogue Semaglutide. J. Med. Chem..

[97] Nauck M.A., Quast D.R., Wefers J., Meier J.J. (2021). GLP-1 receptor agonists in the treatment of type 2 diabetes—State-of-the-art. Mol. Metab..

[98] Yang X.D., Yang Y.Y. (2024). Clinical Pharmacokinetics of Semaglutide: A Systematic Review. Drug Des. Devel Ther..

[99] La Vignera S., Condorelli R.A. (2026). Benefits of Incretin Therapy on Ovarian Function: A Scientific Literature Review. Int. J. Mol. Sci..

[100] Khera R., Murad M.H., Chandar A.K., Dulai P.S., Wang Z., Prokop L.J., Loomba R., Camilleri M., Singh S. (2016). Association of pharmacological treatments for obesity with weight loss and adverse events: A systematic review and meta-analysis. J. Am. Med. Assoc..

[101] Billes S.K., Sinnayah P., Cowley M.A. (2014). Naltrexone/bupropion for obesity: An investigational combination pharmacotherapy for weight loss. Pharmacol. Res..

[102] Gadde K.M., Parker C.B., Maner L.G., Wagner H.R., Logue E.J., Drezner M.K., Krishnan K.R.R. (2001). Bupropion for weight loss: An investigation of efficacy and tolerability in overweight and obese women. Obes. Res..

[103] Fulton S., Décarie-Spain L., Fioramonti X., Guiard B., Nakajima S. (2022). The menace of obesity to depression and anxiety prevalence. Trends Endocrinol. Metab..

[104] Abdalla M.A., Deshmukh H., Atkin S., Sathyapalan T. (2020). A review of therapeutic options for managing the metabolic aspects of polycystic ovary syndrome. Ther. Adv. Endocrinol. Metab..

[105] Ahmed M.I., Duleba A.J., El Shahat O., Ibrahim M.E., Salem A. (2008). Naltrexone treatment in clomiphene resistant women with polycystic ovary syndrome. Hum. Reprod..

[106] Fulghesu A.M., Ciampelli M., Belosi C., Apa R., Guido M., Caruso A., Mancuso S., Lanzoneal A. (2001). Naltrexone effect on pulsatile GnRH therapy for ovulation induction in polycystic ovary syndrome: A pilot prospective study. J. Endocrinol. Investig..

[107] Wadden T.A., Foreyt J.P., Foster G.D., Hill J.O., Klein S., O’Neil P.M., Perri M.G., Pi-Sunyer F.X., Rock C.L., Erickson J.S. (2011). Weight loss with naltrexone SR/bupropion SR combination therapy as an adjunct to behavior modification: The COR-BMOD trial. Obesity.

[108] Apovian C.M., Aronne L., Rubino D., Still C., Wyatt H., Burns C., Kim D., Dunayevich E., COR-II Study Group (2013). A randomized, phase 3 trial of naltrexone SR/bupropion SR on weight and obesity-related risk factors (COR-II). Obesity.

[109] Hollander P., Gupta A.K., Plodkowski R., Greenway F., Bays H., Burns C., Klassen P., Fujioka K., COR-Diabetes Study Group (2013). Effects of naltrexone sustained-release/bupropion sustained-release combination therapy on body weight and glycemic parameters in overweight and obese patients with type 2 diabetes. Diabetes Care.

[110] Liu Y., Han F., Xia Z., Sun P., Rohani P., Amirhalingam P., Sohouli M.H. (2024). The effects of bupropion alone and combined with naltrexone on weight loss: A systematic review and meta-regression analysis of randomized controlled trials. Diabetol. Metab. Syndr..

[111] Yuan L., Wu H., Huang W., Bi Y., Qin A., Yang Y. (2021). The function of metformin in endometrial receptivity (ER) of patients with polycyclic ovary syndrome (PCOS): A systematic review and meta-analysis. Reprod. Biol. Endocrinol..

[112] Diamanti-Kandarakis E., Christakou C.D., Kandaraki E., Economou F.N. (2010). Metformin: An old medication of new fashion: Evolving new molecular mechanisms and clinical implications in polycystic ovary syndrome. Eur. J. Endocrinol..

[113] Kai Y., Kawano Y., Yamamoto H., Narahara H. (2015). A possible role for AMP-activated protein kinase activated by metformin and AICAR in human granulosa cells. Reprod. Biol. Endocrinol..

[114] Feure M., Bertoldo M.J., Khoueiry R., Bongrani A., Brion F., Giulivi C., Dupont J., Froment P. (2018). Metformin in Reproductive Biology. Front. Endocrinol..

[115] Mahamed R.R., Maganhin C.C., Sasso G.R.S., de Jesus Simões M., Baracat M.C.P., Baracat E.C., Soares J.M. (2018). Metformin improves ovarian follicle dynamics by reducing theca cell proliferation and CYP-17 expression in an androgenized rat model. J. Ovarian Res..

[116] Xiao N., Wang J., Wang T., Xiong X., Zhou J., Su X., Peng J., Yang C., Li X., Lin G. (2022). Metformin abrogates pathological TNF-α-producing B cells through mTOR-dependent metabolic reprogramming in polycystic ovary syndrome. eLife.

[117] Ullah A., Chen Y., Zhang F., Shen B. (2026). Beyond glycemic control: Molecular mechanisms of metformin in modulating cytokine networks in polycystic ovary syndrome. Front. Endocrinol..

[118] Zhai J., Yao G.-D., Wang J.-Y., Yang Q.-L., Wu L., Chang Z.-Y., Sun Y.-P. (2019). Metformin Regulates Key MicroRNAs to Improve Endometrial Receptivity Through Increasing Implantation Marker Gene Expression in Patients with PCOS Undergoing IVF/ICSI. Reprod. Sci..

[119] Kimber-Trojnar Ż., Dłusk D.F., Wierzchowska-Opoka M., Ruszała M., Leszczyńska-Gorzelak B. (2022). Metformin as a Potential Treatment Option for Endometriosis. Cancers.

[120] Nie P., Wang M., Mo Y., Zhou H., Zha Q., Lash G.E., Li P. (2025). Metformin in gynecological disorders: Pathogenic insights and therapeutic implications. Front. Pharmacol..

[121] Cheng J., Li C., Ying Y., Lv J., Qu X., McGowan E., Lin Y., Zhu X. (2022). Metformin Alleviates Endometriosis and Potentiates Endometrial Receptivity via Decreasing VEGF and MMP9 and Increasing Leukemia Inhibitor Factor and HOXA10. Front. Pharmacol..

[122] Palomba S., Falbo A., La Sala G.B. (2013). Effects of metformin in women with polycystic ovary syndrome treated with gonadotrophins for in vitro fertilisation and intracytoplasmic sperm injection cycles: A systematic review and meta-analysis of randomised controlled trials. BJOG Int. J. Obstet. Gynaecol..

[123] Morin-Paunen L., Rantala A.S., Unkila-Kallio L., Titinen A., Hippeläinen M., Perheentupa A., Tinkanen H., Bloigu R., Puukka K., Ruokonen A. (2012). Metformin improves pregnancy and live-birth rates in women with polycystic ovary syndrome (PCOS): A multicenter, double-blind, placebo-controlled randomized trial. J. Clin. Endocrinol. Metab..

[124] Lentferink Y.E., Knibbe C.A.J., Van Der Vorst M.M.J. (2018). Efficacy of metformin treatment with respect to weight reduction in children and adults with obesity: A systematic review. Drugs.

[125] Jensterle M., Kravos N.A., Goriĉar K., Janez A. (2017). Short-term effectiveness of low dose liraglutide in combination with metformin versus high dose liraglutide alone in treatment of obese PCOS: Randomized trial. BMC Endocr. Disord..

[126] Tso L.O., Costello M.F., Albuquerque L.E.T., Andriolo R.B., Marjoribanks J., MacEdo C.R. (2015). Metformin treatment before and during in vitro fertilization or intracytoplasmic sperm injection in women with polycystic ovary syndrome: Summary of a cochrane review. Fertil. Steril..

[127] Abdalmageed O.S., Farghaly T.A., Abdelaleem A.A., Abdelmagied A.E., Ali M.K., Abbas A.M. (2019). Impact of Metformin on IVF Outcomes in Overweight and ObeseWomenWith Polycystic Ovary Syndrome: A Randomized Double-Blind Controlled Trial. Reprod. Sci..

[128] Misso M.L., Costello M.F., Garrubba M., Wong J., Hart R., Rombauts L., Melder A.M., Norman R.J., Teede H.J. (2013). Metformin versus clomiphene citrate for infertility in non-obese women with polycystic ovary syndrome: A systematic review and meta-analysis. Hum. Reprod..

[129] Agrawal A., Mahey R., Kachhawa G., Khadgawat R., Vanamail P., Kriplani A. (2019). Comparison of metformin plus myoinositol vs metformin alone in PCOS women undergoing ovulation induction cycles: Randomized controlled trial. Gynecol. Endocrinol..

[130] Dukhovny S., Van Bennekom C.M., Gagnon D.R., Hernandez Diaz S., Parker S.E., Anderka M., Werler M.M., Mitchell A.A. (2018). Metformin in the first trimester and risks for specific birth defects in the National Birth Defects Prevention Study. Birth Defects Res..

[131] Notaro A.L.G., Neto F.T.L. (2022). The use of metformin in women with polycystic ovary syndrome: An updated review. J. Assist. Reprod. Genet..

[132] Tran K.L., Park Y.I., Pandya S., Muliyil N.J., Jensen B.D., Huyh K., Nguyen Q.T. (2017). Overview of Glucagon-Like Peptide-1 Receptor Agonists for the Treatment of Patients with Type 2 Diabetes. Am. Health Drug Benefits.

[133] Khun Yap M.K., Misuan N. (2019). Exendin-4 from Heloderma suspectum venom: From discovery to its latest application as type II diabetes combatant. Basic Clin. Pharmacol. Toxicol..

[134] Underwood C.R., Garibay P., Knudsen L.B., Hartrup S., Peters G.H., Rudolph R., Reedtz-Runge S. (2009). Crystal Structure of Glucagon-like Peptide-1 in Complex with the Extracellular Domain of the Glucagon-like Peptide-1 Receptor. J. Biol. Chem..

[135] Fehse F., Trautmann M., Holst J.J., Halseth A.E., Nanayakkara N., Nielsen L.L., Fineman M.S., Kim D.D., Nauck M.A. (2005). Exenatide augments first- and second-phase insulin secretion in response to intravenous glucose in subjects with type 2 diabetes. J. Clin. Endocrinol. Metab..

[136] Voronova V., Zhudenkov K., Pendand R.C., Boulton D.W., Helmlinger G., Peskov K. (2018). Exenatide effects on gastric emptying rate and the glucose rate of appearance in plasma: A quantitative assessment using an integrative systems pharmacology model. Diabetes Obes. Metab..

[137] van Bloemendaal L., Ten Kulve J.S., la Fleur S.E., Ijzerman R.G., Diamant M. (2014). Effects of glucagon-like peptide 1 on appetite and body weight: Focus on the CNS. J. Endocrinol..

[138] Segal J.B., Dy S.M., Millman E.A., Herbert R., Bass E.B., Wu A. (2007). Diffusion into use of exenatide for glucose control in diabetes mellitus: A retrospective cohort study of a new therapy. Clin. Ther..

[139] Bridges A., Bistas K.G., Jacobs T.F. (2023). Exenatide. StatPearls.

[140] McCormack P.L. (2014). Exenatide twice daily: A review of its use in the management of patients with type 2 diabetes mellitus. Drugs.

[141] Hu Y., Son X., Hamiti S., Ma Y., Yusufu M., Wang X., Zhang K., Guo Y. (2023). Comparison of exenatide alone or combined with metformin versus metformin in the treatment of polycystic ovaries: A systematic review and meta-analysis. BMC Endocr. Disord..

[142] Rosenstock J., Klaff L.J., Schwartz S., Northrup J., Holcombe J.H., Wilhelm K., Trautmann M. (2010). Effects of exenatide and lifestyle modification on body weight and glucose tolerance in obese subjects with and without pre-diabetes. Diabetes Care.

[143] Lundkvist P., Sjöström C.D., Amini S., Pereira M.J., Johnsson E., Eriksson J.W. (2017). Dapagliflozin once-daily and exenatide once-weekly dual therapy: A 24-week randomized, placebo-controlled, phase II study examining effects on body weight and prediabetes in obese adults without diabetes. Diabetes Obes. Metab..

[144] Basolo A., Burkholder J., Osgood K., Graham A., Bundrick S., Frankl J., Piaggi P., Thearle M.S., Krakoff J. (2018). Exenatide has a pronounced effect on energy intake but not energy expenditure in nondiabetic subjects with obesity: A randomized, double-blind, placebocontrolled trial. Metabolism.

[145] Ma R.-L., Deng Y., Wang Y.-F., Zhu S.-Y., Ding X.-S., Sun A.-J. (2021). Short-Term Combined Treatment with Exenatide and Metformin for Overweight/Obese Women with Polycystic Ovary Syndrome. Chin. Med. J..

[146] van Ruiten C.C., Veltman D.J., Nieuwdorp M., IJzerman R.G. (2022). Brain Activation in Response to Low-Calorie Food Pictures: An Explorative Analysis of a Randomized Trial with Dapagliflozin and Exenatide. Front. Endocrinol..

[147] Liu X., Zhang Y., Zheng S.-Y., Lin R., Xie Y.-J., Chen H., Zheng Y., Liu E., Chen L., Yan J. (2017). Efficacy of exenatide on weight loss, metabolic parameters and pregnancy in overweight/obese polycystic ovary syndrome. Clin. Endocrinol..

[148] Ye Z.-R., Yan C.-Q., Liao N., Wen S.-H. (2023). The Effectiveness and Safety of Exenatide Versus Metformin in Patients with Polycystic Ovary Syndrome: A Meta-Analysis of Randomized Controlled Trials. Reprod. Sci..

[149] Li R., Mai T., Zheng S., Zhang Y. (2022). Effect of metformin and exenatide on pregnancy rate and pregnancy outcomes in overweight or obese infertility PCOS women: Long-term follow-up of an RCT. Arch. Gynecol. Obstet..

[150] Karagiannis T., Avgerinos I., Liakos A., Prato S.D., Matthews D.R., Tsapas A., Bekiari E. (2022). Management of type 2 diabetes with the dual GIP/GLP-1 receptor agonist tirzepatide: A systematic review and meta-analysis. Diabetologia.

[151] Nauck M.A., D‘Alessio D.A. (2022). Tirzepatide, a dual GIP/GLP-1 receptor co-agonist for the treatment of type 2 diabetes with unmatched effectiveness regrading glycaemic control and body weight reduction. Cardiovasc. Diabetol..

[152] Cho Y.K., Lee Y.L., Jung C.H. (2023). The Cardiovascular Effect of Tirzepatide: A Glucagon-Like Peptide-1 and Glucose-Dependent Insulinotropic Polypeptide Dual Agonist. J. Lipid Atheroscler..

[153] Alsalim W., Lindgren O., Ahrén B.A. (2023). Glucose-dependent insulinotropic polypeptide and glucagon-like peptide-1 secretion in humans: Characteristics and regulation. J. Diabetes. Investig..

[154] Frías J.P., Davies M.J., Rosenstock J., Pérez Manghi F.C., Fernández Landó L., Bergman B.K., Liu B., Cui X., Brown K., SURPASS-2 Investigators (2021). Tirzepatide versus Semaglutide Once Weekly in Patients with Type 2 Diabetes. N. Engl. J. Med..

[155] Tchang B.G., Mihai A.C., Stefanski A., García-Pérez L.-E., Mojdami D., Jouravskaya I., Gurbuz S., Taylor R., Karanikas C.A., Dunn J.P. (2025). Body weight reduction in women treated with tirzepatide by reproductive stage: A post hoc analysis from the SURMOUNT program. Obesity.

[156] Muller D.R.P., Stenvers D.J., Malekzadeh A., Holleman F., Painter R.C., Siegelaar S.E. (2023). Effects of GLP-1 agonists and SGLT2 inhibitors during pregnancy and lactation on offspring outcomes: A systematic review of the evidence. Front. Endocrinol..

[157] Varughese M.S., O’Mahony F., Varadhan L. (2025). GLP-1 receptor agonist therapy and pregnancy: Evolving and emerging evidence. Clin. Med..

[158] Ozbek L., Shah E., Al-Shiab R., Inal A., Guldan M., Afsar B., Covic A., Kanbay M. (2026). Safety of GLP-1 and Dual GLP-1/GIP Receptor Agonists in Preconception, Pregnancy, and Lactation: A Systematic Review of Maternal, Fetal, and Neonatal Outcomes. Diabetes Obes. Metab..

[159] Ziyadeh F., Saliba S., Bacha D.S., Mauer Y., Griebeler M.L., Burguera B. (2006). Update on antiobesity pharmacotherapy in adults: Current and emerging options. Clevel. Clin. J. Med..

[160] Morin-Papunen L., Vauhkonen I., Koivunen R., Ruokonen A., Martikainen H., Tapanainen J.S. (2003). Metformin versus ethinyl estradiol-cyproterone acetate in the treatment of nonobese women with polycystic ovary syndrome: A randomized study. J. Clin. Endocrinol. Metab..

[161] Diamanti-Kandarakis E., Economou F., Palimeri S., Christakou C. (2010). Metformin in polycystic ovary syndrome. Ann. N. Y. Acad. Sci..

[162] Nestler J.E. (2008). Metformin for the treatment of the polycystic ovary syndrome. N. Engl. J. Med..

[163] Palomba S., Falbo A., Zullo F., Orio F. (2009). Evidence-based and potential benefits of metformin in the polycystic ovary syndrome: A comprehensive review. Endocr. Rev..

[164] Wilding J.P.H., Batterham R.L., Calanna S., Davies M., Van Gaal L.F., Lingrav I., McGowan B.M., Rosenstock J., Tran M.T., Wadden T.A. (2021). Once-Weekly Semaglutide in Adults with Overweight or Obesity. N. Engl. J. Med..

[165] Couldwell M., Tidwell A.J., Taylor A.E. (2025). Effect of GLP1 Agonists on Reproduction. J. Clin. Endocrinol. Metab..

[166] Malhotra R., Garcia de Paredes J., Smith A., Chemrinski A., Doshi D., Morelli S.S. (2025). Obesity Epidemic and Its Impact on Female Fertility: Current Understanding and Future Directions. Cureus.

[167] Andreas E., Winstanley Y.E., Robker R.L. (2021). Effect of obesity on the ovarian follicular environment and developmental competence of the oocyte. Curr. Opin. Endocr. Metab. Res..

[168] Shi M., Sirard M.-A. (2022). Metabolism of fatty acids in follicular cells, oocytes, and blastocysts. Reprod. Fertil..

[169] Meulders B., Marei W.F.A., Loier L., Leroy L.M.R. (2025). Lipotoxicity and Oocyte Quality in Mammals: Pathogenesis, Consequences, and Reversibility. Annu. Rev. Anim. Biosci..

[170] Roberts R., Markande A., Kasaven L., Williams S.C., Faris R., Bracewell-Milnes T., Thum Y., Nicopoullos J., Jones B.P. (2026). Obesity and Female Reproductive Health; Is There a Role for Glucagon-Like Peptide-1 Receptor Agonists?. Obes. Rev..

[171] Luna-Marco C., de Marañon A.M., Hermo-Argibay A., Rodriguez-Hernandez Y., Hermenejildo J., Fernandez-Reyes M., Apostolova N., Vila J., Sola E., Morillas C. (2023). Effects of GLP-1 receptor agonists on mitochondrial function, inflammatory markers and leukocyte-endothelium interactions in type 2 diabetes. Redox Biol..

[172] Reppo I., Jakobson M., Volke V. (2023). Effects of Semaglutide and Empagliflozin on Inflammatory Markers in Patients with Type 2 Diabetes. Int. J. Mol. Sci..

[173] Tavares A.C.M., Martins M.Y.M., de Souza G.F., Lima E.M., Rocha C.A., de Souza L.C., Simões J.M.L., de Araújo N.O., Cavalcante M.B. (2025). Immunological effects of GLP-1 analogs on female reproduction: Therapeutic perspectives for infertility and recurrent pregnancy loss. J. Reprod. Immunol..

[174] Jastreboff A.M., Aronne L.J., Ahmad N.N., Wharton S., Connery L., Alves B., Kiyosue A., Zhang S., Liu B., Bunck M.C. (2022). Tirzepatide Once Weekly for the Treatment of Obesity. N. Engl. J. Med..

[175] Aamir A.B., Latif R., Alqoofi J.F., Almazoq F.A., Fallatah J.O., Hassan G.A., Al Abu Saab F.A.A. (2025). Comparative Efficacy of Tirzepatide vs. Semaglutide in Reducing Body Weight in Humans: A Systematic Review and Meta-Analysis of Clinical Trials and Real-World Data. J. Clin. Med. Res..

[176] Srivastava G., Apovian C.M. (2018). Current pharmacotherapy for obesity. Nat. Rev. Endocrinol..

[177] Ghandi S., Aflatoonian A., Tabibnejad N., Sojoodi Moghaddam M.H. (2011). The effects of metformin or orlistat on obese women with polycystic ovary syndrome: A prospective randomized open-label study. J. Assist. Reprod. Genet..

[178] Forslund M., Wändell P., Forsberg L., Österberg M., Dagerhamn J., Wernersson E., Fredriksson M.K., Ringborg A., Hirschberg A.L. (2026). GLP-1 receptor agonist treatment in women with polycystic ovary syndrome—A systematic review and meta-analysis. Eur.J. Endocrinol..

[179] Gonnella F., Konstantinidou F., Donato M., Gatta D.M.P., Peserico A., Barboni B., Stuppia L., Nothnick W.B., Gatta V. (2024). The Molecular Link between Obesity and the Endometrial Environment: A Starting Point for Female Infertility. Int. J. Mol. Sci..

[180] Greenway F.L., Fujioka K., Plodkowski R.A., Mudaliar S., Guttaauria M., Erickson J., Kim D.D., Dunayevich E. (2010). Effect of naltrexone plus bupropion on weight loss in overweight and obese adults (COR-I): A multicentre, randomised, double-blind, placebo-controlled, phase 3 trial. Lancet.

[181] Chatzis P., Tziomalos K., Pratilas G.C., Makris V., Sotiriadis A., Dinas K. (2018). The Role of Antiobesity Agents in the Management of Polycystic Ovary Syndrome. Folia Medica.

[182] Koufakis T., Busetto L. (2026). From one-size-fits-all to phenotype-based pharmacotherapy: How far are we in obesity management?. Curr. Opin. Pharmacol..

[183] Steenackers N., Toumassian J., Deleus E., Mertens A., Lannoo M., Pazmino S., van Laar A.D.E., Van der Schueren B., Vangoitsenhoven R. (2025). Pharmacotherapy for obesity: Are we ready to select, tailor and combine pharmacotherapy to achieve more ambitious goals?. Front. Endocrinol..

[184] Tuccinardi D., Masi D., Watanabe M., Buffi V.Z., De Domenico F., Berti S., Cipriani V., Manco M., Manfrini S., Pagotto U. (2025). Precision obesity medicine: A phenotype-guided framework for pharmacologic therapy across the lifespan. J. Endocrinol. Investig..

[185] Prosperi S., Chiarelli F. (2025). Insulin resistance, metabolic syndrome and polycystic ovaries: An intriguing conundrum. Front. Endocrinol..

[186] Teede H.J., Tay C.T., Laven J.J.E., Dokras A., Moran L.J., Pitonen T.T., Costello M.F., Boivin J., Redman L.M., A Boyle J. (2023). Recommendations from the 2023 International Evidence-based Guideline for the Assessment and Management of Polycystic Ovary Syndrome. J. Clin. Endocrinol. Metab..

[187] Liu S., Wang R., Yu W., Shi C., Wang X., Liu A., Zhang L. (2026). Natural Products in the Metabolic and Endocrine Modulation of Polycystic Ovary Syndrome: Current Perspectives. Nutrients.

[188] Hoteit B.H., Kotaich J., Ftouni H., Hazime F., Safawi A., Masri R., Marwani M. (2025). The dual impact of GLP-1 receptor agonists on metabolic and reproductive health in polycystic ovary syndrome: Insights from human and animal trials. Ther. Adv. Endocrinol. Metab..

[189] Matuszewski W., Wołos- Kłosowicz K., Włodarczyk P., Waśniewska P., Modzelewski R., Górny J.M., Szklarz M., Madeksza M., Juranek J. (2026). Beyond Glycemic Control: GLP-1RA–Based Therapies and Emerging Targets Beyond the Metabolic Axis. J. Clin. Med..

[190] Ling J., Wang T., Huang W., Zhen Y., Zhang M., Fang X., Song W., Du X. (2025). Combined liraglutide and metformin therapy in overweight or obese women with polycystic ovary syndrome: A systematic review and meta-analysis. Diabetes Obes. Metab..

[191] Acosta A., Camilleri M., Dayyeh B.A., Calderon G., Gonzalez D., McRae A., Rossini W., Singh S., Burton D., Clark M.M. (2021). Selection of Antiobesity Medications Based on Phenotypes Enhances Weight Loss: A Pragmatic Trial in an Obesity Clinic. Obesity.

[192] Gregor M.F., Hotamisligil G.S. (2011). Inflammatory mechanisms in obesity. Annu. Rev. Immunol..

[193] Tek C. (2016). Naltrexone HCI/bupropion HCI for chronic weight management in obese adults: Patient selection and perspectives. Patient Prefer. Adherence.

[194] Jeong H.G., Chao S., Ryu K.-J., Kim T., Park H. (2024). Effect of weight loss before in vitro fertilization in women with obesity or overweight and infertility: A systematic review and meta-analysis. Sci. Rep..

[195] Practice Committee of the American Society for Reproductive Medicine (2021). Obesity and reproduction: A committee opinion. Fertil. Steril..

[196] Alfaiz A.S. (2025). GLP-1 receptor agonists and preconception planning: Bridging the gap between obesity treatment and reproductive safety, a narrative review. Ann. Med. Surg..

[197] Frederick T.W., Camilleri M., Acosta A. (2025). Pharmacotherapy for Obesity: Recent Updates. Clin. Pharmacol..

[198] Abedi M.M., Patni M.M., Shajahan A.N.B., Dube R., Khadeeja L., Alabid I., Kharoufeh A., Kar S.S., George B.T., Bahutair S.N. (2026). GLP-1 Receptor Agonists, Fertility Restoration, and Reproductive Safety in Women of Reproductive Age: A Narrative Review. J. Clin. Med..

[199] Wang Z., Van Faassen M., Groen H., Cantineau A.E.P., Van Oers A., Van der Veen A., Hawley J.M., Keevil B.G., Kema I.P., Hoek A. (2024). Resumption of ovulation in anovulatory women with PCOS and obesity is associated with reduction of 11β-hydroxyandrostenedione concentrations. Hum. Reprod..

[200] Parker C.H., Slattery C., Brennan D.J., le Roux C.W. (2025). Glucagon-like peptide 1 (GLP-1) receptor agonists’ use during pregnancy: Safety data from regulatory clinical trials. Diabetes Obes. Metab..

[201] Blundell J., Finlayson G., Axelsen M., Flint A., Gibbons C., Kvist T., Hjerpsted J.B. (2017). Effects of once-weekly semaglutide on appetite, energy intake, control of eating, food preference and body weight in subjects with obesity. Diabetes Obes. Metab..

[202] Budini B., Luo B., Tam M., Stead I., Lee A., Akrami A., Vidal-Puig A., Park A. (2026). Trajectory of weight regain after cessation of GLP-1 receptor agonists: A systematic review and nonlinear meta-regression. eClinicalMedicine.

[203] Wan K.-W., Dai Z.-H., Lei E.F.C., Wong P.-S., Wang Y.-Y., Yu A.P., Lin F.-C., Huang W.Y., Duncane W.C., Chan K.Y. (2026). Effects of pharmacological, lifestyle, and combined interventions on body composition and metabolic health in women with excess adiposity and polycystic ovary syndrome: A systematic review and network meta-analysis of randomised controlled trials. Lancet Obstet. Gynaecol. Womens Health.

[204] Kettner J., Donnelly E., Maes M. (2026). Glucagon-like Peptide-1 Receptor Agonists and Reproductive Health: Current Evidence and Clinical Implications. J. Pharm. Pract..

[205] Zipursky J.S., Bogler T., Maxwell C. (2024). Glucagon-like peptide-1 receptor agonists during pregnancy and lactation. Can. Med. Assoc. J..

[206] Metzger B.E., Gabbe S.G., Persson B., Buchanan T.A., Catalano P.A., Damm P., Dyer A.R., de Leiva A., Hod M., International Association of Diabetes and Pregnancy Study Groups Consensus Panel (2010). International association of diabetes and pregnancy study groups recommendations on the diagnosis and classification of hyperglycemia in pregnancy. Diabetes Care.

[207] Almutairi F.S., Alsaykan A.M., Almatrood A.A. (2024). Obesity Prevalence and Its Impact on Maternal and Neonatal Outcomes in Pregnant Women: A Systematic Review. Cureus.

[208] ElSayed N.A., McCoy R.G., Aleppo G., Balapattabi K., Beverly E.A., Early K.B., Bruemmer D., Ebekozien O., Echouffo-Tcheugui J.B., American Diabetes Association Professional Practice Committee (2025). 2. Diagnosis and Classification of Diabetes: Standards of Care in Diabetes-2025. Diabetes Care.

[209] Fleming T.P., Watkins A.J., Velazquez M.A., Mathers J.C., Prentice A.M., Stephenson J., Barker M., Saffery R., Yajnik C.S., Eckert J.J. (2018). Origins of lifetime health around the time of conception: Causes and consequences. Lancet.

[210] Lane M., Rodker R., Robertson S.A. (2014). Parenting from before conception. Science.

[211] Zhang X.-G., Yang L.-L., Feng X., Zhang Y.W., Guo L., Huang S.X., Meng T.-G., Li P.-Y., Chen L.-N., Su R.-B. (2026). Maternal obesity disrupts epigenetic reprogramming via peroxisomal-dependent phospholipid-methyl uncoupling during zygotic genome activation. Nat. Commun..

[212] Hinte L.C., Castellano-Castillo D., Ghosh A., Melrose K., Gasser E., Noé F., Massier L., Dong H., Sun W., Hoffmann A. (2024). Adipose tissue retains an epigenetic memory of obesity after weight loss. Nature.

[213] Woldemariam S., Dorner T.E., Wiesinger T., Stein K.V. (2023). Multi-omics approaches for precision obesity management. Wien. Klin. Wochenschr..

[214] Jensterle M., Janez A., Fliers E., DeVries J.H., Vrtacnik-Bokal E., Siegellar S.E. (2019). The role of glucagon-like peptide-1 in reproduction: From physiology to therapeutic perspective. Hum. Reprod. Update.

[215] Fuentes-Mendoza J.M., Concepción-Zavaleta M.J., Mendoza-Godoy J.J., Concepción-Urteaga L., Paz-Ibarra J., Coronado-Arroyo J.C. (2026). Beyond metabolism: Sexual dysfunction and weight-loss drugs. Sex. Med. Rev..

[216] Bakken I.J., Ruiz P.L., Furu K., Gulseth H.L., Sveen K.A., Nøkleby K., Meyer H.E., Kjerpeseth L.J., Karlstad Ø. (2026). Clinical Characteristics of Users of Weight Loss Drugs: Population-Based Case-Control Study. Diabetes Obes. Metab..

[217] Pérez López G. (2025). GLP-1 receptor agonists and GIP/GLP-1 co-agonists in the treatment of obesity in adolescents and the elderly. Med. Clin..

[218] Thomsen R.W., Mailhac A., Løhde J.B., Pottegård A. (2025). Real-world evidence on the utilization, clinical and comparative effectiveness, and adverse effects of newer GLP-1RA-based weight-loss therapies. Diabetes Obes. Metab..

[219] Legro R.S., Barnhart H.X., Schlaff W.D., Carr B.R., Diamond M.P., Carson S.A., Steinkampf M.P., Coutifaris C., McGovern P.G., Cataldo N.A. (2007). Clomiphene, Metformin, or Both for Infertility in the Polycystic Ovary Syndrome. N. Engl. J. Med..

[220] Elkind-Hirsch K., Marrioneaux O., Bhushan M., Vernor D., Bhushan R. (2008). Comparison of Single and Combined treatment with exenatide and metformin on menstrual cyclicity in overweight women with polycystic ovary syndrome. J. Clin. Endocrinol. Metab..

[221] Wadden T.A., Chao A.M., Machineni S., Kushner R., Ard J., Srivastava G., Halpern B., Zhang S., Chen J., Bunck M.C. (2023). Tirzepatide after intensive lifestyle intervention in adults with overweight or obesity: The SURMOUNT-3 phase 3 trial. Nat. Med..

[222] Aronne L.J., Sattar N., Horn D.B., Bays H.E., Wharton S., Lin W.-Y., Ahmad N.N., Zhang S., Liao R., Bunck M.C. (2024). Continued Treatment with Tirzepatide for Maintenance of Weight Reduction in Adults with Obesity: The SURMOUNT-4 Randomized Clinical Trial. J. Am. Med. Assoc..

